# Rising levels of atmospheric oxygen and evolution of Nrf2

**DOI:** 10.1038/srep27740

**Published:** 2016-06-14

**Authors:** Ranko Gacesa, Walter C. Dunlap, David J. Barlow, Roman A. Laskowski, Paul F. Long

**Affiliations:** 1 Institute of Pharmaceutical Science, King’s College London, United Kingdom; 2 European Bioinformatics Institute, Wellcome Genome Campus, Cambridge, United Kingdom; 3 Department of Chemistry, King’s College London, United Kingdom

## Abstract

In mammals, the master transcription regulator of antioxidant defences is provided by the Nrf2 protein. Phylogenetic analyses of Nrf2 sequences are used here to derive a molecular clock that manifests persuasive evidence that Nrf2 orthologues emerged, and then diverged, at two time points that correlate with well-established geochemical and palaeobiological chronologies during progression of the ‘Great Oxygenation Event’. We demonstrate that orthologues of Nrf2 first appeared in fungi around 1.5 Ga during the Paleoproterozoic when photosynthetic oxygen was being absorbed into the oceans. A subsequent significant divergence in Nrf2 is seen during the split between fungi and the Metazoa approximately 1.0–1.2 Ga, at a time when oceanic ventilation released free oxygen to the atmosphere, but with most being absorbed by methane oxidation and oxidative weathering of land surfaces until approximately 800 Ma. Atmospheric oxygen levels thereafter accumulated giving rise to metazoan success known as the Cambrian explosion commencing at ~541 Ma. Atmospheric O_2_ levels then rose in the mid Paleozoic (359–252 Ma), and Nrf2 diverged once again at the division between mammals and non-mammalian vertebrates during the Permian-Triassic boundary (~252 Ma). Understanding Nrf2 evolution as an effective antioxidant response may have repercussions for improved human health.

The ‘Great Oxygenation Event’ (GOE), at 2.45–1.85 Ga is recognised as the most geologically critical environmental change impacting the history of life on Earth^1^. Oxygen-producing photosynthetic cyanobacteria appeared much earlier, preceding the increase of atmospheric oxygen marked by the onset of the GOE^2^, but this oxygen was removed from the atmosphere by rapid oxidation of reduced minerals, precipitating especially vast deposits of ferric oxide from the oxidation of dissolved oceanic ferrous iron. Only after this mineral oxygen sink approached saturation, a process colloquially referred to as the ‘Rusting of the Earth’, did atmospheric oxygen increase at the advent of the GOE, giving a time-lag from the origin of oxygen-producing photosynthetic cyanobacteria that seems to have lasted ~1 Ga^1^. The GOE provided biologically useable molecular oxygen necessary for aerobic respiration, a decidedly more efficient energy-generating process than pre-existing metabolic pathways, thus setting the stage for an evolutionary transition to the aerobe-dominated biota that continues to this day.

An important problem key to the success of the history of aerobic life on Earth is how cellular processes co-adapted to overcome the metabolic toxicity that results from use of highly reactive molecular oxygen. In aerobic respiration, enzyme catalysed four-electron reduction of oxygen is considered to be a relatively safe process producing water at the terminal end of the mitochondrial electron transport chain. The reductive environment of cells, however, provides ample opportunities for oxygen to undergo successive non-enzymatic univalent reduction, these processes being exacerbated by electrophilic xenobiotics and abiotic agents such as solar ultra-violet radiation. Oxidative stress is the net outcome of oxidative damage to biologically important molecules such as proteins, lipids, carbohydrates and nucleic acids caused by the generation of these reactive oxygen species (ROS). To survive in such a reactive oxygen environment, living organisms produce or sequester a variety of water- and lipid-soluble antioxidant compounds such as vitamins C and E. Oxygen metabolising organisms additionally produce an arsenal of antioxidant enzymes that inactivate ROS. Animal genomes often express over 200 antioxidant and xenobiotic detoxifying enzymes^3^. The regulated induction and expression of these genes to protect against metabolically induced oxidative stress and electrophilic toxicity is co-ordinated by a small number of related nuclear transcription factors of the bZip/CNC family of proteins, the most important of these being the master regulator, nuclear factor erythroid 2-related factor 2 (Nrf2). The Kelch-like ECH-associated protein 1 (Keap1) forms an anchor complex with Nrf2. This complex dissociates in response to ROS and toxic electrophiles, thereby releasing Nrf2 which then binds to the nuclear antioxidant response element (ARE) and co-ordinates transcription of multiple antioxidant and detoxifying enzymes^4^.

The domain architecture of Nrf2 is highly conserved across many diverse species of aerobic organisms. Our previous phylogenetic analyses clearly revealed that, whilst absent in bacteria, archaea and plants, the Keap1–Nrf2 pathway predates the fungal–metazoan divergence^5^. Here we present a ‘molecular clock’ which estimates that the evolutionary origins of Nrf2 is allied to the timing of the global transition from anaerobic to aerobic conditions, and provides first demonstration of a metabolic adaptation in multiple eukaryotic ancestors having evolved a significant molecular response to the GOE.

## Results and Discussion

**Figure 1:**
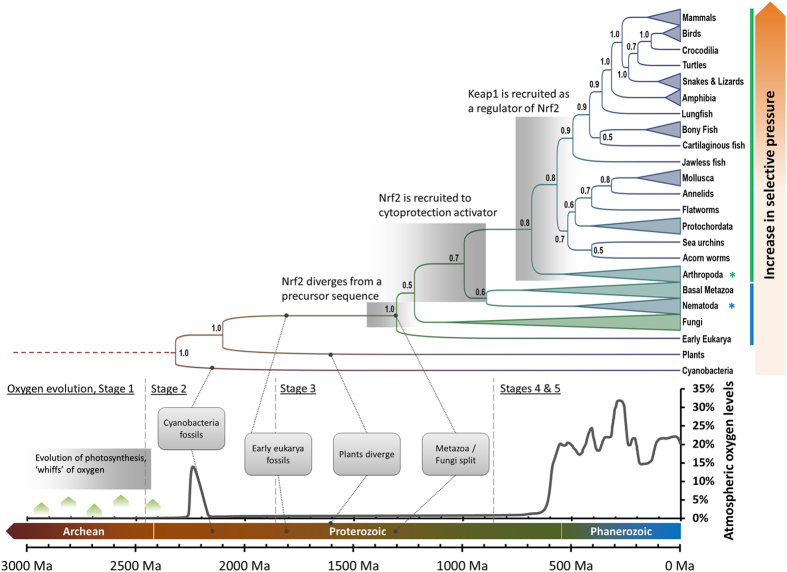
displays the Nrf2 phylogenetic tree relative to atmospheric oxygen levels during the latter period of Earth’s history. The chart presents the traditional “5-stage model” of oxygen evolution constructed from compiled data^1^,^8^,^9^,^10^,^11^,^12^,^13^,^14^,^15^, with the trend line representing a “best guess” model; Stage 1 represents a period when the atmosphere and oceans were largely anoxic; Stage 2 commences the ‘Great Oxygenation Event’; Stage 3 is the period during which atmospheric oxygen levels remained low due to continued absorption by the oceans and oxidative weathering of the terrestrial crust; Stage 4 is the period after saturation of global oxygen buffers, during which oxygen levels rise towards present (Stage 5) atmospheric levels (PAL). The Earth timeline and major geological periods^43^ are compiled and coloured by age. Eukarya and cyanobacteria appearances are noted according to first confirmed fossil evidence^17^. Proposed time frames are shown for major Nrf2 divergence and recruitment events. Taxa known or predicted to contain the Keap1-Nrf2 signalling pathway are denoted by the vertical green bar, while taxa containing Nrf2 only (without Keap1) are denoted in blue. Invertebrates with an experimentally validated Nrf2 system are marked with a star (*). Evolutionary pressure increases towards more recently evolved phyla as schematically shown by an increasing orange hue in the selective pressure bar (decrease in dN-dS test statistic and decrease in p-value for null hypothesis of neutral evolution).

In order to reconstruct the evolutionary life history of Nrf2 in response to mounting oxidative stress, a Bayesian phylogenetic analysis of Nrf2 sequences retrieved from the genome sequences of many diverse taxa was performed together with a prediction of evolutionary pressure as evinced by a calculation of synonymous to non-synonymous nucleotide base substitution rates. The results are presented as a phylogenetic tree which was converted to a ‘molecular clock’ using widely accepted paleontological estimates for known splits between major animal phyla. The molecular clock was calibrated based on best paleontological estimates for the divergence of major phyla using compiled data from previous studies, reflecting the very recent hypothesis of Hedges *et al*. (2015) that speciation is independent of adaptation^6^,^7^. The resulting phylogenetic reconstruction was mapped against the changing level of atmospheric oxygen over geological time–with the Phanerozoic oxygen levels taken as a composite of the data afforded from the GEOCARBSULF model of Berner^8^,^9^,^10^, the glaciation-linked oxygen rise models of Harada *et al*.^11^ and a compilation of other data^1^,^12^,^13^,^14^,^15^. It should be noted that the oxygen curve presented in Fig. 1 is based on “best estimates” and should thus be considered semi-quantitative. While Phanerozoic oxygen trends are well established^8^,^9^,^10^, with moderate error margin^9^, there is still a level of uncertainty over Proterozoic oxygen estimates. Specifically, the estimated date of origin of photosynthesis ranges from 2,400 to 3,000 Ma^15^,^16^,^17^ and the exact oxygen levels over the majority of the Proterozoic era are subject to controversy^18^,^19^,^20^, as are oxygen level dynamics during the Ediacaran era^11^,^14^,^21^. Thus, while future research might lead to fine tuning of the oxygen level data, the pattern of change presented in Fig. 1 is considered reliable as regards the *major* trends in oxygen change over geological time.

The results presented in Fig. 1 allow inference of Nrf2 emergence and sequence diversification as speciation occurred and oxidative stress increased due to changes in atmospheric oxygen. These data would strongly suggest, therefore, that Nrf2 first appeared having evolved from an early eukaryotic peptide that contained a bZIP/CNC domain sequence in Stage 3 of atmospheric oxygenation during the mid-Proterozoic when oxygen was released into the atmosphere but was rapidly absorbed into the Earth’s ocean sediments and terrestrial crust^13^. The divergence of cyanobacterial Nrf2-like sequences, which we use as an out-group in our evolutionary tree (Fig. 1), differ in evolutionary time from the expected eukaryote-plant divergence (1500 Ma)^6^, placing plant Nrf2-like sequences closer to cyanobacterial sequences rather than those of early eukaryotes. This significant difference in the bZip/CNC domain architecture of plants is consistent with a lack of nuclear Nrf2-like activation in the response of plants to oxidative stress^22^ and absence of detectable homology to the Keap1-Nrf2 pathway in plant genomes^5^. Perhaps Nrf2-like sequences in plants might be explained by horizontal transfer during early endosymbiosis, assuming such sequences were inherited from a cyanobacterial precursor of the plant chloroplast^23^. Interestingly, the predicted oxygen level spike during Stage 2 of atmospheric oxygenation during the mid-Paleoproterozoic period does not seem associated with Nrf2 evolution (as evidenced by the lack of Nrf2 homology in cyanobacteria and plants^5^) and instead points to an Nrf2-like mechanism as a Metazoan adaptation.

**Figure 2:**
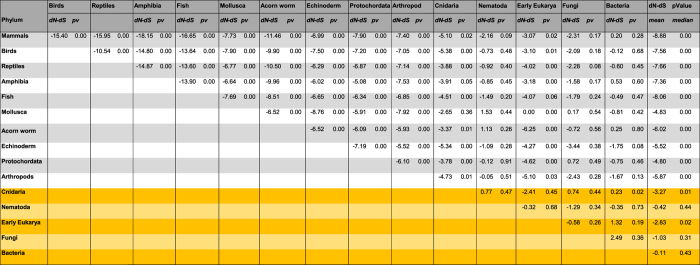
Displays the Codon-based Z test of selection matrix performed on 42 DNA sequences of Nrf2 homologs from major eukaryotic phyla with cyanobacterial sequence used as outgroup (plant outgroup sequences could not be aligned with the dataset). Analyses were conducted using the Nei-Gojobori method, and results are grouped by major eukaryotic phyla with the phylum dN-dS value calculated as the mean of group members. All positions with less than 95% site coverage were eliminated. There were a total of 352 positions in the final dataset and fewer than 5% alignment gaps, missing data, and ambiguous bases were allowed at any position. Evolutionary analyses were conducted in MEGA6. Figure 2 shows dN-dS and the p-value for null hypothesis of strict neutrality (dN = dS) for each pair of phyla. Phyla likely to have lower selective pressure compared to vertebrates (median P-value >10^−3^) are highlighted in yellow. Phyla without selective pressure (based on a p-value of 0.05 as the significance threshold) are highlighted in orange. Codon-based Z test of selection matrix.

Evolutionary pressure determined by the Codon-based Z-test of selection on both nucleotide and amino acid sequences^24^ (Fig. 2) reveals strong purifying selection of Nrf2 sequences for all bilateral animals (all p-values ≤ 10^−3^) with the exception of nematode worms. Cnidaria and other basal metazoans display limited evidence for negative selection (p-values of all tests fall between 0.01 and 0.10). Nematodes and non-metazoan Nrf2 sequences exhibit no significant evidence for selective pressure (p-values of tests are >0.10, as displayed in Fig. 2). Regulation of the Nrf2 antioxidant response exists in simple invertebrates as demonstrated empirically for *Caenorhabditis elegans.* The Nrf2 homolog SKN-1 in *C. elegans*, although serving a similar function, has significant differences in structure and regulatory pathways^25^,^26^, lacking also a regulatory Keap1 interaction that is present in *Drosophila melanogaster*^27^. Notably, the SKN-1 sequence of *C. elegans* is closer to the homolog sequences of basal metazoans such as cnidarians, indicating that recruitment occurred prior to the metazoan radiation of the Cambrian Explosion. This time frame matches the transition from Stage 3 and the start of Stage 4 of atmosphere oxygenation during which oxygen absorbing buffers in the Earth’s oceans and crust were reaching saturation and atmospheric oxygen levels began to rise^1^. This rise in atmospheric and ocean oxygen levels led to an increase in aerobic metabolic stress causing evolutionary pressure towards the expansion of antioxidant response systems in animals. Tests for selective pressure indicate that Nrf2 sequences of basal metazoans were under limiting selective pressure, with averaged p-values for evolution neutrality >1 (testing *H*_0_: dN = dS, Fig. 2). According to empirical evidence gained from *Drosophila melanogaster*^27^,^28^, genomic Keap1 recruitment occurred in early invertebrates preceding the divergence of the class Insecta after the Cambrian Explosion. This time frame coincides with rising levels of atmospheric O_2_ during Stage 4 of the Earth’s oxygenation and matches the increased evolutionary pressure (measured by Codon-based Z-test of selection, Fig. 2) detected in Nrf2 sequences from taxa of the early Bilateria. Together, these lines of evidence suggest that rising levels of oxygen led to recruitment of Keap1 for enhanced regulation of Nrf2 for the transcription of cytoprotective genes in the response of animals to oxidative stress.

It is unclear whether fungi and early diverging metazoans possessed a one-protein (Nrf2 only) or two protein (Keap1-Nrf2) antioxidant response system. Nematodes having only a Nrf2-like sequence^26^,^29^ are grouped with basal metazoans suggesting a one-protein system that likely evolved early in metazoan development. The position of nematode Nrf2-like sequences, however, differs from what would be expected from the generally accepted Tree of Life in which nematodes belong to a clade of ‘moulting animals’ along with arthropods and several smaller phyla^30^. The nematode Nrf2-like sequence SKN-1 also has a lower selective pressure (as measured by Codon-based Z-test of selection, Fig. 2) than the Nrf2 of most animals. Such, an alternative explanation is that recruitment of Keap1 had occurred shortly after Nrf2 evolution into an antioxidant response regulatory system at a time close to the animal-fungal divergence at late Stage 3 of atmospheric oxygenation. Homologous Keap1 proteins of nematodes may have subsequently lost persistence of regulatory control over Nrf2-like function in nematodes, perhaps due to a lack of environmental selective pressure attributed to the often hypoxic soil-dwelling lifestyle of worms. In contrast, tissues of cnidarians harbouring phototrophic endosymbionts can tolerate extremes of oxygen saturation^31^, thus demanding efficient means to control oxidative damage. Accordingly, additional elaboration of Nrf2 activity in fungi and basal metazoans is essential to better elucidate evolutionary processes, which is enabled by the recent availability of several cnidarian genome annotations, including that of the scleractinian coral, *Acropora digitifera*^32^.

In summary, we demonstrate that orthologues of Nrf2 first appeared in fungi around 1.5 Ga^5^ during the Paleoproterozoic when photosynthetic oxygen was being absorbed into the oceans culminating in prolonged low oxidative stress^1^. A subsequent significant divergence in Nrf2 is seen to occur during the split between fungi and the Metazoa approximately 1.0–1.2 Ga^33^; at a time when oceanic ventilation released free oxygen to the atmosphere, but with most of this being absorbed by methane oxidation and oxidative weathering of land surfaces until approximately 800 Ma^12^,^18^. Atmospheric oxygen levels thereafter accumulated during the Neoproterozoic giving rise to metazoan success during the Ediacaran period (635–541 Ma) leading to the Cambrian explosion (radiation) commencing at ~541 Ma^34^. Atmospheric O_2_ levels then rose in the late Paleozoic (359–252 Ma), driving further Nrf2 sequence divergence and Keap1 recruitment for Keap1-Nrf2 regulation of the oxidative stress response at the division between mammals and non-mammalian vertebrates during the during the Late Triassic (~225 Ma)^35^,^36^. Understanding the evolution of Nrf2 and recruitment of other protein partners into an effective antioxidant response cascade might provide novel insights into the human aging process since oxidative stress is believed to be one of the key factors in aging. This could, in turn, reveal possible new intervention strategies to improve metabolic health in our worldwide ageing population.

## Methods

The Nrf2 phylogenetic tree was constructed using BEAST version 2.3.0^37^ using a selection of Nrf2 homologs sourced from major metazoan and fungal phyla, and basic leucine zipper transcription factors from plant and cyanobacteria are utilised as outgroups. Sequences were aligned using T-Coffee Expresso^38^ and T-Coffee Psi-Coffee^38^ aligners and were evaluated using the T-Coffee TCS method to verify multiple alignment transitional consistencies^39^. The phylogenetic tree was calibrated based on best paleontological estimates for the emergence of Eukaryota, the metazoan-fungal split and a set of animal phyla divides using compiled data from previous studies^6^,^7^,^33^,^40^, In order to assess the robustness of phylogenetic reconstruction and selective pressures in the evolution of Nrf2 based on increasing oxidative stress, data were split into subgroups (Mammals, Amniotes, Tetrapods, Vertebrates, Deuterostomia, Bilateria, Eumetazoa and early Eukarya datasets) to examine protein and DNA sequence divergence using MEGA 6^41^. For each group, sequences were aligned using ClustalW^42^, and alignments were analysed using the HyPhy test of codon selection and a codon-based Z test of selection for DNA sequences^24^. Accordingly, Maximum Likelihood and Neighbor Joining Trees were constructed for each group, and tree topologies were compared to verify consistency of results. Tests confirmed the robustness of taxonomical grouping and codon-based selection tests within and between animal subgroups (data not shown). Multiple alignments with Nrf2-like DNA plant sequences were not of sufficient quality to perform Codon-based tests of selection. Detailed bioinformatics methodology is provided in Supplementary Data File 1.

## Additional Information

**How to cite this article**: Gacesa, R. *et al*. Rising levels of atmospheric oxygen and evolution of Nrf2. *Sci. Rep.* **6**, 27740; doi: 10.1038/srep27740 (2016).

## Supplementary Material

Supplementary Information
