# Supplementary material for: Rising levels of atmospheric oxygen and evolution of Nrf2
Source: Sci Rep. 2016 Jun 14;6:27740. doi: 10.1038/srep27740 (PMC4906274; doi:10.1038/srep27740)
Supplement: Supplementary Information [file srep27740-s1.doc]

**Rising levels of atmospheric oxygen and evolution of Nrf2**

Ranko Gacesa, Walter C. Dunlap, David J. Barlow, Roman A. Laskowski and

Paul F. Long

**Supplementary Data File 1: Bioinformatics methodology**

**1.0. Selection of sequences for phylogenetic reconstruction**

Translated genomes of metazoan and fungi deposited in UNIPROT and NCBI Refseq databases as of 01/06/2015 were data mined for homologs to human Nrf2 using HMMER^1^ (HMM profiles generated for Nrf2 and neh1 – neh7 conserved sequences of Nrf2 using vertebrate Nrf2 sequences), psi-BLAST^2^ and a previously developed Distant Homology Search Pipeline (DHSP^3^). If more than one homolog could be identified in a given genome, all potential homologs were investigated for Keap1 binding motifs DLG and ETGE and beta-TRCP binding motif DSGIS using pattern matching, with one mismatch and putative homolog selected based on the presence of DLG / ETGE motifs and HMMER e-values for neh motifs. In the case of ambiguous results, pairwise BLAST alignment with human, mouse and *Drosophila* Nrf2 sequences were used to select putative homologs. DNA sequences were selected as coding DNA for Nrf2 protein homologs if available, and by BLAST searches against NCBI nucleotide databases if putative Nrf2 homologs lacked annotated coding sequences.

**1.1. Reconstruction of dated phylogenetic tree**

A dated phylogenetic tree was constructed using the BEAUTI/BEAST 2.3.0 framework^4^, using the following 63 protein sequences from a set of major metazoan phyla. Plant and bacterial sequences were used as out-groups (see 2.4 for list of sequences). Sequences were aligned using T-Coffee^5^, M-Coffee^6^, T-Coffee Expresso^7^, Psi-Coffee^7^, ClustalW^8^, MUSCLE^9^ and MAFFT^10^ multiple alignment tools, with two independent runs for each tool. Each alignment was evaluated using T-Coffee TCS^11^ for transitional consistency. Based on TCS scores, Expresso and Psi-Coffee were chosen as aligners of choice and three independent alignments were generated by each of these methods. Phylogenetic trees were constructed for each multiple alignment, using the following BEAST parameters:

- JTT evolutionary model^12^, with Gamma site rates (Substitution rate, Proportion of invariant sites and Shape estimated during simulation, 4 gamma categories)
- Relaxed exponential clock model, with estimated rates and continuous rate variations along the tree
- Simulation was run for 100 000 000 MCMC generations

Following date ranges were used for calibration points^13–15^:

- Bacteria-Eukarya divergence: ≈ 2200-4200 Ma (uniform prior probability; min 2200, max 4200 Ma; constrained as monophyletic outgroup)
- Bird-Reptile split: ≈ 255-300 Ma (gamma distributed prior probability; alpha 1.25, beta 10.0, offset 255.0 Ma)
- Eumetazoa – metazoan divergence: ≈ 550-950 Ma (gamma distributed prior probability; alpha 1.25, beta 85.0, offset 550.0 Ma)
- Fungi – Animal divergence: ≈ 900-1500 Ma (normally distributed probability; mean 1200, sigma 100 Ma)
- Human – Chimpanzee split: ≈ 6 – 7 Ma (gamma distributed prior probability; alpha 1, beta 0.2, offset 6.0 Ma)
- Human – Mouse split: ≈ 69 – 110 Ma (gamma distributed prior probability; alpha 1.25, beta 8.0, offset 69.0 Ma)
- Plant – Animal split: ≈ 800 – 2000 Ma (normally distributed probability; mean 1400, sigma 200 Ma)
- Vertebrates – Invertebrates split: ≈ 500 – 600 Ma (gamma distributed prior probability; alpha 2, beta 15.0, offset 500.0 Ma)

Final trees were generated using *treeannotator (BEAST 2.3.0 package)* with *burnin value 0.25*, with other parameters left at default values. Trees were manually compared for consistency. The tree presented in the main article was generated using the *Figtree* tool with species from the same phylum collapsed for clarity, and posterior probabilities calculated as the mean between all BEAST runs. Comparison of trees found that all splits were highly consistent, even within clades with low posterior probability support.

**1.2. Selective pressure analysis**

Evolutionary selective pressure analysis was conducted using HyPhy test of codon selection and a codon-based Z test of selection^16^ for DNA sequences by tools integrated into MEGA 6.0 toolkit^17^. DNA sequences used for these tests are listed in 2.5.

**1.3. Data robustness analysis**

In order to confirm the robustness of the data, DNA and protein sequences (2.4. and 2.5.) were divided into the following subgroups:

- Mammals
- Reptiles and Birds
- Land dwelling vertebrates
- All vertebrates
- Bilaterian animals
- Metazoa

And each group was further analysed using MEGA 6.0 by following protocol:

1. Sequences in the group were aligned using ClustalW and MUSCLE (using default parameters)
2. Maximum likelihood models were analysed using the MEGA Maximum likelihood (ML) model selection tool (model with lowest BIC and AICc scores were picked as models for choice)
3. Phylogenetic trees were reconstructed for each alignment using Neighbor joining and Maximum likelihood methods, using total deletion method and partial deletion method with cutoff of 95 % position coverage.
4. HyPhy test of codon selection and a codon-based Z test of selection were performed on the group.

In addition, multiple alignments used for dated tree reconstruction were also analyzed using MrBayes, version 3.2^18^, using the following parameters for reconstruction of an undated phylogenetic tree:

- Prior for amino acid model set to mixed (aamodelpr=mixed), with gamma model invariant sites
- 10 000 000 MCMC generations, with 8 parallel chains and 4 runs
- Other parameters left at default values

Results of all tests were compared, tree topologies and dN-dS values were found to have high consistency between and within groups, with ClustalW alignments and partial deletion methods generating results with high agreement to BEAST and MrBeast reconstructions. MUSCLE alignments and total deletion methods generated lower bootstrap values.

**1.4 Sequences for dated phylogeny reconstruction.**

>ma_h_sapiens gi|693842|gb|AAB32188.1| Nrf2 [Homo sapiens]

MDLIDILWRQDIDLGVSREVFDFSQRRKEYELEKQKKLEKERQEQLQKEQEKAFFTQLQLDEETGEFLPI

QPAQHTQSETSGSANYSQVAHIPKSDALYFDDCMQLLAQTFPFVDDNEVSSATFQSLVPDIPGHIESPVF

IATNQAQSPETSVAQVAPVDLDGMQQDIEQVWEELLSIPELQCLNIENDKLVETTMVPSPEAKLTEVDNY

HFYSSIPSMEKEVGNCSPHFLNAFEDSFSSILSTEDPNQLTVNSLNSDATVNTDFGDEFYSAFIAEPSIS

NSMPSPATLSHSLSELLNGPIDVSDLSLCKAFNQNHPESTAEFNDSDSGISLNTSPSVASPEHSVESSSY

GDTLLGLSDSEVEELDSAPGSVKQNGPKTPVHSSGDMVQPLSPSQGQSTHVHDAQCENTPEKELPVSPGH

RKTPFTKDKHSSRLEAHLTRDELRAKALHIPFPVEKIINLPVVDFNEMMSKEQFNEAQLALIRDIRRRGK

NKVAAQNCRKRKLENIVELEQDLDHLKDEKEKLLKEKGENDKSLHLLKKQLSTLYLEVFSMLRDEDGKPY

SPSEYSLQQTRDGNVFLVPKSKKPDVKKN

>ma_m_musculus gi|6754832|ref|NP_035032.1| nuclear factor erythroid 2-related factor 2 [Mus musculus]

MMDLELPPPGLQSQQDMDLIDILWRQDIDLGVSREVFDFSQRQKDYELEKQKKLEKERQEQLQKEQEKAF

FAQFQLDEETGEFLPIQPAQHIQTDTSGSASYSQVAHIPKQDALYFEDCMQLLAETFPFVDDHESLALDI

PSHAESSVFTAPHQAQSLNSSLEAAMTDLSSIEQDMEQVWQELFSIPELQCLNTENKQLADTTAVPSPEA

TLTEMDSNYHFYSSISSLEKEVGNCGPHFLHGFEDSFSSILSTDDASQLTSLDSNPTLNTDFGDEFYSAF

IAEPSDGGSMPSSAAISQSLSELLDGTIEGCDLSLCKAFNPKHAEGTMEFNDSDSGISLNTSPSRASPEH

SVESSIYGDPPPGFSDSEMEELDSAPGSVKQNGPKAQPAHSPGDTVQPLSPAQGHSAPMRESQCENTTKK

EVPVSPGHQKAPFTKDKHSSRLEAHLTRDELRAKALHIPFPVEKIINLPVDDFNEMMSKEQFNEAQLALI

RDIRRRGKNKVAAQNCRKRKLENIVELEQDLGHLKDEREKLLREKGENDRNLHLLKRRLSTLYLEVFSML

RDEDGKPYSPSEYSLQQTRDGNVFLVPKSKKPDTKKN

>ma_p_troglodytes gi|332814816|ref|XP_001145876.2| PREDICTED: nuclear factor erythroid 2-related factor 2 isoform X1 [Pan troglodytes]

MMDLELPSPGLPSQQDMDLIDILWRQDIDLGVSREVFDFSQRRKEYELEKQKKLEKERQEQLQKEQEKAF

FAQLQLDEETGEFLPIQPAQHIQSETSGSANYSQVAHIPKSDALYFDDCMQLLAQTFPFVDDNEVSSATF

QSLVPDIPGHIESPVFIATNQAQSPETSVAQVAPVDLDGMQQDIEQVWEELLSIPELQCLNIENDKLVET

TMVPSPEAKLTEVDNYHFYSSIPSMEKEVGNCSPHFLNAFEDSFSSILSTEDPNQLTVNSLNSDATVNTD

FGDEFYSAFIAEPSISNSMPSPATLSHSLSELLNGPIDVSDLSLCKAFNQNHPESTAEFNDSDSGISLNT

SPSVASPEHSVESSSYGDTLLGLSDSEVEELDSAPGSVKQNGPKTPVHSSGDMVQPLSPSQGQSTHVHDA

QCENTPEKELPVSPGHRKTPFTKDKHSSRLEAHLTRDELRAKALHIPFPVEKIINLPVVDFNEMMSKEQF

NEAQLALIRDIRRRGKNKVAAQNCRKRKLENIVELEQDLDHLKDEKEKLLKEKGENDKSLHLLKKQLSTL

YLEVFSMLRDEDGKPYSPSEYSLQQTRDGNVFLVPKSKKPDVKKN

>ma_o_anatinus gi|620978732|ref|XP_007669464.1| PREDICTED: nuclear factor erythroid 2-related factor 2 isoform X1 [Ornithorhynchus anatinus]

MLILSGWCHLSPTPRAKDMNLIDILWRQDIDLGAGREVFDFCQRQKEYELEKQKKLEKERQEQLQKEREQ

ALLAQFQLDEETGEFLPIQPARPSQLEGGDGPAAFSQSPPTPKPDALTFDDCMQLLTETFPFVDDNEVAP

ATLQSLSPPPAESSPVFVPPSPTPAPAEAPVLEPAATDSAAMQDIEQVWEELLSIPELQCLNIQNDKQAE

AAPLPSPEPKSGAADRPYGFYDVLSPLACTIEKEMSDSSPAFLGAFEGSALPTQDLSVSGACAQPPSPSL

GPDFCEDFYTTFVVELEPGAEGAGAPSRLLTDLLNEPVDLADLALCKAFATPRPCGRPESNDADSGISLN

TSPAAASPEPLADSVDGDAAPGSSDSETDDVDSGPPGGAKIRPHAGAEGGRQADPPKKEVLAGRGPPPGT

RDRPAGRLEAHFTRDEQRAKALQIPFPVEKIINLPVDDFNEMMSKEQFSEAQLALIRDIRRRGKNKVAAQ

NCRKRKLENIVELEQDLDHLKDEKEKLLKEKGEHDLSLRLLKQQLSSLYLEVFSMLRDQDGQPYSPADYS

LQQTRDGHVFLVPKSKKPGGQHGN

>ma_e_edwardii gi|585637261|ref|XP_006878862.1| PREDICTED: nuclear factor erythroid 2-related factor 2 isoform X1 [Elephantulus edwardii]

MMDLELPSPELPSQQDMDLIDILWRQDIDLGVSREVFDFSQRRKEYELEKQKKLEKERQE

QLQKEQEKAFFAQLQLDEETGEFLPIQPAQHIQSETSGSADYSQGAHIPKPDALYFDDCM

QLLAETFPFVDNNEVSSATFQSLVPDISSHIESPVFIAPSQTQTPETPVLQTTPEHLDNM

MQDVDQVWEELLSIPELQCLNIQNDKLVETNTVASTETKLTEIDNSYHFYSSIPSLEKEV

GDCSSNFLNAFEDFFDNILPTDDSNQLTVNSLNANATINTDFGDEFYSAFIAEPSVSNSI

SSSAALSQPLTELLNGSIDISDLSLCKAFNQSHPESTAEFNDSDSGISLNTSPSMASPEH

SVESSIYGDTPLGFSDSEMEERDSTPESVKLNGPKTQPVQSSEDTAQPLSPSPGHSASGG

DALCENTPKNELPVSPGHRKTPFTKDKHSSRLESHLTRDELRAKALHIPFPVEKIINLPV

DDFNEMMSKEQFSEAQVALIRDIRRRGKNKVAAQNCRKRKLENIVELEQDLDHLKDEKEK

LLREKGENDKSLHLLKKQLSTLYLEVFSMLRDEDGKPYSPSEYSLQQTRDGNVFLVPKSK

KPDVKKN

>ma_f_catus gi|410968910|ref|XP_003990942.1| PREDICTED: nuclear factor erythroid 2-related factor 2 isoform X1 [Felis catus]

MMDLELPPPGLPSQQDMDLIDILWRQDIDLGVSREVFDFSQRRKEHELEKQKKLEKERQEQLQKEQEKAF

FAQLQLDEETGEFLPIQPAQHIPSETSGSANYSQVAHIPKPDALYFDDCMQLLAETFPFVDDNEVSSAAF

QSLVPDIPSQIENPVFIAPNQAQSPQTLVTQSVIADLDNMQQDIEQVWEELLSIPELQCLNIQNDKLVET

STVPSPETKMTEIDNNYHFYSSMPSLEKEVGNCSPHFLSAFEDSFSSILSTEDSSQLTVNSLNSDATINT

DFGDEFYSAFIAEPSSSNSMPSSATLSQSLSELLNGPIDVSDLSLCKAFNQNHPESTEFNDSDSGISLNT

SPGLASPEHSVESSVYGDTPLGFSDSEMEEIDSAPGSVKQNGPKTQPVQSSGDTVQPLSPSPGHSAPVCD

AQCENTPKKELPVSPGHRKTPFTKDKHSSRLEAHLTRDELRAKALHIPFPVEKIINLPVDDFNEMMSKEQ

FNEAQLALIRDIRRRGKNKVAAQNCRKRKLENIVELEQDLDHLKDEKEKLLREKGENDKSLHLLKKQLST

LYLEVFSMLRDEDGKPYSPSEYSLQQTRDGNVFLVPKSKKPDVKKN

>ma_m_brandtii gi|554525642|ref|XP_005857673.1| PREDICTED: nuclear factor erythroid 2-related factor 2 isoform X1 [Myotis brandtii]

MREREIIETDDGPGNQRDQDMDLIDILWRQDIDLGVSREVFDFSQRRKEHELEKQKKLEKERQEQLEKEQ

EKAFFAQLQLDEETGEFLPIQPAQHIPSETSGSANYSQVAHIPKPDALYFDNCMQLLAETFPFVEDNEVS

SPTFQSLVPDVPSHIESPVFTAPSQTQSSEPVVLQLISDLGNMQQDIEQVWEELLSIPELQCLNIQNDKL

VETNTVPSPETKQADIDNSYHFYSSIPTLEKEVGNCSPPFLNAFEDSFSSILTTEDPSQLTVNSLNSNAT

INTDFGDEFYSAFVEEPSINNSMSSSATFSQSLSELLYGPIDVSDLSLCKAFNPESTAEFNDSDSGISPN

TSPSMASPEHSVESSGYGDTPLGFSDSEMEETDSAAGSVKHSGPKTQPVQTSGETVHPPSPSRGHSAPVS

DAQCENTQKKELPVSPGHRKTPFTKDKHSSRLEAHLTRDELRAKALHIPFPVEKIINLPVDDFNEMMSKE

QFNEAQLALIRDIRRRGKNKVAAQNCRKRKLENIVELEQDLDHLKDEKEKLLKEKGENDRNLHLLKKQLS

TLYLEVFSMLRDEDGKPYSPSEYSLQQTRDGNVFLVPKSKRPDVKK

>ma_m_domestica gi|612019826|ref|XP_001377155.2| PREDICTED: nuclear factor erythroid 2-related factor 2 isoform X1 [Monodelphis domestica]

MLNFVLPRDMNLIDILWRQDIDLGARREVFDFSQRRKEHELEKQKKLEKERQEQLQKEQEKAFLAQLQLD

EETGEFLPIQPAQHIEPSTSASYSQAADIPKADALFFDDCMQLLAETFPFVEDNEVSSATFQSLVPDHID

SNPVFITSSQAQLPESSVLQSIVENNMQDIEQVWEELLSIPELQCLNIENDKLAEATIVPSPEAKPTEIN

DSYNFYTSLSTMEKEVATCNPDFLSAFEDSFGNILPTEDPNQLRMNSLNSNATINTDFCEEFYSTFIAET

NINNSMPSPAHISQSLSELLNEPIDISDLSLCKAFNSNPPENPPECNDSDSGISLNTSSNMASPEHSVES

SLYGDTPLGFSDSEMEDVDSAPGSTQQSGARMQPVPFQEDMPYPVSPTQGPTVPAPDALQSVSTPKRESP

TSPGHQKVPFTKDKHSGRLESHFTRDEMRAKALHIPFPVEKIINLPVDDFNEMMSKEQFNEAQLALIRDI

RRRGKNKVAAQNCRKRKLENIVELEQDLDHLKDEKEKLLRERGENDKSLHLLKKQLSTLYLEVFSMLRDE

NGEPYSPSEYSLQQTRDGNVFLVPKSKKPDIKRN

>ma_o_afer gi|634820840|ref|XP_007936500.1| PREDICTED: nuclear factor erythroid 2-related factor 2 isoform X1 [Orycteropus afer afer]

MMDLELPSPGLPSQQEMDLIDILWRQDIDLGVSREVFDFSQRRKEYELEKQKKLEKERQE

QLQKEQEKAFFAQLQLDEETGEILPIQPAQHIHSETSGSANYSQVAHIPKLDVLYFTDCM

QLLAETFPFVEDNEVSSATFQSLVPDIPSHIEPPIFIAPDQSPETPVLQTTVAHLDNMQD

VDQVWEELLSIPELQCLNIQNDKLVETSTVPSPETKLTEIDNYHFYPSIPSLEKEVGDCS

PHLLNAFEDFFGSILPTDDPGQLTVNSLNSNTINTDFGDEFYSAFIAEPSINNSMSSSAT

LSQPLSELLNGPIDVSDLSLCKAFNENHPESTAEFNDSDSGISLNTSPSRASPEHSVESS

IYGDTPLGFSDSEMEERDSTPESVQQNGPKTQPVQSSGDIVQPLSPSPGHSASVHDAQCE

NAPQKELPVSPGHRKTPFTKDKHSNRLEAHLTRDELKAKALRIPFPVEKIINLPVDDFNE

MMSKEQFNEAQVALIRDIRRRGKNKVAAQNCRKRKLENIVELEQDLDHLKDEKEKLLKEK

GENDKSLHLLKKQLSTLYLEVFSMLRDEAGQPYSPSEYSLQQTRDGNVFLVPKSKKPDVK

KN

>ma_o_cuniculus gi|655828110|ref|XP_002712351.2| PREDICTED: nuclear factor erythroid 2-related factor 2 isoform X1 [Oryctolagus cuniculus]

MMDLELPPSGLQSQQDMDLIDILWRQDIDLGVSRDVFDFSQRQKEYELEKQKQLEKERQEQLQKEQEKAF

FAQLQLDEETGEILPIQPAQHIQSETSGSANYSQVAHIPKPDALYFDDCMQLLAETFPFVDDNEVSSATF

QSLVPDTPSHVESPVFTAPNQAQTPETSFVQVAVADLNNMEQNIEQIWEELLSIPELQCLNIEKDKLVET

TTVPSAEVKLTEVDNNYHFYSSAPSLEKEDNCSAHFLSAFEDSFGSILSADDPAQLSVNSNATLNTDFGD

EFYAAFIAEPSVSNSMSSAPISQSLSELLNGPIDVSDLSLCKAFNQNHPESTEFADSDSGISLNTSPSMA

SPEHSVESSVCGDTPLGFSDSEMEELDSTHGVVKQNASKTQPIHSSGDTVQPLSPSGGYSAPVHNAQCEN

TPKKETPGSPSPRKTPFTKDKHSGRLEAHLTRDELRAKALHIPFPVEKIINLPVDDFNEMMSKEQFNEAQ

LALIRDIRRRGKNKVAAQNCRKRKLENIVELEQDLDHLKDEKEKLLKEKGENDKSLHLLKKQLSTLYLEV

FSMLRDEHGKPYSPSEYSLQQTRDGNVFLVPKSKKPDVKH

>av_a_platyrhynchos gi|514719877|ref|XP_005013612.1| PREDICTED: nuclear factor erythroid 2-related factor 2 [Anas platyrhynchos]

MQLSWVRFPGASIGEHGNFRGRGHGVKDMNLIDILWRQDIDLGARREVFDFSQRQKEYELEKQKKLEKER

EEQLQKEQEKALLAQLELDEETGEFVPVQPAQRIQSENTEPPITFSQSTHTSKPEAEALSFDDCMQLLAE

AFPFIDDNEASSAAFQSMVPAQIDSDPEFISSNQTQPPESPGIVPLTDAENMQNIEQVWEELLSLPELQC

LNIENDNLAEVSTITSPETKSTEMHNGYNYYNSLPIMRKDVNCGPDFLETVESPFPSILQTEDSSQLVVN

SLNNTSTSNPDFCEDFYTTFLYSKGDSDVATTNTISQSLAEILSEPIDLSDFSLWRAFNDEHSGTVPECN

DSDSGISLNANSRVASPEHSVESSACGDKTFGCSDSEMEDVDSSPGSVPQSNASVYPLQFQDQVLSSVEP

STRPPSLQCTNTPKKDPPAGPGHPKAPFTKDKPSGRLEAHLTRDEQRAKALQIPFPVEKIINLPVDDFNE

MMSKEQFSEAQLALIRDIRRRGKNKVAAQNCRKRKLENIVELEQDLSNLKDEREKLLKEKGENDKSLRQM

KKQLTTLYLEVFSMLRDEDGKSYSPSEYSLQQTRDGNVFLVPKSKKSETKL

>av_f_peregrinus gi|909796685|ref|XP_013154279.1| PREDICTED: nuclear factor erythroid 2-related factor 2 [Falco peregrinus]

MNLIDILWRQDIDLGVRREVFDFSQRQKEYELEKQKKLEKERQEQLQKEQEKALLAQLELDEETGEFVPV

QPAQRIHSENTEPPIDFSQSTQTSKPEAETLSFDDCMQLLAEAFPFIDDDEVRMLNAVVSRVCSSPEFIS

SDHAQPPESPGLVSLTDAENMQNIEQVWEELLSLPELQCLNIENDNLAEVSTITSPETKPTEMHNRYNYC

SSLPIMRKDVNCSPDFLDSMEDPFSSILPPEDTSQLSVNSLKDTSPSNSDFCEDFYATFIDTKANGDTAT

TNTISQSLAEILSEPIDLSDFSLCKAFNGNHSGTVPECNDSDSGISLNASSSVASPEHSVESSAYGDKAF

GCSDSEMEDMDSAPGNVPQSHASAYSLQLQDQVFSSMGPSARTPSLQCTNAPKEEPPAGPGHPKAPFTKD

KPSGRLEAHLTRDEQRAKALQIPFPVEKIINLPVDDFNEMMSKEQFNEAQLALIRDIRRRGKNKVAAQNC

RKRKLENIVELEQDLSNLKDEKEKLLKEKGEHDKSLRQMKKQLTTLYLEVFSMLRDEDGKSYSPSEYSLQ

QTRDGNVFLVSKSTKSETKL

>av_a_chloris gi|677997964|ref|XP_009077031.1| PREDICTED: nuclear factor erythroid 2-related factor 2 [Acanthisitta chloris]

MNLIDILWRQDIDLGARREVFDFSQRQKEYELEKQKKLEKERQEQLQKEQEKALLAQLEL

DEETGEFVPVQPAQSSQSENTEPPVVFSQTTEPSKPEAEALSFEDCMQLLAEAFPFVDEN

EVSSDAFQSLVPAQINSNSAFVSSDQSQPPDLVPPTETENMQNIEQVWEELLSLPELQCL

NIENDNLAEVSTITSPEAKPTEMHNRYNYCSSLPTMKKDVNCSPDFLGSIEGPFSGILPS

EDTSHLSVNSLNDTSPSNSDFCEEFYTTFIDTKANGDAATTNTITQSLTEILSEPIDLSD

FSLCKAFNGNHSGTVPECNDSDSGISLNASSSVASPEHSVESCAYGDKTLGCSDSEMEDV

DSAPGSVSQSNASVYSLQFQDPVLSSMGPNTQTPSLPCTNTVKKEPPAAPGHPKPPFTKD

KSSSRLEAHLTRDEQRAKALQIPFPVETIINLPVDDFNEMMSKEQFNEAQLTLIRDIRRR

GKNKVAAQNCRKRKLENIVELKQDLSDLKDEKEKLLKEKGEHDRSLRQMKKQLTTLYLEV

FSMLRDEDGKPYSPSDYSLQQTTDGNVFLVPKSKKSETKL

>av_c_cristata gi|698430665|ref|XP_009697172.1| PREDICTED: nuclear factor erythroid 2-related factor 2 isoform X1 [Cariama cristata]

MNLIDILWRQDIDLGARREVFDFSQRQKEYELEKQKKLEKERQEQLQKEQEKALLAQLEL

DEETGEFVPVQPAQHIQSENTEPPIVFSQTTQTSKPEAEALSFDDCMQLLAEAFPFIDDN

EASSPAFQSLVLAQINSNPVFISSDQTQPPESPVLDPLTDAENMQNIEQVWEELLSLPEL

QCLNIENDNLAEISTIASPETKPTEMHNSYNYYSSLPIMRKDVNCSPDFLDSIEGPFSSI

LPPEDTSQLSVNSLNDASPSNSDFCEDFYTTFIDTKVNVDMVMTNTISQSSLADILSEPI

DLSDFSLCKAFNGNHSGTVPECNDSDSGISLNASSSVASPEHSVESSAYGDKTFGCSDSE

MEDMDSAPGSVPQGNASAYSLQFQDQVFSSVGPSTQTPSLQCTSTPKKEPPAGPGHPKAP

FTKDKPSSRLEAHLTRDELRAKALLIPFPVEKIVNLPVDDFNEMMSKEQFSEAQLALIRD

IRRRGKNKVAAQNCRKRKLENIVELEQDLSNLKDEKEKLLREKGEHDKSLRQMKKQLTTL

YLEVFSMLRDEDGKSYSPSEYSLQQTRDGNVFLVPKSKKSETKL

>av_p_crispus gi|694650372|ref|XP_009481973.1| PREDICTED: nuclear factor erythroid 2-related factor 2 isoform X1 [Pelecanus crispus]

MNLIDILWRQDIDLGARREVFDFSQRRKEYELEKQKKLEKERQEQLQKEQEKALLAQLEL

DEETGEFVPVQPAQCIQSENTEPPIGFSQTTQTSKPEAEALSFDDCMQLLAEAFPFIDDN

EASSAAFESLVAAEIDSNAVFISSDQTQPPDSPVLVPLTDAENMQNIEQVWEELLSLPEL

QCLNIENDNLAEVTTITSPETKPTEMHNSYNYYSSLPIMRKDVNCGPDFLDSIEGPFSSI

LPPEDTSQLSVNSLNDTSPSNSDFCEDFYTAFIDTKANGDTATTNTISQSLAEILSEPID

LSDFSLCKAFNGNHSGTIPECNDSDSGISLNASSSVASPEHSAESSAYGDKTFGCSDSEM

EDMDSAPGSVPQSNASVYSSQFQDQVFSSVGPSTQTPSLQCTNTPKKEPPAGPGHPKALF

TKDKPSSCLEAHLTRDEQRAKALQIPFPVEKIINLPVDDFNEMMSKEQFSEAQLALIRDI

RRRGKNKVAAQNCRKRKLENIVELEQDLSNLKDEKEKLLKEKGEHDKSLRQMKKQLTTLY

LEVFSMLRDEDGKSYSPSEYSLQQTRDGNVFLVPKSKKSETKL

>av_t_guttatus gi|719754029|ref|XP_010213995.1| PREDICTED: nuclear factor erythroid 2-related factor 2 [Tinamus guttatus]

MNLIDILWRQDIDLGARREVFDFSQRQKEYELEKQKKLEKERQEQLQKEQEKALLAQLQL

DEETGEFVPIQPDQRVESENTEPPDSFSQSTHTSKPETEALSFDDCMQLLAEAFPFIDDN

EVSFTTFQSLVPAQMDSSPVFMSSNQTQPEVESPESPALVSLTDAENMQDIEQVWEELLS

LPELQCLNIENDNLGEVSTITSPEPKSTEMHNSYNYYNSLSTMKKDVTCGPDFLHSIEGP

FSNILPPEDTSQLSGNSLNHTSNSSSNFCEDFYATFIHTKENSNTATTNTISQSLVDILS

EPIDLSSFSLCKAFNGDHSGTAPECNDSDSGISLNANSSIASPEPSVESSVLGDKALGCS

DSELEEADGAAGSAAPSSARACARPFPERALCALGPGPQRPALPYANAPKKEPPASPGHP

KAPFAKDKPASRPGAPLTRDEQRAKALQIPFPVEKIINLPVDDFNEMMSKEQFSEAQLAL

IRDIRRRGKNKVAAQNCRKRKLENRVELEQDLSNLKDEKEKLLKEKGENDKSLRQMKKQL

TTLYLEVFSMLRDEDGKSYSPSEYSLQQTRDGNVFLVPKSKKSETKF

>re_a_carolinensis gi|327284173|ref|XP_003226813.1| PREDICTED: nuclear factor erythroid 2-related factor 2 [Anolis carolinensis]

MEVEMPQDMNLIDILWRQDIDLGARREVFDFSQRKKASELEKQKKLERERQEQLQKEQEKAFLAQLQLDE

ETGEFVPIQPTQAIESGNTAISNNYSQNVHISKQDADNLFSDVFDDCMQVLAETFLFVEDPKVSPVEFQQ

VAPSDIESNQVFVDPNHMQPLDSSVLQPAISEFAMTPGESTQDMEQVWEELLSIPELQCLNIQNDNLAEV

TPNTCTANTMSEAAIDFTFYNPLPPMENEVSTCSPEFLKPLEASYSGILLPDLSQNNTSSTSSDFCEDFY

PDFIDVKANNSITSPPPNFVDQALTGFLNEPIDLSDFAQCKAFNCDLAGNPQECTDSDSGISLNRSPSTT

SPAHSIDSSSICRDTGFGCSDTEIEEMDSAPGSVQQSNTQMPVFQFLLPLSPPVEQRSPTAASPVKGEVK

RELPANPGHSEAPFMKDKSYSQDEAHLTRDELRAKALQIPFPVEKIINLPVDDFNEMMSKEQFTEAQVTL

IRDIRRRGKNKVAAQNCRKRKLENITELEYDLGYLKDEREKLLKEKAENDKSLHLLKKQLSTLYLEVFGM

LRDEDGKPYSVNEYSLQQTRDGGIFLVPKTKKPGTKM

>re_c_mydas gi|465978850|gb|EMP35077.1| Nuclear factor erythroid 2-related factor 2, partial [Chelonia mydas]

YHLSYPQDMNLIDILWRQDIDLGARREVFDFSQRQKEYELEKQKKLEKERQEQLQKEQEKALLAQLQLDE

ETGEFIPIQPAQHIESDNTGMPTNFSKTTHISKPETDALSFDDCMQLLAETFSYVDDDEVSSAAFQVLVP

AHIDSDAIFITSNQTQPPESSVLQSSVAEVDNMQNIEQVWEELLSIPELQCLNIENDNLAEVTTIANPET

KPSEIHSNFYNSSSITVNCNSDFLNTFEDSFSSILPPEDLSQLGLDSLDTTSCLSSNFSEDFYSTFVDPK

VNGDTAAPHVVSESFAEIPYEPIDISDFSLCKAFNGDHQGNAPECNDSDSGISLNAHSSTASPEHSVESS

VFGDTAFGYSDSEMEEMESAPGSLQQSNAQMYSLQFHDPVSPSLGPSTKKSDLQCVNTPKRELPASPGHP

KAPFTRDKPSRYPEAHFTRDEQRAKALHIPFSVEKIINLPVDDFNEMMSKEQFNEAQLALIRDIRRRGKN

KVAAQNCRKRKLENIVELEQDLGHLKDEKEKLLKEKGENDKSIRLMKKQLTNLYLEVFSMLHDENGKPYS

PSEYSLQQTKDGSIFLVPKSKKPETKF

>re_p_bivattatus gi|602635604|ref|XP_007424667.1| PREDICTED: nuclear factor erythroid 2-related factor 2 isoform X1 [Python bivittatus]

MVVAWSGRARGRRMGPRRKVVLDVTLIDILWRQDIDLGAGREVFDFSQRKKEYELERQKK

LESERQEQLQKEQEKAFLAQLQLDEETGEFVPIQPAQAIESGNSAISNSYSQSVHISKQD

ADDLFDDCMQILAETFPFVDESEISPAEFQQVAPLEMATNQVFVDSNHMQPLDSSVLQST

IPELLDTKVENTQDIEQVWEELLSIPELQCLNIQNDSLADVTPNSFAVNATSEAADNFTL

YNSLSAMEKEVNCSQEFLKPLEDPYSSMVLPEDPSQHSTDFCEDFYSLIDGKMNSRVATP

PSHFVDQALAGFLSEPIDLSDFAQCKAFNQDLAGNAPECNDSDSGISLNASPSTTSPAHS

VESSSVYRDTSFGCSDSEMEEIDSAPGSVSQSNTKMHSFQLQAPVSPSLEQGTPKPDIPL

TSTPQRELPANPGHVEAPFMKDKSFSQPEAHLTRDELRAKALQIPFPVENIINLPVDDFN

EMMSKQQFSEAQLTLIRDIRRRGKNKVAAQNCRKRKLENITELEHDLDYLKDEKEKLLKE

KAENDKSLQLLKKQLSTLYLEVFSMLRDEDGKPYSPNDYSLQQTRDGSIFLVPKSKKLET

KF

>re_a_mississippiensis gi|564264610|ref|XP_006271149.1| PREDICTED: nuclear factor erythroid 2-related factor 2 isoform X1 [Alligator mississippiensis]

MRSLQGIGDMNLIDILWRQDIDLGARREVFDLSQRKKEYELEKQKKLEKERQEQLQKEQE

KALLAQLQLDEETGEFVPIQPTQHIESENTRAPISFSQNTHTSKSEADALSFDDCMQLLA

ETFSFVDDNEVSSAAFQSLVPAPVDSNTIFISPNQTQPPEASVLQSPVTNGDSTQNINQV

WEELLSIAELQCLNIENDNLVQIMNAFTSPEAKPAEMHNNCNFYNSLSITDKDVTCNPDF

LDAFEEGPFSSILPPEDLSQLRVNPSNTTPSSRSDLCEDFYSTFIDTKVSNDTAAPNVIS

QSLDDILSEPIDLADFSLCKAFNSDLSGSAAEGNDSDSGISLNTSPSTASPEYSGESSVC

EDKTFGYSDSEMEETDSAPGSMQQSNVNVYSLQCHDQMSPALGPSTRKSDLQCANTPKGE

LPASPGHPKAPFTKDKPSGHLEAHLTRDEQRAKALHIPFPVEKIINLPVDDFNEMMSKEQ

FNEAQLALIRDIRRRGKNKVAAQNCRKRKLENIVELEQDLGHLKDEKEKLLKEKGENDKS

LRLMKKQLSTLYLEVFSMLRDENGKPYSPNEYSLQQTRDGNVFLVPRSKKPDTKF

>re_n_annah gi|565323069|gb|ETE73686.1| Nuclear factor erythroid 2-related factor 2, partial [Ophiophagus hannah]

MTFLLYPFCLLEDVTLIDILWRQDIDLGARREVFDFSQRKKEYELEKQKKLESERQEQLQKEQEKAFLAQ

LRLDEETGEFVPIQPAQVNVHISKQDTDDLFDDCMQILAETFSFVDESEISPSEFQQVAPLEMAANQVFL

ESNHMQRLDSPVLQSTIPELLDTKVENTQDIEQVWEELLSIPELQCLNIQNDSLADVTSSPFAVNTTSEA

TDDFNFYNSLSVMEEEVVNGSQDFLKPLEDPYSSIGLPEDSSQRSTDFCEDFYSFIDVKMNPRVATPPSH

FVDQAIAGFLSEPIDLSDFAQCKAFKQDLVGNAPEYNDSDSGISLNASPNITSPAHSVESSSVCQDTGFG

CSDSEMEEIDCAPGIVSPSNAKIHSFHLQAPASPSLEQGTPKPDLLLTSTPQRELPANPGHVEAPFMKDK

SFNQQEAYLTRDELRAKALKIPFPVEKIINLPVDDFNEMMSKQQFNEAQLSLIRDIRRRGKNKVAAQNCR

KRKLENITELEHDLDYLKDEKEKLLKEKVENDKSLQLLKKQLSTLYLEVFSMLRDEDGKPYSPNDYSLQQ

TRDGSIFLVPKSKKLETKF

>am_x_tropicalis tr|Q68FB5|Q68FB5_XENTR Nuclear factor (Erythroid-derived 2)-like 2 OS=Xenopus tropicalis GN=nfe2l2 PE=2 SV=1

MMEIEMPLPLQSQQDMDLIDILWKQDIDLGVSREVFDYNQRQKENELEKQKKLEKERQEQ

LQKEREKALYAQLQLDEETGEFIPIQQAAPIETAAVTQELASSIEVKPSLVHDLSFDECL

KILGETFQLGPANEESSLAYQTLEPSDPIETNQTFLQSEPNPVPAGTLSSIPAEGEIMHE

MNQAWEELLSIPELQCLNNEIENMVDLSMYTNQESITMTETPDTYSFLSPLSTIEKPHES

STVFSSDLVDTFTSSLPSVNTNTAFNVESFCDDIFTLDPKVTNVVPLTDNSGQLLNELLN

DNVDITDLSLCKAFNGNNQPEFNDSDSGVSVNASPCATSPSQSMSGSVYGEPHHSYSDSD

MEDMDSTPETAQQKPPDNFTAAFTEDTYFTLSPFVSHDTDPFDIEAHTPSAKEIPASPGY

SKAPFAKDKYLSRQEARFTRDEQRAKVLNIPFSVDKIVNLPVDNFNELMSKYQFNEAQLA

LIRDIRRRGKNKVAAQNCRKRKMDNIVELETDLDKLKYEKEKLLAERGEYNNSLSQLKKK

LGALYMEVFNKLQDENGQPYSPHEYSLQQTKEGNIFLVPKTKKVSIKKE

>am_x_laevis gi|147907391|ref|NP_001086307.1| nuclear factor, erythroid 2-like 2 [Xenopus laevis]

MMEIEIPLPLQSQQDLDLIDILWKQDIDLGVSREVFDYNQRQKENELEKQKKLEKERQEQLQKEQEKALY

AQLQLDEETGEFIPVQQATPIETTEVTQALATSIQDKPSAVHELSFDECLKILADTFQLGITNEDSSIAY

QTLQPNAPIETNQNFLQSDPNSVAAGTLSSIPAEGENLNEINQAWEELLSIPELQCLNNEIDNMVDLSMY

PNPESITMTESPDTYSFLNPLSAIAKPHEINTSVVSNDFVDTFASSVPSVNTNAPFNVETFCDDIFTLVD

PKVTNIVPLTNNSGQLLNELLNDNVDITDLSLCKAFNGSNQPEFNDSDSGVSVNTSPCATSPSQSIGGSA

FDEPHYGYSDSDMEDMDSTPEAAQQKPPDNFIAPFTEDTYFTLSPFVSQDTDPFEIEAYDSPAKEIPASP

GYSKAPFAKDKSLNRQEARFTRDTQRAKVLNIPFSVDKIVNLPVDSFNEMMSKYQFNEAQLALIRDIRRR

GKNKVAAQNCRKRKMENIVELETDLDKLKYEKEKLLSERGAYNNSLSQMKKKLGTLYMEVFNKLQDENGK

PYSPHEYSLQQTKEGNIFLVPKTKKVSIKKE

>fi_d_rerio gi|528489101|ref|XP_005172568.1| PREDICTED: nuclear factor erythroid 2-related factor 2 isoform X1 [Danio rerio]

MMEIEMSKMQPSQQDMDLIDILWRQDVDLGAGREVFDFSYRQKEVELRRRREQEEQELQERLQEQEKTLL

AQLQLDEETGEFLPRSTPLTHTPEADGGGAGEITQNGAFAEQEADPMSFDECMQLLAETFPLTEPAESAP

PCLNTSAPPSTDLMMPADVPAFTQNPLLPGSLDQAWMELLSLPELQCLNMQMQETLDMNAFMKPSTEAPT

QNYGQYLPGMDHLGSAQTEVCPPEFTNTYNGSFNTMVSPNMNQLSLNVPDVGAEFGPEEFNELFYPEMEV

KVNNPPITSDGGNMVGDPPVNPIDLQSFSPGDFSSGKPDPIVEFQDSDSGLSLDASPHMSSPGKSITQDG

SFGFSDSDSEEMEGSPGSMESDYNEIFPLVYLNDGSQTPLSEKSSTEKQEMKLKNPKMEPAEASGHSKPP

FTKDKLKKRSEARLSRDEQRAKALQIPFTVDMIINLPVDDFNEMMSKHQLNEAQLALVRDIRRRGKNKVA

AQNCRKRKLENIVGLEYELDSLKEEKERLMKEKSERSSNLKEMKQQLSTLYQEVFGMLRDENGKAFSPNE

FSLQHTADGTVFLVPRLKKTLVKNI

>fi_c_milii gi|632945823|ref|XP_007888253.1| PREDICTED: nuclear factor erythroid 2-related factor 2 isoform X1 [Callorhinchus milii]

MRAGGVRHRPRSEPLRGTGRAAPGVFYAESQQSVVSRVSQTATGNNMTDIQRLPIQQQSQQDRDLIDILW

RQDIDLGVVREDFDYNYRQKEYALEKQKKLEKEKQEQLQKEQEKALLAQLQLDEETGEFVAIRPAKNSEP

ANTEGLTESIQIPRTAEQDNEALSFDECMQLLAEAFPFVEDIETVPLEATVPLEPSVPIPAQTSSQQMAP

HETQQAETSVLPSAAPESNSLEDLEQTWQELLSIPELQWLHMQNEHFGGTAGFSSRSKASEIQSDGYIAT

LPSDQMVTESNHSFLSLFDRPYQEIMPPESQDMIQLKTNASDNANSSFSNNFNGLFCSTFVNTQRNSNLS

PLTTMTDSLTGILDDPLLEQITISDLAMNENFDCKQPPNFPEVPDSDSGLGSSPNTASPHNSMGSSICGD

APYSHGDSDMDDLESSPDSVKPEFPEMYPMQYQNEDQYQTPSLQDLTKPSPCLNLDTRQTPKDELPVSPG

HRKAPFTKDKHSKRVEARLTRDEQRAKALKVPFSVRKIINLPVDDFNEMVSKYQLNEPQLALIRDIRRRG

KNKVAAQNCRKRKLENLVGLEQDLDSLEDEKEKLLEEKGEHNKSLHIIKQQLNSLYREVFSMLRDGDGHP

YSPSEYSLQHTSDGSVFLVPRSKKLEIKRE

>fi_l_chalumnae gi|557007423|ref|XP_006005031.1| PREDICTED: nuclear factor erythroid 2-related factor 2 isoform X1 [Latimeria chalumnae]

MMEIQLPPTQQNQQDIDLIDILWRQDVDLGARREVFDYSHRQKEYELEKKKKLEKERQEQLQKEKEKSLL

ARLQLDEETGEFVPIQPAQQFEPEPSPVPTEPAQNTSISEQESEALSFDECMQLLADTFPFVDDIKVENS

PVFVAPRQTDSSILHPTMPDPNSMQDVEQVWQELLSIPELQCLNIQSENMADQTTTTSTAGTDNDNCSFF

TSLVLMDKPVLGCSPQVPNTFENSYHTLVQPENLNQIGVSLLNTNVSQSCDSFCNIFYSSLLSPENENNI

PPVNNANHSLTGPLDESPLKSIDIVDLSVCKGFEADCSTDMSEFPDSDSAMSIDASPNATSPVNSAKSSM

YGDAPFGYSDSEMEDMDSNPGSVQKNYSICPTEFQGDAQYQTAPSLVPQKQTFNLQATRSPRKEVPASPG

HNKAPFTKDKISSRIEARLTRDEQRAKALNIPFSVEKIINLPVDDFNEMMSKHQFNEAQLALIRDIRRRG

KNKVAAQNCRKRKLESIVGLEHELNQLKDDKEKLLKEKREYDKNLRLIKQQLNSLYHDVFSMLCDEDGKP

YSPSEYSLQQTSDGSVFLVPRVKKPEIRRE

>fi_o_formosus gi|820162421|gb|KKX22618.1| nuclear factor erythroid 2-related factor 2-like [Scleropages formosus]

MELIDILWRQDIDLGAEREVFDYSHRQKEHELRRQRELEEEERQQRLREQEKALLAQLQLDEETGEFVPR

PLPTAQPGPVAAQTAVFAEDGGDTLSFDECMQLLEETFPLVETIESDSSPGMMGPPPASLLSAVQSTQKP

LPDLEQAWMELLSIPELQGWHGAANPLVPPLFPGSEASGDYPFYTPKLTDVVASPAEAGPPPAYQDTFEG

SFAAIAPPENLSQMTLKASDVNAAFNVDSFCDMFYPDLTNANAKKPSALAADGNESGPLAEVQDEPANPM

EIPEFALSEGFEDKKAEVMAEFPDSDSGLSLDASPSGCSPQKSAFGDGSFGYSDSDMEEMDSNPGSMQSE

YAEMFPSSFGADGFQGDSSIAAQPSPSQEPNVKHTKTESVDGVGHSKPPFAKDKQKRRAESRLSRDEQRA

KALQIPFTVDLIINLPVDDFNEMMSKHQLNEAQLALVRDIRRRGKNKVAAQNCRKRKMESIAGLESELDS

LREEKERLQREKAENDSSLRRAKQQLSSLYQEVFGMLRDEEGRPYSPSEYSLQQSSDGNVFLVPRIKKTS

AKSSSK

>fi_b_floridae tr|C3XTB7|C3XTB7_BRAFL Putative uncharacterized protein OS=Branchiostoma floridae GN=BRAFLDRAFT_127500 PE=4 SV=1

MVKKHFYDGLVQLAILFSLLGTDINSYLNSQDQNQGTSLQEIIQGQNLALTQSPSPFNPN

LLTGYLNLKSAHHDRYEREREILQELHKLKPSSKAFPLDITALLLEGGVQTEEGTHVTAE

EPSSQQAEPNLGNPAENEVEGATGPSPVGDLTKEDMDLIKVLWQQDIDLGAGLEVFDAGL

RQQELEKQRALEKEKEECSKAQEWQEGVDYFVDSETGEHIPLPPRQPEMAPQQPSTEPIM

TMDQCMQYLQSMIPVPEAEQPQVPSPPATVPDLDQTLQDLASIEELGLGLPQQYSYSHNT

QDTVSQPSPVSNINTNVSLVNAINATLDDGLLPPADLPSDAQNLTQMPIMDNFDLNMTQL

LLNTDPSEFDQLLSGGMLDDTSLMDVDFNNISEAGSDSGLSLHDNETSSMSEHESSSAYD

MSSNCDGSSECDMDMLGATGVSRTELQDINFEEGDAEEGAVGYQPVFKSCGMEAISNHSY

SGNQPGDQPSFQASKEHVNHNHTYPLPPGSEKQHMRRGGSSRRNGTPPRGSDSDSKAGRR

RSKDERRAKQLKVPFSVDRIISTPVDDFNDMLAQHPLTDAQLQLIRDIRRRGKNKIAAQN

CRKRKIDTIYTLDDDVKQLMEDKERLIKEQGMIDKSLRSMRDKFDKLYQEVFRSLRDDNG

QPYSPDEFSLQQTSDGNVFLVPTNSTVSSTATRTNPKGKKRKGSKSNK

>fi_p_marinus tr|S4RI79|S4RI79_PETMA Uncharacterized protein (Fragment) OS=Petromyzon marinus PE=4 SV=1

QDSAIPGGEQAPLSFDECLKILEENFNFDTTPEVRERNCTDVSPPPQTQRAIYAGAHAFQ

QCPLHPLDLEQRWQEILSLPDFQVCLPLWLVKVLDMHVVDLEPSLYAGIESQPALDVLES

CPALCCDSTATAVPAQDFNSTLGSVEVPIDSEANYCGMDLPESKYGIVKVTAGNSRIPLN

LPPRESIPGSSDVGKIEIFRPSSSFLAFESTAALDEHDSDSGMSTGSSPHGSHGSHYQAL

FRDEDDEFSDEDDLDEMESVSGERHTYSQEEDGSAFLDEAHYSSSSPGNDEKQRRNPSGK

KSKLSRSDPAASDGSAKRPVARDKSGCYPTSKGGGRLSRDERKAQSLKIPFSVADIVNLP

VDDFVGLLSRHQLNDSQLALVRDIRRRGKNKVAAQNCRKRKMEGLSSLEGELDGLLGERE

RLKRERDQLSSGLASVRQGLEQMQRQVFESLRDDAGQPYSPSKYFLEYASNGTIFLVPRH

PSHVVRPV

>mo_b_glabrata gi|908411096|ref|XP_013066639.1| PREDICTED: nuclear factor erythroid 2-related factor 2-like isoform X1 [Biomphalaria glabrata]

MIKEYFTDGLIGLAILLSIFRTDLVGINNLINYPEVQEIILGQTVAYLPASYNHIINSHH

PFESSKHLELSNEAFSAWHLNINNLPFLRERHRTEIEAFLVSGTQQNVELENFTGTPTTL

NVEHEDNQIVDLSGQNLPEESASSYSEATISNDNVPESSEDTLQTAIADTSIVNNPFPGC

NLTKEDLDLIDVLWQQDVDLGVGKEVFDLFLRQEIETKKEQELLKHQEWEKSQLQLREKQ

EKERQEEAEKWLKENFRRDGETGEWIRNSNGPSSSLDYFETDDSFTLEAALDYLSTNIDH

ALIKNLDISPGIIPSYSSDQLTDNCLIGTPEGQGFQQSQQLLSHQDSLEESLNGLLDFLS

NEPVEDNSASLDQFGIDLNTSNLTSDMKLDQDLDAQTDYLIQNVTMQSQEIQPVANEMNA

TFPFLSTNSSDQMNFDLDSLSFLNSLATMQNGGLDDGELFENIPVDSNETLLNANIPLNE

SMDTFTVLHDNFSQSLGAMASPSSYDGLCDSLDGLEGAIGGSDLSASSQNNITRPYSKVN

RLSESSNDSGYPFQSGFSSSSPASSSASSPAGCHYSNISTSGNDTEHDPQNTQTVARHAV

AHNHTYNTPPGQVPREVKKYAPKEPSRKGPHSRDQRRLEEFKIPYTIDDIIESPVETFNE

MLKSHKLSEAQLSLIRDIRRRGKNKIAAQNCRKRKVNVIVNLSDEMVDLEKARDKLLKER

AEIEKETLKMKEKFGHLYTHIFQSLRDEHGQPYDPNLYSLQQTSDGDVLLVPQSMNRNKY

SNSSSASSSPTSSKDSVNSKKRKSFDE

>mo_c_gigas gi|405951713|gb|EKC19603.1| Nuclear factor erythroid 2-related factor 2 [Crassostrea gigas]

MGLCRLPFRFARHQVTRQQRIATFLVGLSNGGGRILTNSQPQDTQPVNNDNVNESPTTEQSSGTAQEEEN

AEPQEQEEVILCQVDADCSSAVDPSFEDLDLIDVLWRQDIDLGVGKEMFDVNLRRELEREREIELQKERQ

KFKRQILEPLSYDGEWVPMNGRRAPSQPLASPPAVPQNMSPQVPNVAPPNVTNMGQQSMNQTSQFQNYQS

YETGMPMMNSSMPQHLSVLENDYHIPTSNQSYPPQLASPSHQPITSPLHHMSPEQSHLQNYTYNQTQQQS

QGNYHPQERMPQPRYATQENRGYGGNTSLEETWMDLVNILELPSNDSAGMVNNMNGPGRMMTPQNHSNAL

IQNVTMPTPINNTVNTNSFEPQTRSNFTSAPPSEGCSSPLEWNPTSMLFNGSSTGPEPSGGNLTTQVDDI

LSNIIDEGLDDLNITEMALEDAGLGSMQMLDDASSESGISMGGSSGGSPSQDHFSEGAMSPYDGMEGGAR

GGGDFGDGTPDFGKRPRKYNFNGGFNYNENGAESSQSNYSSSSNDTDFQYRPSSTNHIRHNHSYPLQPGQ

EPREFKKYSITDKPKQKGPHCRDKKRLEDLKVPLSMDQIVESPVEEFNEILTHHKLSESQLQLIRDIRRR

GKNKVAAQNCRKRKMDVIVTLEDEMTQLKESREKLMAERQMVDKQTRDMKDKYSALYREIFLSLRDEHGR

PYDPAQFSLQQSSDGNVMLFNGSSTGPEPSGGNLTTQVDDILSNIIDEGLDDLNITEMALEDAGLGSMQM

LDDASSESGISMGGSSGGSPSQDHFSEGAMSPYDGMEGGARGGGDFGDGTPDFGKRPRKYNFNGGFNYNE

NGAESSQSNYSSSSNDTDFQYRPSSTNHIRHNHSYPLQPGQEPREFKKYSITDKPKQKGPHCRDKKRLED

LKVPLSMDQIVESPVEEFNEILTHHKLSESQLQLIRDIRRRGKNKVAAQNCRKRKMDVIVTLEDEMTQLK

ESREKLMAERQMVDKQTRDMKDKYSALYREIFLSLRDEHGRPYDPAQFSLQQSSDGNVFLVPKNVTSEEQ

LEMSKKIKEERDKDSH

>an_c_teleta tr|R7V3M6|R7V3M6_CAPTE Uncharacterized protein OS=Capitella teleta GN=CAPTEDRAFT_19335 PE=4 SV=1

MEILHQGIQQPTDMDLIEVLWRQDIDLGAGRETFDINLRKDLEKQREVELEKERQEQLALEGQRVQETIQ

NEALFDPSAFELDAETGELVATQNQLSPSSSSTEEADETLSVENAMELLEQATSALNVTPSVDSSTISLE

QQLDLLIQSNADLPQNVSDDVNESFSLYDGLEGATAGDQDNHSMDVNMTELLYEGNPEHVAHNHTYPLQP

GQQPKEKKKSEPLGAEYTRDRKKIKAMKLPFSLEDIVESPVEHFNEMLIKYRLTEGQLQLMKDIRRRGKN

KVAAQNCRKRKMEVVNGLEDEVALLKAERDRLANQKKGIHKEFASMKVKYGRLYEEVFRSLRDEEGMPYD

PQRFVLQHTEDGNVYVLPREQEKTPKGKANRKRKSNH

>hc_s_kowalevskii gi|291234365|ref|XP_002737120.1| PREDICTED: nuclear factor erythroid 2-related factor 1-like isoform X1 [Saccoglossus kowalevskii]

MTTVQVIPSPAKDMDLIDVLWKQDIDMGVCREIYDGTYRQKELEKEKQLELAKLKEEKSPSIGDEWSGLE

YGVDSETGEYYLIPHVHEPTSEPEATAADIPLDPDAIDYNLDECIQMLREHALQQNGPQAVDDEGLPLSV

GITSPQILPAEDNATAEQQWQDLASLTELQLGLPPLPPQNDTYAVNATTNGNVNLQNASMSPELNNLTDF

MSVGASLLSPVVQTPEDFTYNANNTDSLLAALLSGAVIGDIDLMNDITMDETLSASLDDIDPLNASMEDE

HVPSENGELTTLLPSVTDDADSAVSVNSGSVSSGINSPYSFDNDWNDTTSSHCNDTSDDGIYDDMEGATA

MDYDSADDDMNAYFAGKQVVKEESQYDKYQRLSTQPPNMDNIKHNHTYPQPHDSEQKQQNNKGNHNTPGY

SGSSSKKDKKHKLSRDEKRAKGLKIPFTTDKIINLPVDDFNEMLSSSSLSEAQLTLIRDIRRRGKNKIAA

QHCRKRKLESISNLSDGLAELKAEKERLCKERRMIDKETISMKDRFQVLYREVFESLRDERGAPYDPEEY

SLQQSTDGNVFLVPNTATQRQQEDRKNKNRRKGRSK

>ed_s_purpuratus gi|780165930|ref|XP_011683763.1| PREDICTED: nuclear factor, erythroid derived 2 isoform X1 [Strongylocentrotus purpuratus]

MIQKKLPYESMLAIALMLSVLRLDWTGYLGEDILVTNSRFHDTIVGPSVGLTETQFHNLYNSAGQTHSKN

IITDSFYYNRSVFRELDSLYEGISSITSSRRRVEVTTFLLDQLESNDRSNGQVMVAQQPTNPTNNHVNNN

MNSGNLEQPMHHDSESDEGFEEMDTGVEGAVGQSHSVSPTPSDELSIEDMDLIESLWRQDIDMGVCPETY

PYKTDLKPGFTEDLKKQGLDQWDYNYNIDGETGEYVAGPRQDFGEEVQRTVQPQEPLPCSQPQQDAQPQQ

DSSLSLEDCLQLLEDEYSPDTAQTELSPGLSQQETEQRWHDLATIPELQGSIPELLPTTPTLNNTQEGYS

TPQQLFIPPVANVTGSLVPEVTELSQPQLLNDVFAVNASTNGMVSLQNATQSQQQPILLPPTRNDSFNNF

PNVDAQSHSTNATASAPNDNMTDYLMQTLQHQMNAVNASAPFPAANLSTAENGSSLLMDLLNSPGSAHPI

NPLDLDFDEQMHDVIAALGEDSESLSNEEDSDGSLFEAEGASGYSSGSDEDDYKGAAGGYGFSRGHERQY

GSDSSNNSYSGQPPKMENVKHNHSYAAPSQSQMQNGNGAQNISFNGTNGNYMKLNRDEKRAKALKLPVCL

EKIINLPVDSFNDLVKKYELTDPQMQLVRDIRRRGKNKVAAQNCRKRKIDAIQIVESTVGELRMERDKLV

KERDSIDKEVNEMQQRYAELCEEVFASVQDEHGSPVDPNDYNVQQMPDGTVYLVPRNSNQRDSDEQSM

>fw_e_granulosis tr|W6UXB0|W6UXB0_ECHGR Nuclear factor erythroid 2-related factor OS=Echinococcus granulosus GN=EGR_01981 PE=4 SV=1

MTGASTSSNQERVFASTPPKLYPDQGSVLGAAACRSNENLCIDDYLIYLQKIGFDDPEEGQIDELNFLFQ

ANHLPISVFQPNAQNRDRESGACQTFLTPTTSTSGAGVKPGPVEVDLTPHLVVSVKPERDDLLLTPQQTS

SVEEVTFDTESSENMTDFTPRKLPVSKKVLGPFESTCSITSRTSAPSEYFALSPPPSNSVVRSLQRNTEL

GSPPSTLFVTQASPSSSRVPIFVGPLTDAATLTRSALSPLPSSLSHNGVLKSLSNVRQTPQPRHHLHFSQ

DRFHQLQNQARRNMTENETFVWPRFDTPRHLCIPGPRGSNSSEDLGHISDTEFCPRVSQTSSWRKKEEEG

WDEEVDQSEGDEGEQDGYERWEEEGEIKFSRIVTSPSVPGSTPRSSFCDRNIRDINILKRLAVPFSYEEV

AYSSNERFREIKSKPGLTFDQITAMLDARRRATNRQAAERCRRVKSAARDSLANQLDRLRLEHADLTRRI

ICTRRRRQQKWDELTSAQNHLLQNLVDSTGTPLDPSQWRIQTTSEGELILVRINVSGDINNRNNEDGRND

DHEETGNSAKLS

>ur_c_intestinalis tr|Q4H350|Q4H350_CIOIN Transcription factor protein OS=Ciona intestinalis GN=Ci-NF-E2 PE=2 SV=1

MATDYFINIQDSDMMQLMYQQDVDLGFRWPSRDPVKNLDEIDEVLNMKKTTDVGEFFVDGETGEQIPISK

VQNNSKQQELNDKVETSQAVEPSTEPTSSYFSIDEYLEILNESIDDIQPQVVEQTNLNQISEESIIIEDL

TDLLTDNENGSTMEQNNAHLWQDMSAIPQLPGKSSEAEGQLGTVNVINTNVSLTNATVESNQTNENTLHQ

QGTRNINLGYKPVAFENEQPFATQPYNVYKTPAFNPTVPCKPAFSSPSYNTSLNGEDGIPMDGCLSLPIV

TSPQGLLHNQINSYQAPMETGDSPPYDEVSPVLSFHGEPGSMFFDEANVNEMLDDIINMNSMNQVYNNST

ESAYLNMNPAVFVPPPLQANPPQISFNDTKSLDGSTSDQLKISETCDSDSGVSMSPHSFYMGHQRMERDS

IDNVPSIKLETSLHETSFATNALKVINHNHTYDGTVGKPRIIKKKPDKSASHIRESRDERKARELNIPFT

LDEIIMSPVEEYNEMLARTPLTTAQQTLIKDIRRRGKNKVAAQNCRKRKIETITTMEEDVDVLRGRKNDL

EMEQDELEARKQNLKSQYNALYQQIFRSLRDESGRPYDPSLYTLEQVEGAVLLVPRNLRNRDHNNSDHED

HVDFTRVKMEKRD

>cr_d_pulex tr|E9G1H7|E9G1H7_DAPPU Putative uncharacterized protein OS=Daphnia pulex GN=DAPPUDRAFT_307821 PE=4 SV=1

MRPSAVALCKGLRLEGLFAVRMPLMRTMSMEHRWQDLANFLSLPDGAAAAAAAANLSSHPGHHHHHHHSG

HHPHAVHHTHHPHHPGHPHHAMHTLGHHPHSPHPAFAHPSYPHPHSPHGSVHPHHHHNSSPVHTHHGYGV

HPASSTATTIHHHHHGYSGLTSAATPVYPNSAVDAGGRVALLQNVSIGSPLTELGGNGSYPSPGLGVGVT

LGSAVTTSMNLTNSTDTMETAVPYKTETTADMHLYYQNNTGEVNQTGDGFFPSIFNEDDLQLMDMAITDT

MYTPRLLDNQPVNHSVGLTSANGGNPAVSSVIPSTVIDSTSDSAVSSMGSERGPSISDGDWIDTSNATDS

NTTSITGATAVLPSGSAYGMEYSSGAKYRHYDYGYGGRSSGLEGASTSGRGSSAGTPVAQKKHQMFGKRV

FHDQSDLPGSPAAVTVVGSPAKFDYGSGASASGGSLYSHPSTPLDSASGMNSMDLKYGCGMEYTPHPHDV

RGMDHVHHNHTYALGSEASGTSQRPVSRDKHRASGSSLRGSVSDTASTPGAGTSGSCSESEAYSRDEKRA

RSMNIPMTVDDIINLPMDEFNERLSKYDLTEPQLSLIRDIRRRGKNKVAAQNCRKRKLDQILTLADEVKR

ARERKIRLNKEREFLSAEKTRIKDRFAQLYRHVFQSLRDSDGQPYSPYEYSLQQSADGNILLVPRATAMA

NGGSTIDPQPPIQQQLNHHQHHHQQHLHNHHSSTSNGQHRPVMIDPSSLTAHMVGNASGGPVGGGSSTAV

LHGVRRKTPDQKNEP

>ar_m_occidentalis gi|391333510|ref|XP_003741156.1| PREDICTED: uncharacterized protein LOC100906298 [Metaseiulus occidentalis]

MYHVSIGLLRKKPFLHSHLLQLLLAVGLLRWSAPPEPWSPLYISNNPSSGSLDALQLAPW

ETYAPQASLIHPKALRHDDGYFDLLETFCDYDRLARAASRGPLIAYLAEDGAPSRAPHAS

TGSTALEECQAETLSREDADLIEILWKQDVDLGIPLEDYRPCNQPASQSVAAASPPPKSV

EGSTVVPVLDTLESKKDFEPSTNEPRVTSIDSETGEPIFEPVAGPSTLDSVLQNNSSSDS

FLDINALMGVVSEELFCADPEWAQMPSYSGVSSPPFNGTDPFGGVLLQNVSMPSSYDASV

TAVNSTMQQASPAAGSTFASTPTAAASGDAPVGCNGCSDLSKPTVTDQHAIRDLFTYNNS

TQSGWDSFELFYEDLAALASNTTNATNSHSESNRLVPLGPTTVSQPASRMSSSSANGGSV

SLKHTSIGDGGYAASDTGVSSMYSDEANEEWMESSSETSHDHDQEQLVSTDVSYHSNSSS

MSFGSAEGCTVPQKKYNFFGRKPYHQTSNGTVDRSIEESHVVPQPLKIAHNPAMDGFLHN

HSYGQEQLSAGYNVPQLPVGVKLETQFHSEPRKQSSASQADGYESSDPSESGAGYIQVTS

AKDERRARELKIPIPTEEIVTLSIEEFNERLTRYELSEDQHALIRDIRRRGKNKVAAQNC

RKRKLDQISALQDEVENFQDTCRSLQNENDELTRRELYAQQRLNQLRDVIANAANSGAIP

KHHGHHKMANSE

>ar_s_mimosarum tr|A0A087U446|A0A087U446_9ARAC Segmentation protein cap'n'collar (Fragment) OS=Stegodyphus mimosarum GN=X975_17025 PE=4 SV=1

MFFSKKISSDLLLLVLLVVRLRLDSDISQNDMWTRELVSRDVFVGSSVAYTQIISRAPYS

SYRRIHPKSLEYNELEETSILFNRLNYFNSVRARRSNLIAYYASSPSDQTAAPSLESSNF

ESNGNSITPGDPSLPTGAEAAPDSVGTSSDRGSSSTEDHSLTREDMDLIEILWKQDIDLG

VTRDAYDSHPSVEVETLKDIEYTKESFQEFFSQDDIKDCEKDESVLDPLSNLNYTIDSET

GEYVFDELSSLNDGEFSGDQLLENEALNGLTQDSYLPFDNSTRLSNLTEAFSPTEFPDFM

PEDFPCESDYLLSEIEDLEKELAPFDSFLETPINHSQPSEMYPNVEECWPESTAMLPLLT

QGSFSPHSVNGYHISSNGAAPFSSALQNRTESFVSMLQNASLEIPTTEDTFQTMPSAVGP

SSIGCAVAESMATLTNDTEPIPTNSIITTAYNPDLPELMYTNQTIAVPQNELLISDLLLD

EDLQLVPLPSAVGQDLPNEDNMDTSNDSVSMTSGKVPSVSDYQDWVDSCSESSSNNGERD

DYGRNNSSINNNFNHHANFERVATGMPSKRQKYFESRNEIGNRGFADVPFEHTGGHDQQY

FSLKNPFPHLNENSISGVATNGAPFRKFDFSPRTASVSNSNFLQHNHSYHLPFTSDDSHY

KPVMRDKSKSSSEDDTCNKDEKKAKELKLPISIDDIINLAIDEYNERLAKYELSEAQLTL

IRDIRRRGKNKVAAQNCRKRKMDQISSLQQDLDSLQSEKLHLRSKQNILLQQKHHFEDKY

AQLYQLVMENTGFKPPGDDDHPIRRPSEVTASTAADVLMAEDDSDLSRGARTKRKFKK

>i_co_d_ponderosae tr|U4U1E5|U4U1E5_DENPD Uncharacterized protein OS=Dendroctonus ponderosae GN=D910_03551 PE=4 SV=1

MIQTPQFHHPHHRAAFQSRMPYVRTVSMEQRWQDLANLLSLPNPGEIHPFGAHHALPNYG

HPHAHAHPATTYDARGVLLHNATLPTPMGDLNSTMPYGNLGGTISNAVATSMNLTNSSEP

MGEPSSAPHYKLEPSNDMMYYQNNTGSEFSNNQTDGFLSNLLNDEDLQLMDMAMNEGMYT

MRMLESNNTVSNMSMNNTAVTTSVSRHDMERLDTSSDSAVSSMGSERVPSLSDGEWCDAG

SDSGHTTNGEPYSQDYQNKYRPFDYSYSNRLSENARMNPVPQKKHLMYGKRFHTSGFSEY

RDPTNSFQQENSTSRAKVPEIKYSCSAEFTHLPRTVDHIQHNHTYHLPAENSGPMQRPVS

RDKGKSRRSEEEHLSRDEKRARALNIPITVDDIINLPMDEFNERLSKYDLSEAQLSLIRD

IRRRGKNKVAAQNCRKRKLDQILSLADEVKEMRDRKMSIMNEHKYSLVELKRMQDKYQQL

YRHVFQNLRDADGNQYSPYQYSLQTAADGSILMVPRSSNGMNGTTERKDPPQNQKD

>i_dp_a_aegypti tr|Q17B61|Q17B61_AEDAE AAEL005077-PA OS=Aedes aegypti GN=AAEL005077 PE=4 SV=1

MEVRRYAGFTDERWSWNWQKNASRVPLSRAVSMEQRFQDLANLLSFPPGMGVGMGVGEMP

PAHPHPHYPPHYSYQANGAIPQHGQYHSHAVLQNASLADIGPTQPYYAPNLGSAVATSMH

LTNSTSETDAGATGYKMDHEMMYYSNTSSEMNHTDGFLNSILDDDLQLMDIAVNEGMYTM

RMLDHNATSSNSSVLGGSLGGSAAAVAAAAAAGLNGPAHLGGLMSSASAAVSSGAMQTSL

NGSTGTHGATGGTTSGDRLDASSDSAVSSMGSERVPSLSDGEWGDGGSDSAQEYHNKYGG

PFDYSYSGSNRLGDGTRQPPVAQKKHHMFAKRYFQEQNTSIPSLPSATNPSATGPTVDPQ

SQLNASIPIKYEFDYMNPASLSHLEGAVGPVTKQEDQTSAHNNPLSSVDMKYPYSLDFSR

QNPASAPAARSHHHDVIHHNHTYTLPHNSGANPKPQTRDKRIRKAEEEHLTRDEKRARAL

QIPIPVQDIINLPMDEFNERLSKYDLSETQLSLIRDIRRRGKNKVAAQNCRKRKLDQIVT

LADEVKDMKMRKERLLRDREIIQTERKRIRDKFSALYRHVFQNLRDSEGNPYSQEHWSLQ

QSADGTVVLVPRSVDRQQDLTDRKSETGP

>i_dp_d_melanogaster sp|P20482|CNC_DROME Segmentation protein cap'n'collar OS=Drosophila melanogaster GN=cnc PE=2 SV=3

MISNKKSYAMKMLQLALALSLLHYNPDYLLHRWDSQLELGTHGDGWELEMLRTVHRLDMD

HNPYGNRKGLSPRIEDLLNFDDPSLGGMANGIGGCKLPPRFNGSTFVMNLHNTTGNSSVQ

TAALQDVQSTSAAATGGTMVVGTGGAPTSGGQTSGSALGEIHIDTASLDPGNANHSPLHP

TSELDTFLTPHALQDQRSIWEQNLADLYDYNDLSLQTSPYANLPLKDGQPQPSNSSHLDL

SLAALLHGFTGGSGAPLSTAALNDSTPHPRNLGSVTNNSAGRSDDGEESLYLGRLFGEDE

DEDYEGELIGGVANACEVEGLTTDEPFGSNCFANEVEIGDDEEESEIAEVLYKQDVDLGF

SLDQEAIINASYASGNSAATNVKSKPEDETKSSDPSISESSGFKDTDVNAENEASAASVD

DIEKLKALEELQQDKDKNNENQLEDITNEWNGIPFTIDNETGEYIRLPLDELLNDVLKLS

EFPLQDDLSNDPVASTSQAAAAFNENQAQRIVSETGEDLLSGEGISSKQNRNEAKNKDND

PEKADGDSFSVSDFEELQNSVGSPLFDLDEDAKKELDEMLQSAVPSYHHPHPHHGHPHAH

PHSHHHASMHHAHAHHAAAAAAAHQRAVQQANYGGGVGVGVGVGVGVGSGTGSAFQRQPA

AGGFHHGHHQGRMPRLNRSVSMERLQDFATYFSPIPSMVGGVSDMSPYPHHYPGYSYQAS

PSNGAPGTPGQHGQYGSGANATLQPPPPPPPPHHAAMLHHPNAALGDICPTGQPHYGHNL

GSAVTSSMHLTNSSHEADGAAAAAAAYKVEHDLMYYGNTSSDINQTDGFINSIFTDEDLH

LMDMNESFCRMVDNSTSNNSSVLGLPSSGHVSNGSGSSAQLGAGNPHGNQANGASGGVGS

MSGSAVGAGATGMTADLLASGGAGAQGGADRLDASSDSAVSSMGSERVPSLSDGEWGEGS

DSAQDYHQGKYGGPYDFSYNNNSRLSTATRQPPVAQKKHQLYGKRDPHKQTPSALPPTAP

PAAATAVQSQSIKYEYDAGYASSGMASGGISEPGAMGPALSKDYHHHQPYGMGASGSAFS

GDYTVRPSPRTSQDLVQLNHTYSLPQGSGSLPRPQARDKKPLVATKTASKGASAGNSSSV

GGNSSNLEEEHLTRDEKRARSLNIPISVPDIINLPMDEFNERLSKYDLSENQLSLIRDIR

RRGKNKVAAQNCRKRKLDQILTLEDEVNAVVKRKTQLNQDRDHLESERKRISNKFAMLHR

HVFQYLRDPEGNPCSPADYSLQQAADGSVYLLPREKSEGNNTATAASNAVSSASGGSLNG

HVPTQAPMHSHQSHGMQAQHVVGGMSQQQQQQSRLPPHLQQQHHLQSQQQQPGGQQQQQH

RKE

>i_ho_a_mellifera gi|328792851|ref|XP_003251788.1| PREDICTED: segmentation protein cap'n'collar-like isoform X1 [Apis mellifera]

MLCIKKLYHEELFQLTLLLSLLRIDPESYLGLDIQTIGVGSLDLNNGSRWHTDVHTIVHRPIFVHPKNLD

SMLLNYERDLFEDLNSLGRYNRINSGLNDIHAYLLNVEESTRDIAIAGPSISLSTDPTRNMTSPDSSNSS

QNPDEPTNTAELTQEDMDLIEVLWKQDVDLGFTLVEPTTTATKKLSTVEKGSDDEIEKLKALEAINGSNE

EKDTKEYDEAQDDPWAGLPYTIDLETGEYILNSGNQEGDGNNAIEEDDRLLREASLDLDNHPLAGLTDDS

LGLTDTLELENDLPSDLLGGSLLASANVESLLNNDSLDLPDGFNLEEALQLVGLDEAQSEETKPEVKKKE

KDSIEESTSEAKDDESPIISSSSSVEVAKSSRCEDPETGDMIHTPQFHHPHHPHHRSFQGRMPFMRAMSM

EQRWQDLASLLSLPGAPEHFPHTHPGYPGHGISHSHYEAQRNVLLHNATLAPPVGDLNSTSPYHNVGGSS

NLGSAVATSMNLTNSSEPMGAESGAAYKSEPADMMYYHTPTSDSINQTTDGFLSSLLNDEDLHLMDMAMN

DGMYTMRMLDNGNNNASGPTGAAALSGVQTAGGTTSSATGVTTLPGVTDERMDASSDSAVSSMGSERVPS

LSDGEWMETGSNSSHTQADSHYTMDYASKYRMSYDCSYSVSGRNAGSPRCQTERTMPPVAQKKHQMFAKR

YFQEQGTGSPLGATAHPTTPMKYEYDSHTVGAGAPGNAYSGPIEGATGPQPEIKYSCSVDFSRHQSGRSA

IEHVHHNHTYHLPAESSGSLQRPVSRDKKVRKNDGEEHLTRDEKRARALNVPIPVNDIINLPMDEFNERL

SKYDLSEAQLSLIRDIRRRGKNKVAAQNCRKRKLDQIISLADEVKEMRDRKMRLVREREFMLIERQRVKD

KFSQLYRHVFQSLRDPDGNQYHPYEYSLQQSADGNVLLVPRNQTNPHHPRSTTMEPKTKPDPEHKE

>i_lo_d_plexippus tr|G6CUG7|G6CUG7_DANPL Cap-n-collar OS=Danaus plexippus GN=KGM_18938 PE=4 SV=1

MSSSAIYLVPLDIIAHGGAGYAPNYHAPIPPIPEKHHEAYGAPAPLDGAYKVEAAHHPQQHDGLYYQTPT

EPQQDGFLQSILNDEDLQLMDMAMNEGMYTMRMLDGAPTVHQTHAHMPVAAERDSASDSAVSSMGSERVP

SLSDGEWCDGSDSAQEFHSSKFRPYEAAYGRERSHAPQKKHHMFGKRSFQEQPSQETRPVVKYECEQTYH

EMHMHADYTPRQHIPPQLGVQPTLDINSPHSSHALQHTTLPSPNPPRFGFSSGDRVRHNHTYSAALPPTE

ERLPTRDKRGLHISTYISVFCQRVIRRLTDGSTSDSGSGHLSRDEKRAKALGIPLEVQDIINLPMDEFNE

RLSKHDLSEAQLSLIRDIRRRGKNKVAAQNCRKRKLDQITSLADEVRTVRDRKARTQRDRHNLLADRQKL

KERFAALYRHVFQHLRDPEGRPLSSSQYSLQQAADGSVVLVPRMGGDHSMNRTEEDLERKNNYEH

>i_ph_p_humanus tr|E0VYB8|E0VYB8_PEDHC Putative uncharacterized protein OS=Pediculus humanus subsp. corporis GN=Phum_PHUM512570 PE=4 SV=1

MRRTGWGEGSSSNLGSAVATSMNLTNSSEPISDGSSVFKMENPHDLMYYQNSTSEMNQTTEGFLSSILND

EDLQLMDMAMTEERNEEDRIGHAGRMPFVRTMSMEQRWQDLANLLSLPGQEGVGVHHPFAHHSHSHHHHH

HHHHHGNYNGHNYSHDGRGVLIHNATLAPPVGDLNGSGPYNNSSTTMGSSSNLGSAVATSMNLTNSSEPI

SDGSSVFKMENPHDLMYYQNSTSEMNQTTEGFLSSILNDEDLQLMDMAMTEGMYPMRMLESNSSHNGTTT

GGPPVGHTEERLDASSDSAVSSMGSERVPSLSDGEWMETGSDSGHNTGDHYGNVDYHGSKFRPFDYSYTG

RPLLGNTGPQSATPDGHIPPVAQKKHHMFGKRYFQEQGNATTGSTLPPHRALTLLPTATPTPAPVKYEYV

ETGAEAIVPPGFNNTVEPSCGNKMPEVKYSCSLDFIRHHQTGARSLEHIHHNHTYPLHAEGSVSMAARPS

HREKTNSRGRKSEEDHLTRDEKRANAMNIPMPVEEIVNLPMDEFNERLSKYDLSEAQLSLIRDIRRRGKN

KVAAQNCRKRKLDQIISLADEVKQMRDRKHRLLRERDYMVAERLRVKEKFSQLYRHVFQALRDPEGNQYS

PYEYSLQQSADGTVVLVPRSTSNTLLDQDGGTNRVKHGKDQNHHESHQQKE

>cn_a_digitifera lcl|adi_v1.14913 unnamed protein product

MAVGKKYFNSANLLNVTMALSLLRPDLQGFLGQNTTPPMPVIAYPHNYSGVAFAPSSTSNFNFNFHLGSK

EAVAQDRNPLGEFLDWHTTSQNQLDTDAVAMLFNGESHAPRETRTSFSTELDSGYCSDGGRSPSALSSVS

GSPRHDIESSCGNFQAPETLNNVGASAASGFSLADQDILKGFELEDYIDFSIYDEPPHKKSFTPEQQQPF

YPIVKTEPLSPVDSYTRKGPNSPLDTSYVPTDFNQKDPFEEISLEEPFSIDGFDQLDPYSIDFISTNEEF

QEVGAEGDTGFDFESFANNLDYSNDLPPDIFDRRPVIEAAIESKHDSLVPPGQEPIPVFPEVTATRNDSG

GPFKELLPLTQSFSGDWESAPLPLLSSVTVKQEKPDFQDQSASAPLRFSPAKVPREVYKDARPKVASSST

ASRIATENEIVDMNISEFNSFLETLSEAEAQKARDIRRRGKNKVAARLCRKRKIELVGDIEDDIESLKQK

KEEILKERKKLQEEQSYYQNKISELQDHLFKSLRDESGKPLSSKEYSLFQGANGSVYVGKNIDSESRKTR

SGGGE

>cn_h_vulgaris gi|221129532|ref|XP_002160548.1| PREDICTED: uncharacterized protein LOC100209530 [Hydra vulgaris]

MIWDQGVSNFIVALLFTKWILQSSNDTLQINQIFNNVPFNKRLVSTFRNLYKNAENTQLDLNIAGYHDLQ

NLKGSNVYSYEVQNLLSWQNAYHRGDLRLHSELQLSILPVNAVHKGDKKISNSLQTISNVFIDDGYDSSG

LSLDSPTSSTLSETRPDYLFSSSSPPFSDNNFENFSSLPGAYSADFDIDDLLDNNDLNLGVETNYKSQSC

CNEKEKLSCLNPFSKILDIDHDLDYVSSPLKSPSFDEFLTSPNQGFLTKYGIDISFEDPFEICFTNSSLK

EKLLDSSLNIEDYDINELKLEEDVDRALVESLSPNVSKYKVMNDESNEVSKALIEKNAFEVDHDYYQRSD

SSLNYTTKSYLNDVAVGARQLTELSHTFAPSHSVTNSKETKSENIWPDFPYTSEELVTMPVDTFNEVIKL

LDEIRKHIAKDVRRKGKNKFAARGCRKRKNDLIKCLDIGVDELIRKKNNLLDERNKIIAETLEIRRKTMW

LNSYIFMHLRDSNGGLYSSVDYSLQYTSDGNVYIVPSDKTSKVHI

>cz_s_rosetta gi|514699996|ref|XP_004997566.1| hypothetical protein PTSG_01585 [Salpingoeca rosetta]

MTTAAANETFTLRLDDPQPQPQQQLEQAAFADMDFSFLDFVPDDTQAGTQHTTTQQHQHQQQHQHQQQQV

MDDVDLFGGLFTPPGNMDMSATPTFTLPLPEQTTTATTTTTATAQQQQQQVVPPLTVNIPTTPATSQHYH

HQQLDLDLPSLWTPPPQDLVDLSSLHELFGTTTNDHNDDSNGYAATTNTTATTLPQCTRPSSVHSDEHND

DGNVSDAASDISGSSLATSADTEHDGPAAKRAKRSTTAAARTATASSVSKKASPSPSRNTGSKGGSRGRG

HARARSTDSTDSVTAGDESTGDDEEEDYYDDSDPRNRPFASRLRANTHKSLSEEEKRLLKREGFELPEGR

DLTEKERKQLGVVRRKIKNKISAQDSRKKKRRYIKQLESKLKKETTVNVDLKNRVDLLEKQNTTLYEKLM

ELHAHVRRLATSGGSATSGTALLLLGLCFSLYMAPSTPSAQADPTAMYFNTPAPTHDPSAAATATADATA

AAAASSSSAFRARTLLSSPSSSLAWLPDTVATAADTVTQHAREQRKPRGIDAYIWERFTALVAGGDGDRD

RVTPAGLGTGGVVVQELDDDDDDDHVHDESDNVVAEGGRGRQLDKQRQQSHATAATRKAMGNDTLDTLDD

>cn_n_vectensis tr|A7SQR2|A7SQR2_NEMVE Predicted protein (Fragment) OS=Nematostella vectensis GN=v1g127893 PE=4 SV=1

ADSLDIGVSEEKIVEMSVAEFTTFLEKLSDAQAKYVRDVRRRGKNKEAARICRKRKMDAIETLDDEITRL

KQQRQSMFDERKDLQQETAELKRKISELESSLFSSFRDDNGRPLSSEEYSLFQGSDGAVFLGKNIASKEK

KKEKSS

>ne_c_elegans tr|V6CLA3|V6CLA3_CAEEL SKN-1, isoform d OS=Caenorhabditis elegans GN=skn-1 PE=4 SV=1

MQNDSLQAVVSNGQIDYDHSYQSTGQTPLSPLIIGSSGRQQQTQTSPGSVTVTATATQSLFDPYHSQRHS

FSDCTTDSSSTCSRLSSESPRYTSESSTGTHESRFYGKLAPSSGSRYQRSSSPRSSQSSIKIARVVPLAS

GQRKRGRQSKDEQLASDNELPVSAFQISEMSLSELQQVLKNESLSEYQRQLIRKIRRRGKNKVAARTCRQ

RRTDRHDKMSHYI

>ne_p_pacificus gi|802707126|gb|KKA71482.1| skn-1, partial [Pristionchus pacificus]

VQVQAMLSTAQARAAARSARLAAAREYASEVVQNVELQDNLGLEDKILGSLSGIVMEAIDCVRAEQPFRS

SSLSLLSLELLSYLRQPNKPMIVNFACAFTMLMSELEGADLTERGGAEGTGGPRSYSNSIPDVPPEAATV

ERAVPATTFSTPSTSRQHRAHAPPHQPAETIFPRETMSADPLKDVPLLKEEDVIPEQERLSVEPIDYDDH

DNHFGQHSLDGEGFMDEGRSESSYPDDASGQNELLQRMIANFSTPSTSSGHTHRFDRSDSAKNQHKSKSG

RFESRDDQLAATYALPVSAKEIETLTEASMAELMRSGELSEMQRSIIRQIRKRARSKISSREYRKRKEAR

RLELENSLNSSRNPPGQ

>pl_t_adhaerens tr|B3S8P9|B3S8P9_TRIAD Predicted protein OS=Trichoplax adhaerens GN=TRIADDRAFT_60616 PE=4 SV=1

MLSATDLQQFIVGILTDVATLYVCASRQELMPYLKLDDNVIHKMKSPMWDEAVKTMSKYPQSTLQFPSGY

HNSFSHNIHWPTQAVVAFFDVVPDEQNINEQADNGNSNEMPNELANDDEMDDIFSNVEEEITTDYNTNLL

TPGELSSAVLSMKSNTTGEFDWDYWHFGNDDTPNMLPLNGWKGDDNKGIASLTGNAVNGLNDEDSFALAN

IVDDDDNNNLGVSEDWWGQLPTDFVDSPSEGCSNGYRSDGNNTDQSISPSVIAADSDSNSRSEAWDQIKK

HINDDEVLLHSINSLASHQSSQGFLPFIPPNIADISPISRNGFSWDHSGQDDASGFNTNQNDLFHELGLE

LDVLGSQSNNEASLHLNRDGPLSISTTNDNHFLHHGIPNSSFNNATGNPYGEFFDLMGLDNQSPTGELKG

DLPFQPIQASRNGVASNASQPHVPPYSSSSSSSRDAVYALFTDSRPYECNPQTMGATMDMGTFFGYNNDA

IRLENSMVDSHQVASQSQPIGEWASANGTVVYGETQSEFAEIFGVPTNSMLVLPVPERELVDMPVNEFLA

MIERLPSDVAALARDVRRRGKNKFAARNCRKRKIDDIDGLKDEVDELEVQKESLLAEVKKLEEESEKYRK

KSEAMYKKIEKYVKMKEANNRS

>po_a_queenslandica tr|I1G4S3|I1G4S3_AMPQE Uncharacterized protein OS=Amphimedon queenslandica PE=4 SV=1

MVGRGPVQLVYPNNAPDYSNQHPPTKMESFEIPSTIKQEQNVLPPSKFNTGFGLPVGSNGGVPSYSSPSP

PFMQYETGIYVLPIDQLSNNPYKQDGGEDDSQSWLFRKYPTSSSGDASWLPPVTSGDDFNNVLSQVPSTS

SSSSFPFAKEKTVEKDEDFLAQLLDVGGFLDGFDNDSFYSAFNQSNELSIPVQPVAGAIPPLPNHPSPPQ

LSPMQLIPSYQQYDIPQPLEANTEYNGLEDPGLTVPIMAEEHDPLVSGVNEVMPSPSNEVVPISNEHVIA

PPADDLQDLLGELFGKDDFSLSNSLVAPQTERNVIPVGGNGEAFNNFVSPSFIPLSFSPTSDNETNQTRK

TEDNMIPSGDLLPVSKKHRGTSESSCSTLASLLLEERPMDIQLDDEPKKKPSLSSPVSSPSQSSPKDGPA

PSSSRKSHSGGNGIGTVAMFGQNEDEIIHKLMSSHNRRGGASKPITRDKLVIMPVEDFNSLLDEALLSEI

EVAFMKEWRRRGKNKMAAQIARKRKREELFELEDDMDSLRQKKSKLQQSVAKLNALIASYKRRVEVGEKK

IYERYSHAHGSLVSRETHTIHMTDDGKTMLIPRTSDQVLLV

>ct_m_leidyi lcl|ML016353a unnamed protein product [Mnemiopsis leidyi]

MLLRSVRGGHNVRPYYAKRALTRALLRAYKPPRRARYQTSTAIITQNAAEHRSQAMLSESSDSESSGNTT

VQIGEYQFTEAGIVEMNKQQFNDIIQGLPEIQSILLRDMRRRGLTRKAVQKCRQRQNMRLNELDQKKKEL

QEKLRDAVRKQEEMTLEYERARLRYEHAQSRIVEHLRKHPGVHTHKGIVVITPAAAALHRDL

>f_sd_p_nodorum tr|Q0UYB7|Q0UYB7_PHANO Putative uncharacterized protein OS=Phaeosphaeria nodorum (strain SN15 / ATCC MYA-4574 / FGSC 10173) GN=SNOG_03247 PE=4 SV=1

MMEVQQPQQFDWAHDAFTTLNDPSMPLKPEKWASMQNNAFSIPPSLLTQASIERFGQITPPEDSPIKSPL

QSARESSIPVEQLQNEAPWLQQSPPQQQDHTEPSPKRRRTSRQTAASQAQSNDGAPPQHENVDPQPPKRK

RGRPKSQPQMIEAFTADGFPFQVSSARQSHLEKNRVAAHKCRQRKKEYINGLEDRAREFSSKNKALKENV

AMLREEVLELKNEVLRHAGCGFWAVDEYLARCAGDLLGMEVPMQTGGRSMNQTSPLMMDTQQYMNQRHNS

YGSVNSSVTEASSANLNDDFGGLELLRDYEEEDIEDNQ

>f_sg_g_clavigera gi|320591877|gb|EFX04316.1| bzip transcription factor [Grosmannia clavigera kw1407]

MDTDNFFNEYLADVGFAALPHIMHNDHDDSQFFGLGGHDEQQHQIGHFASGKELFGDNDCMDGTVSPSWL

SHLQFEPTSNPESSGDPSPVFSGSIDEDMGQLQADTKHFEDEPEALTHLLLDPEQEKKPESVFLGANSDE

LQALEISIDDVHLKREEVEDMAMSPITSSAATLSVRKRRKRSTAGNNDKVRRQKFLERNRVAASKCRRKK

KEAEQALEGLQKQMEDDHMALKSLRATLVSEFAELQSMLMAHSGCGSASVDQWLATRSTRLEGILGKEAL

DAEFQKPLEDQPGDGADETPTMQHLPPAESQAAHHTSHNAAYQTPRTLLQHSPLATEQFQPSADPVFSAN

YDHSPRLCAGDFTLLPSANKNMRNASLASSFASMTSYASSNFAASDFDAQRKSSTDSTGSSYKLSPISSA

YADFSDSMVVDLTKPEAQMELLAETSFESICSS

>f_sh_c_militaris gi|573987019|ref|XP_006671430.1| bZIP transcription factor, putative [Cordyceps militaris CM01]

MANSRRESFATGPPLFSPKTEDWQSVDMQSIPSNNPYVDPQDAAGYLRMDHEHSHPYSAPSTSSWGMAAQ

TSLPPFDPAVAAGFEIPASIYQNAAHAPMPFPNMFGSMSAEQKHDQASPRSEWPATSQPIHQTATAAAVA

ADGDQLASPGDLRRDGVRKRNARFEIPPDHNLNNIDQLIAESSNEMEIKELKQQKRLLRNRQAALDSRQR

KKQHTERLEDEKKQYTATIGELDMEVNTIRHQLEESRHEAQLYRQYCETLTLQKDELIRNHTIESRELRK

KISVLTENIAALENNSAPTPGPSGNTINGPFGEVDAMCMPGGWDNSNFLHQYGMMPEAPKQSIQPSVAKK

AHSSSPAEGEKTATQGGLLFMLFLVGAFVMSSPSPPAIPHVSEDVRSASAALLDNVLKEAGMPSSTAVQP

LQSQHASVNWGDMSTGAPMTGVLTGGVTAPMLGDLNDFIQPSREQTNEQAFSMSAAQYNGVSDQTFLQNG

HPERVPEQSQGRKNLAAALSEIRATNKQSGSADVYTRTLLWDQIPRDVVRDFIKMFADNNAAQIDPQQCN

EIMS

>f_ss_n_crassa tr|Q7SGM8|Q7SGM8_NEUCR B-ZIP transcription factor IDI4 OS=Neurospora crassa (strain ATCC 24698 / 74-OR23-1A / CBS 708.71 / DSM 1257 / FGSC 987) GN=NCU08055 PE=4 SV=2

MHLSLDHHHLSSDDPYAAASLSHVTDTLSTTMGLTMPSGRNTTTLGMDMFRTASNNSGSSNNNMGYSQLN

ATTSSSSRDHSTTPPTSQSSGSTSPTASTSHHGHGGQGHLYPGLTLPSPVDASSKPKRGRPPGPKKRALS

PSVAAEAELTDSEDIMIKRQRNNIAAKKYRQKKIDRIQELEEEVDQIKKEREELRLMLAKRDAEVGMLRE

MLAMAKQGR

>f_ch_b_dendrobatidis tr|F4NXT1|F4NXT1_BATDJ Putative uncharacterized protein OS=Batrachochytrium dendrobatidis (strain JAM81 / FGSC 10211) GN=BATDEDRAFT_36745 PE=4 SV=1

MCFVKAPRGSATIPTTQLTGEQQQPVCETPFFNNDATLWGDFSSIDSLGQSNSADLAIPLNLAVGPSMGF

SNYHSADTQSSPLLGVDNTTASDSLATLDSLSSNNLDIWLDLLSSSDLGPVAKPLFPVEASTPLAASTGE

PAQTSPVATTQQKYHYNKQLPTHPNTNMLGGALLPSPPESTCPLSPLLPIKVCSTTPTAYSRSAVSSMTP

ASSFLPMSAIQHPSIHQQYSQHSQLHQQRQSQADAHSTATTVSADAKISQTLPVSSTPPLTKRKTDEDLA

AHDDAYNGSNPLAVKRARNNEAARRSRERKMKKLVELEVQVTHLDTEKTDLLVRLAVLESERTTWMHRER

ELAHRVLALETQLSESHRALMHVGLNRNSHESTHFSATDS

>f_mu_r_delemar gi|384500797|gb|EIE91288.1| hypothetical protein RO3G_15999 [Rhizopus delemar RA 99-880]

MNNNNNSSTFTWVLDTNYFSLDNNTIDNDPNAIDVSNDDLFQFLLEPQIQQQQLLNSTTYNNSSNSSDGS

TSSGEEGFTNHKLKSTSSNHGKHSSEFASSTGRLDFRQVQDNLSESQLKLMTSKERRQLRNKISARNFRN

RRKEYVTTLETELEQQKAENSQLKLEIKWLKSKMEKLQGENDKLRLDLVLGPVTLPSSQQQLVVHPSPDM

SLLSATPPSQEDNWDFILPDFNSEQHQNTFISQAVVPTWNQVFLSKEQEQREPSVDLLKQYPLLAPALMS

IILSHTMTMNTDQLIASAKFSNSFIQQQQQQQQQQYIIPSSPNMTNKEAQIIWNLLEPLRVVKERNEKLA

EKSNDKEDDQTNEDAKKEDSKIICPITRCVITWLQYTVCGHISKMIAKHNDTPIEEKPLLCRSYQKAMKY

INA

>f_rz_r_alloycis tr|A0A075B573|A0A075B573_9FUNG Uncharacterized protein OS=Rozella allomycis CSF55 GN=O9G_002094 PE=4 SV=1

MTAPHDYQSHSPSNFDSGSLYHLSSKEQQEILEVSSIIALMGEYAKIHLGTEPPLNNVNIDDTTSEELAY

ILMRMNQDAHKFINFNEINHKGETQNAKARRILNGLVTRLMIEEGLDNEINSSKINISQASQKNSKNKKP

NKQLVKKTKPPKQPISSFLAFYRHKRSEMAKKHPELTFGQPFVDEANKDRIRFADEQEKYRELQAKKSKL

KTNQDEEYNDKDIKKLKPVKENKKIKKYTAENTFITSTASLETVEAENNSNCQDNRLSHGNSERMNLNEP

MVRTSSDHMPINLMSLDLEEFFFQNVKTNTPITSKLYSTSSLDSGRSFEHSDNPDRLDLPPAPTGKRNAI

SINRSFKRSKRDSMTEEQRRELQEKNRIAALKCRKRKKEYVATLKDSIETLEAENLLLEERIKMLESILE

NS

>pl_a_thaliana gi|18420842|ref|NP_568457.1| basic leucine zipper 9 [Arabidopsis thaliana]

MDNHTAKDIGMKRSASELALQEYLTTSPLDPCFDLMNRDYTCELRDSLLWSEGLFPAGPFRDAQSSICEN

LSADSPVSANKPEVRGGVRRTTSGSSHVNSDDEDAETEAGQSEMTNDPNDLKRIRRMNSNRESAKRSRRR

KQEYLVDLETQVDSLKGDNSTLYKQLIDATQQFRSAGTNNRVLKSDVETLRVKVKLAEDLVARGSLTSSL

NQLLQTHLSPPSHSISSLHYTGNTSPAITVHSDQSLFPGMTLSGQNSSPGLGNVSSEAVSCVSDIWP

>bac_synechococcus hypothetical protein, partial [Candidatus Synechococcus spongiarum]

MAQLDGATRHILDNRTRTLAGYLKQALARADDFRFVSAYFTIHGYALLADRLESVGRTRFLFGTPSSVED

LDPGEQEPKAFALTEGGLEPRHVLAQKALARRCAQWVRKNTVEIRAVSRANFLHGKMYLTTSSHGQSGVV

GSSNFTRRGLGEGNQPNLEINLATEDQETVNELREWFDRLWTDDGRTRDVKQQVLAALAHIGNDYAPEAV

YYKTLYEIFHQEIAARQAGDDSATTTGFKDSRVWNALYQFQQDGAMSVIDKLRDHNGCILADSVGLGKTY

TALAVIKYFELCNERVLVLCPRKLYGNWSLYPASNGHRQNPFQEDRFGYRLLAHTDLSRSSGHSGDADLA

NFNWSNYDLLVIDESHNFRNDGSRRYQRLLEDVIRTGNKTRVLMLSATPVNISLVDLRNQIYLMTEGQEQ

AFRDSLGVGNIRTLMARAQKAFKQWEQQQERRDKAQLLDQLGADFLRLLNGVSISRSRRQIEQFYAEEME

RIGPFPSRARPINASPHTDINGTLSYQDLAEQIGAFKLAVYQPSAYVVDQERLAELESRRQAQNFNQKDS

ERFLVGMMRVNALKRLESSAHALRLTMDRIIDKINKLLDKVERYGQGDESPTHGRIDEEIVPDADEEDEE

FVVNRNRSRNLYRLAELDLPRWTSDLKDDRAVLSAVRDRVAAITPERDGKLQDLKQRIRNKVNQPTRNRD

GKPNRKLLVFTTFKDTACYLYDNLKPLMQELGIAMAMVSGNETYATAGDNTFNAILTNFAPTARQRSATD

ANHDIDLLIATDCISEGQNLQDCDTVLNYDIHWNPVRLVQRFGRIDRIGSHNSSVQMVNYWPTNDMEIYL

RLQNRVQARMALADLTASGDEDPFSEEDMERDLRFRDAQLLKLRETIPDLDDCDDAPGLADFTLDDFLTQ

LLRYLERNKAALEAMPPGVYAVTVGDGIDTGGLV

**2.5 DNA Sequences for analysis of selective pressure**

>ma_h_sapiens lcl|NM_006164.4_cds_NP_006155.2_1 [gene=NFE2L2] [protein=nuclear factor erythroid 2-related factor 2 isoform 1] [protein_id=NP_006155.2] [location=556..2373]

ATGATGGACTTGGAGCTGCCGCCGCCGGGACTCCCGTCCCAGCAGGACATGGATTTGATTGACATACTTT

GGAGGCAAGATATAGATCTTGGAGTAAGTCGAGAAGTATTTGACTTCAGTCAGCGACGGAAAGAGTATGA

GCTGGAAAAACAGAAAAAACTTGAAAAGGAAAGACAAGAACAACTCCAAAAGGAGCAAGAGAAAGCCTTT

TTCGCTCAGTTACAACTAGATGAAGAGACAGGTGAATTTCTCCCAATTCAGCCAGCCCAGCACATCCAGT

CAGAAACCAGTGGATCTGCCAACTACTCCCAGGTTGCCCACATTCCCAAATCAGATGCTTTGTACTTTGA

TGACTGCATGCAGCTTTTGGCGCAGACATTCCCGTTTGTAGATGACAATGAGGTTTCTTCGGCTACGTTT

CAGTCACTTGTTCCTGATATTCCCGGTCACATCGAGAGCCCAGTCTTCATTGCTACTAATCAGGCTCAGT

CACCTGAAACTTCTGTTGCTCAGGTAGCCCCTGTTGATTTAGACGGTATGCAACAGGACATTGAGCAAGT

TTGGGAGGAGCTATTATCCATTCCTGAGTTACAGTGTCTTAATATTGAAAATGACAAGCTGGTTGAGACT

ACCATGGTTCCAAGTCCAGAAGCCAAACTGACAGAAGTTGACAATTATCATTTTTACTCATCTATACCCT

CAATGGAAAAAGAAGTAGGTAACTGTAGTCCACATTTTCTTAATGCTTTTGAGGATTCCTTCAGCAGCAT

CCTCTCCACAGAAGACCCCAACCAGTTGACAGTGAACTCATTAAATTCAGATGCCACAGTCAACACAGAT

TTTGGTGATGAATTTTATTCTGCTTTCATAGCTGAGCCCAGTATCAGCAACAGCATGCCCTCACCTGCTA

CTTTAAGCCATTCACTCTCTGAACTTCTAAATGGGCCCATTGATGTTTCTGATCTATCACTTTGCAAAGC

TTTCAACCAAAACCACCCTGAAAGCACAGCAGAATTCAATGATTCTGACTCCGGCATTTCACTAAACACA

AGTCCCAGTGTGGCATCACCAGAACACTCAGTGGAATCTTCCAGCTATGGAGACACACTACTTGGCCTCA

GTGATTCTGAAGTGGAAGAGCTAGATAGTGCCCCTGGAAGTGTCAAACAGAATGGTCCTAAAACACCAGT

ACATTCTTCTGGGGATATGGTACAACCCTTGTCACCATCTCAGGGGCAGAGCACTCACGTGCATGATGCC

CAATGTGAGAACACACCAGAGAAAGAATTGCCTGTAAGTCCTGGTCATCGGAAAACCCCATTCACAAAAG

ACAAACATTCAAGCCGCTTGGAGGCTCATCTCACAAGAGATGAACTTAGGGCAAAAGCTCTCCATATCCC

ATTCCCTGTAGAAAAAATCATTAACCTCCCTGTTGTTGACTTCAACGAAATGATGTCCAAAGAGCAGTTC

AATGAAGCTCAACTTGCATTAATTCGGGATATACGTAGGAGGGGTAAGAATAAAGTGGCTGCTCAGAATT

GCAGAAAAAGAAAACTGGAAAATATAGTAGAACTAGAGCAAGATTTAGATCATTTGAAAGATGAAAAAGA

AAAATTGCTCAAAGAAAAAGGAGAAAATGACAAAAGCCTTCACCTACTGAAAAAACAACTCAGCACCTTA

TATCTCGAAGTTTTCAGCATGCTACGTGATGAAGATGGAAAACCTTATTCTCCTAGTGAATACTCCCTGC

AGCAAACAAGAGATGGCAATGTTTTCCTTGTTCCCAAAAGTAAGAAGCCAGATGTTAAGAAAAACTAG

>ma_m_musculus lcl|NM_010902.3_cds_NP_035032.1_1 [gene=Nfe2l2] [protein=nuclear factor erythroid 2-related factor 2] [protein_id=NP_035032.1] [location=234..2027]

ATGATGGACTTGGAGTTGCCACCGCCAGGACTACAGTCCCAGCAGGACATGGATTTGATTGACATCCTTT

GGAGGCAAGACATAGATCTTGGAGTAAGTCGAGAAGTGTTTGACTTTAGTCAGCGACAGAAGGACTATGA

GCTGGAAAAACAGAAAAAACTCGAAAAGGAAAGACAAGAGCAACTCCAGAAGGAACAGGAGAAGGCCTTT

TTTGCTCAGTTTCAACTGGATGAAGAAACAGGAGAATTCCTCCCAATTCAGCCGGCCCAGCACATCCAGA

CAGACACCAGTGGATCCGCCAGCTACTCCCAGGTTGCCCACATTCCCAAACAAGATGCCTTGTACTTTGA

AGACTGTATGCAGCTTTTGGCAGAGACATTCCCATTTGTAGATGACCATGAGTCGCTTGCCCTGGATATC

CCCAGCCACGCTGAAAGTTCAGTCTTCACTGCCCCTCATCAGGCCCAGTCCCTCAATAGCTCTCTGGAGG

CAGCCATGACTGATTTAAGCAGCATAGAGCAGGACATGGAGCAAGTTTGGCAGGAGCTATTTTCCATTCC

CGAATTACAGTGTCTTAATACCGAAAACAAGCAGCTGGCTGATACTACCGCTGTTCCCAGCCCAGAAGCC

ACACTGACAGAAATGGACAGCAATTACCATTTTTACTCATCGATCTCCTCGCTGGAAAAAGAAGTGGGCA

ACTGTGGTCCACATTTCCTTCATGGTTTTGAGGATTCTTTCAGCAGCATCCTCTCCACTGATGATGCCAG

CCAGCTGACCTCCTTAGACTCAAATCCCACCTTAAACACAGATTTTGGCGATGAATTTTATTCTGCTTTC

ATAGCAGAGCCCAGTGACGGTGGCAGCATGCCTTCCTCCGCTGCCATCAGTCAGTCACTCTCTGAACTCC

TGGACGGGACTATTGAAGGCTGTGACCTGTCACTGTGTAAAGCTTTCAACCCGAAGCACGCTGAAGGCAC

AATGGAATTCAATGACTCTGACTCTGGCATTTCACTGAACACGAGTCCCAGCCGAGCGTCCCCAGAGCAC

TCCGTGGAGTCTTCCATTTACGGAGACCCACCGCCTGGGTTCAGTGACTCGGAAATGGAGGAGCTAGATA

GTGCCCCTGGAAGTGTCAAACAGAACGGCCCTAAAGCACAGCCAGCACATTCTCCTGGAGACACAGTACA

GCCTCTGTCACCAGCTCAAGGGCACAGTGCTCCTATGCGTGAATCCCAATGTGAAAATACAACAAAAAAA

GAAGTTCCCGTGAGTCCTGGTCATCAAAAAGCCCCATTCACAAAAGACAAACATTCAAGCCGCTTAGAGG

CTCATCTCACACGAGATGAGCTTAGGGCAAAAGCTCTCCATATTCCATTCCCTGTCGAAAAAATCATTAA

CCTCCCTGTTGATGACTTCAATGAAATGATGTCCAAGGAGCAATTCAATGAAGCTCAGCTCGCATTGATC

CGAGATATACGCAGGAGAGGTAAGAATAAAGTCGCCGCCCAGAACTGTAGGAAAAGGAAGCTGGAGAACA

TTGTCGAGCTGGAGCAAGACTTGGGCCACTTAAAAGACGAGAGAGAAAAACTACTCAGAGAAAAGGGAGA

AAACGACAGAAACCTCCATCTACTGAAAAGGCGGCTCAGCACCTTGTATCTTGAAGTCTTCAGCATGTTA

CGTGATGAGGATGGAAAGCCTTACTCTCCCAGTGAATACTCTCTGCAGCAAACCAGAGATGGCAATGTGT

TCCTTGTTCCCAAAAGCAAGAAGCCAGATACAAAGAAAAACTAG

>ma_p_troglodytas lcl|XM_001145876.3_cds_XP_001145876.2_1 [gene=NFE2L2] [protein=nuclear factor erythroid 2-related factor 2 isoform X1] [protein_id=XP_001145876.2] [location=553..2370]

ATGATGGACTTGGAGCTGCCGTCGCCGGGACTCCCGTCCCAGCAGGACATGGATTTGATTGACATACTTT

GGAGGCAAGATATAGATCTTGGAGTAAGTCGAGAAGTATTTGACTTCAGTCAGCGACGGAAAGAGTATGA

GCTGGAAAAACAGAAAAAACTTGAAAAGGAAAGACAAGAACAACTCCAAAAGGAGCAAGAGAAAGCCTTT

TTCGCTCAGTTACAACTAGATGAAGAGACAGGTGAATTTCTCCCAATTCAGCCAGCCCAGCACATCCAGT

CAGAAACCAGTGGATCTGCCAACTACTCCCAGGTTGCCCACATTCCCAAATCAGATGCTTTGTACTTTGA

TGACTGCATGCAGCTTTTGGCGCAGACATTCCCGTTTGTAGATGACAATGAGGTTTCTTCGGCTACGTTT

CAGTCACTTGTTCCTGATATTCCCGGTCACATCGAGAGCCCAGTCTTCATTGCTACTAATCAGGCTCAGT

CACCTGAAACTTCTGTTGCTCAGGTAGCCCCTGTTGATTTAGACGGTATGCAACAGGACATTGAGCAAGT

TTGGGAGGAGCTATTATCCATTCCTGAGTTACAGTGTCTTAATATTGAAAATGACAAGCTGGTTGAGACT

ACCATGGTTCCAAGTCCAGAAGCCAAACTGACAGAAGTTGACAATTATCATTTTTACTCATCTATACCCT

CAATGGAAAAAGAAGTAGGTAACTGTAGTCCACATTTTCTTAATGCTTTTGAGGATTCCTTCAGCAGCAT

CCTCTCCACAGAAGACCCCAACCAGTTGACAGTGAACTCATTAAATTCAGATGCCACAGTCAACACAGAT

TTTGGTGATGAATTTTATTCTGCTTTCATAGCTGAGCCCAGTATCAGCAACAGCATGCCCTCACCTGCTA

CTTTAAGCCATTCACTCTCTGAACTTCTAAATGGGCCCATTGATGTTTCTGATCTATCACTTTGCAAAGC

TTTCAACCAAAACCACCCTGAAAGCACAGCAGAATTCAATGATTCTGACTCCGGCATTTCACTAAACACA

AGTCCCAGTGTGGCATCACCAGAACACTCAGTGGAATCTTCCAGCTATGGAGACACACTACTTGGCCTCA

GTGATTCTGAAGTGGAAGAGCTAGATAGTGCCCCTGGAAGTGTCAAACAGAATGGTCCTAAAACACCAGT

ACATTCTTCTGGGGATATGGTACAACCCTTGTCACCATCTCAGGGGCAGAGCACTCACGTGCATGATGCC

CAATGTGAGAACACACCAGAGAAAGAATTGCCTGTAAGTCCTGGTCATCGGAAAACCCCATTCACAAAAG

ACAAACATTCAAGCCGCTTGGAGGCTCATCTCACAAGAGATGAACTTAGGGCAAAAGCTCTCCATATCCC

ATTCCCTGTAGAAAAAATCATTAACCTCCCTGTTGTTGACTTCAACGAAATGATGTCCAAAGAGCAGTTC

AATGAAGCTCAACTTGCATTAATTCGGGATATACGTAGGAGGGGTAAGAATAAAGTGGCTGCTCAGAATT

GCAGAAAAAGAAAACTGGAAAATATAGTAGAACTAGAGCAAGATTTAGATCATTTGAAAGATGAAAAAGA

AAAATTGCTCAAAGAAAAAGGAGAAAATGACAAAAGCCTTCACCTACTGAAAAAACAACTCAGCACCTTA

TATCTCGAAGTTTTCAGCATGCTACGTGATGAAGATGGAAAACCTTATTCTCCTAGTGAATACTCCCTGC

AGCAAACAAGAGATGGCAATGTTTTCCTTGTTCCCAAAAGTAAGAAGCCAGATGTTAAGAAAAACTAG

>ma_o_anatinus lcl|XM_007671274.1_cds_XP_007669464.1_1 [gene=NFE2L2] [protein=nuclear factor erythroid 2-related factor 2 isoform X1] [protein_id=XP_007669464.1] [location=23..1777]

ATGTTAATTCTATCGGGGTGGTGTCATTTGTCCCCCACCCCCCGTGCAAAGGACATGAACTTGATTGACA

TACTCTGGAGACAGGACATCGACCTCGGGGCGGGCCGGGAAGTGTTTGACTTCTGCCAGCGGCAGAAGGA

GTATGAGCTGGAGAAACAGAAGAAATTGGAAAAGGAAAGGCAAGAGCAGCTGCAGAAAGAGCGGGAGCAG

GCCTTGCTAGCCCAGTTCCAGCTGGACGAGGAGACGGGCGAGTTCCTCCCCATCCAGCCCGCGCGGCCCT

CTCAACTTGAAGGCGGGGATGGGCCCGCGGCCTTCTCCCAGAGCCCCCCAACCCCCAAGCCGGATGCCTT

GACCTTCGATGACTGCATGCAGCTCTTGACCGAGACGTTCCCCTTCGTGGACGACAATGAGGTTGCTCCA

GCCACACTTCAATCTCTCAGTCCACCCCCTGCCGAGAGCAGCCCTGTCTTCGTCCCTCCCAGCCCGACTC

CAGCTCCCGCCGAAGCCCCTGTCCTGGAGCCGGCCGCCACGGACTCAGCCGCTATGCAAGACATAGAGCA

AGTGTGGGAGGAGCTGCTGTCCATTCCAGAGTTACAGTGTCTTAACATTCAAAATGACAAGCAAGCTGAG

GCGGCCCCACTGCCGAGTCCCGAACCCAAATCGGGCGCCGCAGACCGCCCCTATGGCTTCTACGACGTGC

TCTCCCCGCTGGCCTGCACCATTGAGAAAGAGATGAGCGACAGCAGCCCCGCCTTCCTTGGCGCTTTTGA

GGGCAGTGCCCTTCCCACGCAAGACCTCAGTGTATCAGGCGCCTGCGCCCAGCCCCCCAGCCCCTCCCTT

GGCCCTGACTTCTGTGAGGATTTCTACACCACATTTGTGGTGGAGCTGGAGCCGGGGGCAGAGGGGGCGG

GGGCTCCGAGCAGGTTGCTCACGGACCTGCTGAACGAGCCTGTGGACCTGGCTGACTTGGCCCTGTGCAA

AGCCTTCGCTACCCCCCGGCCCTGTGGCCGGCCCGAATCCAACGACGCCGACTCAGGCATCTCCCTCAAC

ACGAGCCCTGCTGCAGCCTCCCCCGAGCCTTTGGCTGACTCCGTCGACGGGGATGCTGCCCCAGGCTCCA

GTGACTCGGAGACGGACGACGTGGACAGCGGCCCGCCCGGTGGCGCCAAGATCCGGCCGCACGCGGGGGC

CGAGGGCGGGAGACAGGCAGACCCACCCAAGAAGGAGGTGCTGGCCGGCCGGGGGCCGCCCCCAGGCACC

AGGGACAGGCCCGCGGGCCGGCTGGAGGCGCATTTCACGCGGGATGAGCAGAGGGCCAAGGCTCTGCAGA

TCCCCTTCCCTGTGGAAAAGATCATCAACCTGCCCGTGGATGACTTCAATGAGATGATGTCCAAGGAGCA

GTTCAGCGAAGCCCAGCTGGCGCTCATCCGTGACATCCGCCGGAGGGGCAAGAACAAGGTGGCCGCCCAG

AACTGCCGCAAACGCAAGCTGGAAAACATCGTGGAACTGGAGCAGGACCTGGATCACCTGAAGGACGAGA

AGGAGAAGTTGCTCAAGGAGAAGGGAGAGCACGACCTTAGCCTCCGCCTCCTGAAGCAGCAGCTGAGCAG

CCTGTACCTGGAGGTCTTCAGCATGCTGCGTGACCAGGACGGGCAGCCCTACTCGCCGGCCGACTACTCC

CTGCAGCAGACGCGCGACGGCCACGTGTTCCTCGTCCCCAAGAGCAAGAAGCCGGGTGGACAACACGGAA

ACTAG

>ma_e_edwardii lcl|XM_006878800.1_cds_XP_006878862.1_1 [gene=NFE2L2] [protein=nuclear factor erythroid 2-related factor 2 isoform X1] [protein_id=XP_006878862.1] [location=102..1925]

ATGATGGACTTGGAGCTGCCGTCACCGGAACTGCCGTCCCAGCAGGACATGGATTTGATTGACATACTTT

GGAGGCAAGATATAGATCTTGGGGTAAGTCGTGAAGTATTTGATTTCAGTCAGCGACGCAAGGAGTATGA

GCTGGAAAAGCAGAAAAAACTTGAAAAGGAAAGACAAGAACAACTCCAAAAGGAGCAAGAGAAAGCATTT

TTTGCTCAGCTTCAACTAGACGAAGAGACAGGTGAATTCCTCCCAATCCAGCCAGCCCAGCACATCCAAT

CAGAAACCAGTGGATCTGCCGACTACTCCCAGGGTGCACACATTCCCAAACCAGATGCTTTGTACTTTGA

TGACTGCATGCAGCTTTTGGCGGAGACATTCCCATTTGTAGATAACAATGAGGTTTCTTCAGCCACGTTT

CAGTCACTTGTTCCAGATATTTCCAGCCACATTGAGAGCCCAGTCTTCATTGCTCCTAGTCAGACTCAGA

CACCTGAAACTCCTGTCCTTCAGACAACTCCTGAACATCTAGACAATATGATGCAGGACGTTGATCAAGT

GTGGGAAGAGCTGTTATCCATTCCAGAATTACAGTGTCTTAATATTCAAAATGACAAGCTAGTTGAGACT

AACACTGTGGCAAGTACGGAAACAAAACTGACAGAAATTGACAACAGTTACCATTTCTACTCATCTATAC

CCTCACTGGAAAAAGAAGTAGGTGACTGCAGTTCAAATTTTCTCAATGCCTTTGAGGATTTCTTTGACAA

TATTCTACCTACAGATGACTCAAACCAGTTGACAGTGAACTCATTAAATGCAAATGCTACAATAAACACC

GATTTTGGTGATGAGTTTTATTCTGCTTTCATAGCAGAGCCCAGTGTCAGCAACAGCATATCTTCATCTG

CGGCATTAAGCCAGCCGCTGACAGAACTTCTGAATGGGTCTATTGATATTTCTGATCTATCACTTTGTAA

AGCTTTCAACCAAAGCCACCCTGAAAGCACAGCAGAATTCAATGACTCTGACTCTGGCATTTCACTGAAC

ACAAGCCCTAGCATGGCATCGCCAGAGCACTCAGTGGAGTCTTCCATCTACGGAGACACACCGCTTGGCT

TCAGTGACTCAGAAATGGAAGAGAGGGATAGTACTCCTGAAAGCGTCAAACTGAATGGTCCTAAAACACA

GCCAGTACAGTCTTCTGAAGATACAGCCCAACCCCTGTCACCATCTCCAGGGCACAGTGCCTCTGGGGGT

GATGCCCTGTGTGAAAACACCCCAAAAAACGAGTTGCCTGTAAGTCCTGGTCATCGAAAAACCCCATTCA

CGAAAGACAAACATTCAAGCCGCTTGGAATCTCATCTCACAAGAGATGAGCTAAGGGCCAAAGCTCTCCA

TATTCCATTCCCTGTAGAAAAAATCATCAACCTTCCTGTTGACGACTTCAATGAAATGATGTCCAAGGAG

CAATTCAGTGAAGCTCAAGTTGCATTAATCCGAGATATACGTCGAAGGGGTAAGAATAAAGTGGCTGCTC

AGAACTGCAGAAAAAGAAAACTGGAAAATATTGTAGAACTAGAGCAGGATTTGGATCATTTAAAAGATGA

AAAAGAAAAATTGCTCAGAGAAAAAGGAGAAAATGACAAAAGCCTCCACCTCCTGAAAAAACAGCTCAGC

ACCTTATACCTTGAAGTCTTCAGCATGCTGCGTGATGAAGATGGAAAACCCTATTCTCCTAGTGAATACT

CCTTGCAGCAAACTAGAGATGGCAATGTATTCCTTGTTCCCAAAAGTAAGAAGCCAGATGTTAAGAAAAA

>ma_f_catus lcl|XM_003990893.3_cds_XP_003990942.1_1 [gene=NFE2L2] [protein=nuclear factor erythroid 2-related factor 2 isoform X1] [protein_id=XP_003990942.1] [location=152..1972]

ATGATGGACTTGGAGCTGCCGCCGCCCGGACTGCCGTCCCAGCAGGACATGGATTTGATTGACATACTTT

GGAGGCAAGATATAGATCTCGGGGTAAGTCGAGAGGTATTTGACTTCAGTCAACGACGGAAGGAACATGA

GCTGGAAAAACAGAAAAAACTTGAAAAGGAAAGACAAGAACAACTCCAAAAGGAGCAAGAGAAAGCCTTC

TTTGCTCAGTTACAACTAGATGAAGAGACAGGTGAATTCCTCCCAATTCAGCCTGCCCAACACATCCCAT

CAGAAACCAGTGGATCTGCCAACTACTCCCAGGTTGCCCACATTCCCAAACCAGATGCTCTGTACTTCGA

TGACTGCATGCAGCTTTTGGCAGAGACATTCCCATTTGTAGATGACAATGAGGTTTCTTCAGCTGCGTTT

CAGTCACTTGTTCCTGATATTCCCAGCCAAATCGAGAACCCCGTCTTCATTGCTCCTAATCAGGCTCAGT

CACCTCAGACTCTTGTCACTCAGTCAGTCATTGCTGATTTAGACAATATGCAGCAGGACATTGAGCAAGT

TTGGGAGGAGCTACTGTCCATTCCAGAATTACAGTGTCTTAATATTCAAAATGACAAGTTGGTTGAGACT

AGCACGGTCCCAAGTCCAGAAACCAAAATGACAGAAATTGACAACAATTATCATTTCTACTCATCGATGC

CCTCACTGGAAAAGGAAGTAGGTAACTGCAGTCCACATTTTCTCAGTGCTTTTGAGGATTCCTTCAGCAG

CATCCTCTCCACAGAAGATTCCAGCCAGTTGACCGTGAACTCATTAAATTCAGATGCCACAATAAACACT

GATTTTGGTGATGAATTTTATTCTGCTTTCATAGCAGAACCTAGTAGCAGCAATAGCATGCCCTCCTCGG

CTACTTTAAGCCAGTCACTCTCTGAACTTCTTAATGGGCCCATTGATGTTTCTGATCTATCACTTTGTAA

AGCCTTCAACCAAAACCACCCTGAAAGCACAGAATTCAATGACTCTGACTCTGGCATTTCGCTGAACACG

AGTCCTGGCCTGGCATCACCAGAACACTCAGTGGAATCTTCTGTCTATGGAGACACACCGCTTGGCTTCA

GTGATTCTGAAATGGAAGAGATAGATAGTGCCCCTGGGAGTGTCAAACAGAATGGTCCTAAAACACAACC

CGTACAGTCTTCTGGAGATACAGTCCAACCCCTGTCACCATCCCCAGGGCACAGTGCTCCAGTGTGTGAT

GCCCAGTGTGAAAACACGCCCAAGAAAGAATTGCCTGTAAGTCCCGGTCATCGAAAAACCCCATTCACAA

AAGACAAACATTCAAGCCGCTTGGAGGCTCATCTCACAAGAGATGAGCTAAGGGCCAAAGCTCTCCACAT

CCCATTCCCTGTTGAAAAGATCATTAACCTCCCTGTTGATGACTTCAATGAGATGATGTCCAAGGAACAA

TTCAACGAAGCTCAACTGGCATTAATTCGAGATATACGCAGGCGGGGTAAGAATAAAGTGGCTGCTCAGA

ACTGCAGAAAAAGAAAACTGGAAAATATAGTGGAACTGGAACAAGATTTGGATCATTTGAAAGATGAAAA

AGAAAAATTGCTCAGAGAAAAGGGCGAGAATGACAAAAGCCTGCATCTACTGAAAAAACAGCTTAGCACC

CTGTATCTTGAAGTCTTCAGCATGTTACGTGATGAAGATGGAAAACCTTACTCTCCTAGTGAATACTCCC

TGCAGCAAACAAGAGATGGCAATGTGTTCCTTGTGCCCAAGAGTAAGAAGCCAGATGTGAAGAAAAACTA

G

>ma_m_brandtii lcl|XM_005857615.1_cds_XP_005857677.1_1 [gene=NFE2L2] [protein=nuclear factor erythroid 2-related factor 2 isoform X5] [protein_id=XP_005857677.1] [location=17..1780]

ATGGATTTGATTGACATACTCTGGAGGCAAGATATAGATCTTGGGGTGAGTCGAGAAGTATTTGACTTCA

GTCAACGACGGAAGGAGCATGAGCTGGAAAAACAGAAAAAACTTGAAAAGGAAAGACAAGAACAACTCGA

AAAGGAGCAAGAGAAAGCCTTTTTCGCTCAGTTACAACTAGACGAAGAGACAGGTGAATTCCTCCCAATT

CAGCCAGCTCAACATATCCCATCAGAAACCAGTGGATCTGCCAACTACTCCCAGGTTGCCCACATTCCCA

AACCAGATGCTTTGTACTTCGATAACTGCATGCAGCTTTTGGCAGAGACATTCCCGTTTGTAGAGGACAA

TGAGGTTTCTTCGCCTACGTTTCAATCACTTGTTCCTGATGTTCCCAGCCACATCGAGAGCCCAGTCTTT

ACTGCGCCTAGTCAGACTCAGTCATCTGAACCTGTTGTCCTTCAGCTAATCAGTGATTTAGGTAATATGC

AGCAGGACATTGAGCAAGTTTGGGAGGAACTACTATCCATTCCAGAATTACAGTGTCTTAATATTCAAAA

TGACAAGCTGGTTGAGACTAACACGGTTCCAAGTCCAGAAACCAAACAGGCAGACATTGACAACAGTTAT

CATTTCTACTCATCTATCCCTACACTGGAAAAAGAAGTAGGTAACTGCAGTCCACCTTTTCTCAATGCTT

TTGAGGATTCCTTCAGCAGCATCCTCACCACAGAAGACCCCAGCCAACTGACAGTGAACTCATTAAATTC

AAATGCCACCATAAACACAGATTTTGGTGATGAATTTTATTCTGCTTTCGTAGAGGAGCCCAGTATCAAC

AACAGCATGTCTTCCTCAGCTACTTTCAGCCAGTCACTCTCTGAACTTCTCTATGGGCCCATTGATGTTT

CTGATCTATCACTTTGTAAAGCCTTCAATCCTGAAAGCACAGCAGAATTCAATGATTCTGACTCTGGCAT

TTCACCGAACACAAGCCCCAGCATGGCATCACCAGAACACTCAGTGGAATCTTCTGGCTATGGAGACACA

CCACTTGGCTTTAGTGATTCTGAAATGGAAGAGACAGACAGTGCCGCTGGCAGTGTCAAACACAGTGGTC

CTAAAACACAGCCAGTTCAGACTTCTGGGGAGACAGTTCACCCCCCGTCACCATCTCGGGGGCACAGTGC

CCCAGTAAGTGATGCCCAGTGTGAAAACACACAAAAGAAAGAATTGCCTGTAAGTCCTGGTCATCGCAAA

ACCCCATTCACAAAAGATAAACATTCAAGCCGCTTGGAGGCTCATCTCACAAGAGATGAGCTAAGGGCGA

AAGCTCTCCATATCCCATTCCCTGTAGAAAAAATAATTAACCTCCCTGTTGATGACTTCAATGAAATGAT

GTCCAAGGAACAATTCAATGAGGCTCAACTTGCATTAATTCGAGATATACGTAGGAGGGGTAAGAATAAA

GTGGCTGCTCAGAATTGCAGAAAAAGAAAACTGGAAAATATAGTGGAACTGGAACAAGATTTGGATCATT

TAAAAGATGAAAAAGAAAAATTGCTCAAAGAAAAAGGAGAAAATGACAGAAACCTCCATCTACTGAAAAA

ACAACTCAGCACCTTATATCTAGAAGTCTTCAGCATGCTCCGAGATGAAGACGGAAAACCTTACTCTCCT

AGTGAATACTCCCTGCAGCAGACGAGAGATGGCAACGTATTCCTTGTTCCCAAGAGTAAGAGGCCAGATG

TTAAGAAAACCTAG

>ma_m_domestica lcl|XM_007494424.1_cds_XP_007494486.1_1 [gene=NFE2L2] [protein=nuclear factor erythroid 2-related factor 2 isoform X2] [protein_id=XP_007494486.1] [location=147..1940]

ATGATGGACTTGGAACTGCCCCCGTCGCAACAGGACATGAATTTAATTGACATCCTCTGGAGACAAGATA

TAGATCTTGGAGCAAGACGAGAAGTGTTTGACTTCAGTCAAAGGCGGAAAGAGCATGAGCTGGAAAAACA

AAAGAAACTTGAAAAAGAAAGACAAGAACAACTCCAGAAAGAACAAGAGAAAGCCTTTCTGGCTCAACTG

CAGCTAGACGAAGAAACAGGTGAATTCCTCCCAATTCAGCCAGCCCAGCACATTGAGCCTAGCACATCTG

CCAGCTACTCACAAGCTGCTGACATCCCCAAAGCAGATGCTCTGTTCTTTGATGACTGCATGCAGCTTTT

GGCAGAGACCTTCCCATTTGTAGAAGATAATGAGGTTTCTTCAGCCACATTTCAGTCACTTGTTCCAGAT

CACATTGACAGCAACCCAGTCTTCATTACTTCCAGTCAAGCTCAGCTCCCTGAATCGTCTGTCCTTCAGT

CTATAGTAGAAAACAACATGCAGGATATCGAGCAAGTGTGGGAGGAGCTGTTGTCTATTCCAGAGTTACA

GTGTCTTAATATTGAAAATGACAAGTTGGCTGAGGCTACCATAGTTCCAAGTCCTGAAGCAAAACCGACA

GAAATCAATGACAGTTACAATTTCTACACTTCCCTCTCCACGATGGAAAAAGAAGTAGCTACCTGCAATC

CAGATTTCCTCAGTGCTTTTGAGGACTCCTTTGGCAACATCCTTCCCACAGAAGACCCTAACCAGTTGAG

AATGAACTCTTTAAATTCAAATGCCACAATAAACACTGATTTTTGCGAGGAATTTTACTCAACTTTCATA

GCAGAAACAAACATAAACAACAGTATGCCTTCCCCCGCCCACATCAGCCAGTCACTTTCAGAACTCTTAA

ATGAGCCCATTGATATTTCTGACCTTTCACTCTGTAAGGCCTTTAACAGCAACCCTCCTGAAAACCCCCC

AGAATGTAATGATTCTGACTCAGGCATTTCTTTGAACACTAGCTCTAACATGGCATCACCAGAACATTCA

GTGGAGTCATCCCTCTATGGAGACACGCCACTGGGCTTCAGTGATTCTGAAATGGAAGATGTTGACAGTG

CTCCTGGAAGCACACAGCAGAGCGGAGCCAGGATGCAGCCAGTGCCATTTCAGGAGGACATGCCCTATCC

AGTGTCTCCCACTCAGGGGCCAACCGTTCCTGCGCCTGATGCTCTGCAGAGTGTAAGCACGCCAAAGAGA

GAGTCACCCACCAGTCCTGGTCACCAGAAAGTCCCATTTACAAAAGACAAACATTCAGGCCGCTTAGAAT

CCCATTTCACGAGAGATGAGATGAGAGCAAAGGCTCTTCATATCCCTTTCCCTGTAGAAAAGATTATTAA

CCTTCCTGTGGATGACTTCAATGAAATGATGTCAAAAGAGCAGTTTAATGAGGCCCAACTTGCACTTATT

CGAGATATTCGTAGGCGGGGCAAAAACAAGGTGGCTGCTCAGAACTGCAGGAAAAGAAAACTGGAAAACA

TAGTGGAACTGGAGCAAGATTTGGATCATTTAAAAGATGAAAAGGAAAAGCTGCTCAGAGAAAGAGGAGA

AAATGACAAAAGCCTCCATCTACTGAAAAAACAGCTCAGCACCTTGTATCTTGAGGTGTTCAGCATGCTA

CGAGATGAAAATGGAGAGCCCTACTCCCCTAGTGAGTATTCCCTGCAGCAAACAAGGGATGGTAACGTGT

TCCTCGTTCCCAAAAGCAAGAAACCTGACATTAAGAGAAATTAG

>ma_o_afer lcl|XM_007938309.1_cds_XP_007936500.1_1 [gene=NFE2L2] [protein=nuclear factor erythroid 2-related factor 2 isoform X1] [protein_id=XP_007936500.1] [location=111..1919]

ATGATGGACCTGGAGCTGCCGTCACCCGGACTGCCGTCCCAGCAGGAAATGGATTTGATTGACATACTTT

GGAGGCAAGATATAGATCTTGGGGTAAGTCGTGAAGTATTTGACTTCAGTCAGCGGCGCAAGGAGTATGA

GCTGGAAAAACAGAAAAAACTTGAAAAGGAAAGACAAGAACAACTCCAAAAGGAGCAAGAGAAAGCCTTT

TTCGCTCAGCTACAACTAGATGAAGAGACAGGTGAAATCCTCCCAATTCAGCCAGCCCAACACATCCATT

CGGAAACCAGTGGATCTGCCAACTACTCTCAGGTTGCTCACATTCCCAAACTAGATGTTTTGTACTTCAC

TGACTGCATGCAGCTTTTGGCAGAGACATTCCCATTTGTAGAAGACAATGAGGTTTCCTCGGCTACATTT

CAATCGCTTGTTCCTGATATTCCCAGCCATATTGAGCCCCCCATCTTCATTGCTCCTGATCAGTCACCTG

AAACTCCTGTTCTTCAAACAACTGTTGCCCATTTAGACAATATGCAAGACGTTGATCAAGTTTGGGAGGA

GCTATTATCCATTCCAGAATTACAGTGTCTTAATATTCAAAATGACAAGCTAGTTGAGACTAGCACTGTT

CCAAGTCCAGAAACAAAACTGACAGAAATTGACAATTATCATTTCTACCCATCGATCCCCTCACTGGAAA

AAGAAGTAGGTGATTGCAGTCCACATTTGCTCAATGCTTTTGAGGATTTCTTTGGCAGCATCCTACCCAC

AGATGACCCTGGCCAGTTGACAGTGAACTCATTAAATTCAAATACAATAAACACCGATTTTGGTGATGAA

TTTTATTCTGCTTTCATAGCAGAGCCCAGCATCAACAACAGCATGTCCTCCTCTGCTACCTTAAGCCAAC

CACTTTCTGAACTTCTAAATGGGCCCATTGATGTTTCTGATCTATCACTTTGTAAAGCTTTCAATGAAAA

CCACCCTGAAAGCACAGCAGAATTCAATGATTCTGACTCTGGCATTTCATTGAACACAAGTCCCAGCAGG

GCTTCACCAGAACACTCAGTGGAGTCTTCCATCTATGGAGATACACCGCTTGGCTTCAGTGATTCTGAAA

TGGAAGAGAGAGATAGTACTCCTGAAAGTGTCCAACAGAATGGTCCTAAAACACAGCCAGTACAGTCTTC

TGGGGATATAGTCCAGCCCCTGTCACCATCTCCAGGGCACAGTGCTTCAGTGCATGATGCACAGTGTGAA

AATGCCCCCCAAAAAGAATTGCCTGTAAGTCCTGGTCATCGAAAAACCCCATTCACAAAAGACAAACATT

CAAACCGCTTGGAAGCACATCTCACAAGAGATGAGCTAAAGGCAAAAGCTCTCCGTATTCCATTCCCCGT

AGAAAAAATCATTAACCTCCCTGTTGACGACTTCAACGAAATGATGTCCAAGGAGCAATTCAATGAAGCT

CAAGTTGCATTAATTCGAGATATACGTAGGAGGGGTAAGAATAAGGTGGCTGCTCAGAATTGCAGAAAAA

GAAAACTGGAAAATATAGTAGAACTAGAACAAGATTTGGATCATTTAAAAGATGAAAAAGAAAAATTGCT

CAAAGAAAAAGGAGAAAATGACAAAAGCCTCCATCTACTGAAAAAACAACTCAGCACCTTGTACCTTGAA

GTCTTCAGCATGCTACGAGATGAAGCTGGACAACCGTATTCTCCTAGTGAATACTCCCTGCAGCAAACCA

GAGATGGCAATGTATTCCTTGTTCCCAAAAGTAAGAAGCCAGATGTTAAGAAAAACTAG

>ma_o_cuniculus lcl|XM_002712305.2_cds_XP_002712351.2_1 [gene=NFE2L2] [protein=nuclear factor erythroid 2-related factor 2 isoform X1] [protein_id=XP_002712351.2] [location=183..1985]

ATGATGGACTTGGAGCTACCGCCGTCGGGACTGCAGTCCCAGCAGGACATGGATTTGATTGACATACTTT

GGAGGCAAGATATAGATCTTGGGGTAAGTCGAGACGTATTTGACTTCAGTCAGCGACAGAAGGAGTATGA

GCTGGAAAAACAGAAACAACTTGAAAAGGAAAGACAAGAACAACTCCAAAAGGAGCAAGAGAAAGCCTTT

TTCGCTCAGTTACAACTAGATGAAGAGACAGGTGAAATCCTCCCAATTCAGCCAGCCCAACACATCCAGT

CAGAAACCAGTGGATCTGCCAACTACTCCCAGGTTGCCCACATTCCCAAACCAGATGCTCTGTACTTTGA

TGACTGCATGCAGCTTTTGGCAGAGACATTCCCATTTGTAGATGACAATGAGGTTTCTTCAGCTACGTTT

CAGTCACTTGTTCCTGATACTCCCAGCCACGTCGAGAGCCCAGTCTTCACTGCACCTAATCAGGCTCAGA

CACCTGAAACTTCTTTTGTTCAGGTAGCTGTTGCTGATTTAAACAATATGGAACAGAACATTGAGCAAAT

TTGGGAGGAATTATTATCCATTCCAGAATTACAGTGTCTTAATATTGAAAAGGACAAGCTGGTTGAGACT

ACCACGGTTCCAAGTGCAGAAGTCAAACTGACAGAAGTTGACAACAATTATCATTTCTACTCGTCGGCTC

CCTCACTGGAAAAAGAAGATAACTGCAGTGCGCATTTTCTTAGTGCTTTTGAGGATTCTTTCGGCAGCAT

CCTCTCCGCAGATGACCCCGCCCAGCTGAGCGTGAACTCAAATGCCACATTAAACACAGATTTTGGTGAT

GAATTTTATGCTGCCTTCATAGCTGAACCCAGTGTCAGCAACAGCATGTCCTCTGCTCCCATCAGCCAGT

CACTCTCTGAACTTCTAAATGGGCCTATTGATGTTTCTGACCTATCCCTTTGTAAAGCTTTTAACCAGAA

CCACCCTGAAAGCACAGAATTCGCCGACTCTGACTCTGGCATTTCACTGAACACAAGTCCCAGCATGGCA

TCACCAGAACACTCAGTGGAATCTTCTGTCTGTGGAGACACACCACTTGGCTTCAGTGATTCTGAAATGG

AAGAACTAGACAGTACCCATGGGGTTGTCAAACAGAATGCTTCTAAAACACAGCCAATACATTCTTCTGG

GGATACAGTACAACCCCTGTCACCATCTGGGGGGTACAGTGCTCCAGTGCACAATGCCCAATGTGAAAAC

ACACCAAAGAAGGAAACGCCTGGGAGTCCCAGTCCTCGAAAAACCCCATTCACAAAAGACAAACATTCAG

GCCGCTTGGAGGCCCATCTCACAAGAGATGAACTTAGGGCAAAAGCTCTCCATATCCCATTCCCTGTAGA

GAAAATCATTAACCTCCCTGTCGATGACTTCAATGAAATGATGTCCAAGGAGCAATTCAATGAAGCACAA

CTTGCATTAATTCGAGATATACGTAGGAGAGGTAAGAATAAAGTGGCTGCTCAGAATTGCAGAAAAAGGA

AACTGGAAAATATAGTAGAACTGGAGCAAGATTTAGATCACTTAAAAGATGAGAAAGAAAAATTGCTCAA

AGAAAAAGGAGAAAATGACAAAAGCCTCCACCTCCTTAAGAAGCAACTCAGCACCTTGTATCTGGAAGTC

TTCAGCATGTTACGGGATGAGCACGGGAAGCCGTACTCGCCTAGTGAGTACTCCCTGCAGCAGACGAGGG

ACGGCAATGTATTCCTTGTTCCCAAAAGTAAGAAACCAGATGTCAAACACTAG

>av_a_platyrhynchos lcl|NM_001310777.1_cds_NP_001297706.1_1 [gene=NFE2L2] [protein=nuclear factor, erythroid 2-like 2] [protein_id=NP_001297706.1] [location=25..1860]

ATGCAGCTCAGCTGGGTAAGATTTCCAGGAGCTTCAATACGAGAACATGGGAACTTTAGAGGCAGAGGCC

ATGGTGTCAAGGACATGAACTTGATTGACATCCTTTGGAGGCAAGATATAGACCTTGGGGCAAGGCGTGA

AGTTTTTGATTTTAGTCAACGACAGAAGGAGTATGAACTCGAGAAACAGAAGAAACTTGAAAAGGAAAGA

GAAGAGCAGCTCCAGAAGGAGCAGGAGAAAGCCTTGCTGGCTCAGCTGGAGTTAGACGAGGAGACAGGTG

AATTTGTTCCAGTTCAGCCAGCTCAGCGCATTCAGTCAGAAAACACTGAGCCACCAATCACTTTTTCACA

GAGCACGCATACTTCAAAACCAGAAGCAGAGGCCTTGTCCTTTGATGACTGCATGCAGCTCTTGGCAGAA

GCATTCCCGTTTATAGATGATAATGAGGCTTCTTCAGCTGCATTTCAGTCAATGGTTCCTGCTCAGATTG

ATAGTGACCCAGAGTTTATTTCCTCTAATCAAACTCAGCCACCTGAATCACCTGGTATAGTTCCACTTAC

TGATGCGGAGAATATGCAGAACATAGAGCAAGTCTGGGAAGAATTATTGTCCCTTCCAGAGTTACAGTGT

CTTAACATTGAAAATGATAACCTGGCTGAGGTAAGCACAATCACAAGCCCTGAAACCAAGTCAACAGAGA

TGCACAACGGCTATAATTACTACAACTCATTACCTATCATGAGAAAAGATGTTAACTGTGGTCCGGATTT

CCTGGAGACTGTTGAGAGCCCTTTTCCCAGCATTTTGCAAACAGAAGACAGCAGCCAGCTGGTGGTGAAC

TCTTTAAATAACACATCCACCTCAAACCCCGATTTTTGTGAGGATTTCTATACCACCTTTTTGTATTCAA

AGGGGGACAGTGACGTAGCAACGACAAACACTATCAGTCAATCACTTGCAGAAATTTTAAGTGAACCTAT

TGATCTTTCTGATTTCTCACTGTGGAGAGCTTTTAATGATGAACACTCAGGAACTGTACCAGAATGCAAT

GATTCTGACTCCGGTATTTCACTGAATGCAAATTCTAGGGTAGCATCACCTGAACACTCTGTTGAATCAT

CTGCCTGTGGAGATAAGACTTTTGGTTGTAGTGATTCTGAAATGGAAGATGTGGATAGTTCTCCTGGAAG

TGTGCCACAGAGCAATGCTAGTGTATACCCACTGCAATTCCAGGATCAAGTACTTTCTTCCGTGGAGCCA

AGCACTCGACCACCTAGCTTACAATGTACAAACACACCAAAGAAAGACCCTCCTGCTGGTCCAGGCCACC

CCAAAGCACCGTTCACAAAAGATAAGCCTTCAGGCCGCCTTGAAGCTCATCTCACAAGAGATGAGCAAAG

AGCAAAGGCTCTGCAGATCCCTTTTCCTGTAGAAAAAATCATCAATCTCCCTGTCGATGACTTCAATGAA

ATGATGTCTAAGGAGCAGTTCAGTGAAGCTCAGCCTGCTCTTATTCGAGATATACGCAGGAGAGGCAAGA

ATAAAGTGGCTGCTCAAAATTGCCGTAAAAGAAAACTGGAAAATATAGTGGAACTGGAGCAAGATTTGAG

TAACCTAAAAGATGAGAGAGAGAAGCTGCTTAAAGAAAAAGGGGAGAATGACAAAAGCCTTCGTCAAATG

AAAAAGCAACTAACCACCTTATACCTTGAAGTTTTCAGCATGCTACGTGATGAAGATGGAAAATCTTACT

CTCCTAGTGAATATTCACTGCAGCAAACTAGAGACGGCTATGTCTTTCTTGTTCCTAAAAGCAAGAAGTC

AGAGACTAAACTTTGA

>av_f_peregrinus lcl|XM_013298825.1_cds_XP_013154279.1_1 [gene=NFE2L2] [protein=nuclear factor erythroid 2-related factor 2] [protein_id=XP_013154279.1] [location=187..1929]

ATGAACTTGATTGACATCCTTTGGAGACAAGATATAGACCTTGGGGTGAGGCGTGAAGTTTTTGATTTTA

GTCAACGACAGAAGGAGTATGAACTTGAGAAACAGAAGAAACTTGAAAAGGAAAGACAAGAGCAGCTCCA

AAAAGAGCAGGAGAAAGCCTTGCTGGCTCAGCTGGAGTTAGACGAAGAAACAGGTGAATTTGTTCCTGTT

CAGCCAGCTCAGCGCATTCACTCAGAAAATACTGAGCCACCCATCGATTTTTCTCAGAGCACGCAGACTT

CAAAACCAGAAGCAGAGACCTTGTCCTTTGATGACTGCATGCAGCTCTTGGCAGAAGCATTCCCATTTAT

AGATGACGATGAGGTAAGAATGCTCAACGCAGTGGTTTCACGGGTCTGCAGCTCTCCGGAGTTCATTTCA

TCTGATCACGCTCAGCCACCGGAATCGCCTGGTTTAGTTTCACTTACTGATGCGGAGAATATGCAGAATA

TAGAGCAAGTTTGGGAAGAACTATTGTCCCTTCCAGAGTTACAGTGTCTTAACATTGAAAACGATAACCT

GGCCGAGGTAAGCACAATCACAAGCCCTGAAACCAAGCCAACAGAGATGCACAACAGATACAATTACTGC

AGCTCATTACCCATCATGAGAAAAGATGTTAACTGCAGTCCGGATTTCCTGGATAGCATGGAGGATCCCT

TTTCCAGCATTTTGCCACCAGAAGACACCAGCCAGCTGAGTGTGAACTCTTTAAAAGACACATCCCCTTC

AAACTCTGATTTCTGTGAGGATTTCTACGCCACCTTTATTGACACAAAGGCGAACGGTGACACAGCAACA

ACAAACACTATCAGTCAATCACTTGCAGAAATTCTAAGTGAACCTATTGATCTTTCTGATTTCTCACTGT

GTAAAGCTTTTAATGGCAACCACTCAGGAACTGTACCAGAATGTAATGATTCTGACTCTGGTATTTCATT

GAATGCGAGTTCCAGCGTAGCATCACCTGAACACTCTGTTGAATCATCTGCCTACGGAGATAAGGCTTTT

GGTTGTAGCGATTCTGAAATGGAAGACATGGATAGTGCTCCTGGAAATGTGCCGCAGAGCCATGCCAGCG

CGTACTCCTTGCAGCTCCAGGACCAAGTGTTTTCTTCCATGGGGCCGAGCGCTCGGACACCTAGTTTGCA

GTGTACAAATGCACCAAAGGAGGAACCCCCTGCTGGTCCTGGCCACCCCAAAGCTCCGTTCACAAAAGAT

AAACCTTCAGGCCGCCTTGAAGCTCATCTCACAAGAGATGAGCAAAGAGCAAAAGCTCTGCAGATCCCTT

TTCCTGTTGAAAAAATCATCAATCTCCCTGTTGATGACTTTAATGAAATGATGTCTAAGGAGCAGTTCAA

TGAAGCCCAGCTTGCGCTTATTCGAGATATTCGCAGGAGAGGCAAGAACAAAGTGGCTGCTCAAAATTGC

CGTAAAAGAAAACTGGAAAATATAGTGGAACTGGAGCAAGACTTGAGTAACTTAAAAGATGAGAAAGAGA

AACTGCTTAAGGAGAAAGGGGAGCATGACAAAAGTCTTCGTCAAATGAAAAAGCAACTGACTACCTTATA

CCTTGAGGTGTTCAGCATGCTACGTGATGAAGATGGAAAGTCTTACTCTCCTAGTGAATATTCACTGCAG

CAAACTAGAGATGGCAATGTCTTCCTTGTTTCTAAAAGCACCAAGTCAGAGACTAAACTTTGA

>av_a_chloris lcl|XM_009078783.1_cds_XP_009077031.1_1 [gene=NFE2L2] [protein=nuclear factor erythroid 2-related factor 2] [protein_id=XP_009077031.1] [location=1..1743]

ATGAACTTGATCGACATCCTTTGGAGGCAAGATATAGACCTCGGGGCAAGACGTGAAGTTTTTGATTTTA

GTCAACGACAGAAGGAGTATGAACTCGAGAAACAGAAGAAACTTGAAAAGGAAAGACAAGAGCAGCTCCA

AAAAGAGCAGGAGAAAGCCTTGCTGGCTCAGCTGGAGTTAGACGAAGAGACAGGTGAATTTGTTCCTGTG

CAGCCAGCTCAGAGCAGTCAGTCAGAAAATACTGAGCCACCAGTTGTTTTTTCACAGACCACAGAGCCTT

CAAAACCAGAAGCAGAGGCCTTGTCGTTTGAGGACTGCATGCAGCTCTTGGCAGAAGCATTCCCATTTGT

AGATGAGAATGAGGTTTCTTCAGATGCATTTCAGTCACTGGTTCCTGCTCAGATCAATAGCAACTCAGCC

TTCGTTTCCTCTGATCAAAGTCAGCCACCTGATCTAGTTCCACCTACTGAGACAGAGAATATGCAGAACA

TAGAGCAAGTTTGGGAAGAATTATTGTCCCTTCCAGAGTTACAGTGTCTTAACATCGAAAATGATAACCT

GGCTGAGGTAAGCACAATCACAAGCCCTGAAGCCAAACCAACAGAGATGCACAACAGATATAATTACTGC

AGCTCATTACCAACAATGAAAAAAGATGTTAACTGCAGTCCAGATTTCCTGGGTAGTATTGAGGGCCCCT

TCTCAGGCATTTTGCCATCAGAAGACACCAGCCATCTGAGTGTGAACTCTTTAAATGACACATCCCCTTC

AAACTCTGATTTCTGTGAGGAGTTCTATACCACCTTTATTGATACAAAGGCGAACGGTGACGCAGCAACG

ACAAACACTATCACTCAATCCCTCACAGAGATTCTAAGTGAACCTATTGATCTTTCTGACTTCTCACTGT

GTAAAGCTTTTAATGGCAATCACTCAGGGACTGTACCAGAATGTAATGATTCTGACTCTGGTATTTCATT

GAACGCAAGTTCTAGCGTAGCATCACCTGAACACTCTGTGGAATCATGTGCCTATGGAGATAAGACTTTG

GGTTGTAGTGATTCTGAAATGGAAGACGTGGATAGCGCTCCTGGAAGTGTGTCACAGAGCAATGCTAGTG

TGTACTCTTTGCAATTCCAGGATCCAGTGCTTTCTTCCATGGGGCCAAACACTCAAACACCAAGTTTACC

GTGTACAAACACAGTAAAGAAGGAACCCCCTGCTGCTCCTGGCCATCCCAAACCTCCCTTCACTAAAGAT

AAGTCTTCAAGCCGCCTTGAAGCTCATCTCACAAGAGATGAGCAAAGAGCGAAAGCTCTGCAGATCCCTT

TTCCTGTTGAAACAATCATCAATCTCCCTGTTGACGACTTCAATGAAATGATGTCTAAGGAGCAGTTCAA

TGAAGCGCAGCTCACGCTGATTCGCGACATACGCAGGAGAGGCAAGAACAAAGTGGCAGCTCAAAACTGC

CGTAAAAGGAAACTGGAAAACATAGTGGAACTGAAGCAAGACTTGAGTGACCTCAAAGATGAGAAAGAGA

AATTGCTTAAGGAAAAAGGAGAGCATGACAGAAGCCTTCGTCAAATGAAAAAGCAACTAACCACCTTATA

CCTCGAGGTCTTCAGCATGCTACGGGATGAGGATGGGAAGCCTTACTCTCCTAGCGATTATTCACTGCAG

CAAACTACAGATGGCAATGTCTTTCTTGTTCCTAAGAGCAAGAAGTCAGAGACTAAACTTTGA

>av_c_cristata lcl|XM_009698871.1_cds_XP_009697173.1_1 [gene=NFE2L2] [protein=nuclear factor erythroid 2-related factor 2 isoform X2] [protein_id=XP_009697173.1] [location=1..1746]

ATGAACTTGATTGACATCCTTTGGAGGCAAGATATAGACCTTGGGGCAAGGCGTGAAGTTTTTGATTTTA

GTCAACGACAAAAGGAGTATGAACTCGAGAAACAGAAGAAACTTGAAAAGGAAAGACAAGAGCAGCTCCA

GAAAGAGCAGGAGAAAGCCTTGCTGGCTCAGCTGGAGTTAGACGAAGAGACAGGTGAATTTGTTCCTGTT

CAGCCAGCTCAGCACATCCAGTCAGAAAATACTGAGCCACCGATTGTTTTTTCACAGACTTCAAAACCAG

AAGCAGAGGCCTTGTCCTTTGATGACTGCATGCAGCTCTTGGCAGAAGCATTCCCATTTATAGATGACAA

TGAGGCTTCTTCACCTGCATTTCAGTCACTGGTTCTTGCTCAGATCAATAGCAACCCAGTCTTCATTTCC

TCTGATCAAACTCAGCCACCTGAATCACCTGTTCTAGATCCACTTACTGATGCAGAGAATATGCAGAACA

TAGAGCAAGTTTGGGAAGAATTATTGTCCCTTCCAGAGTTACAGTGTCTTAACATTGAAAATGATAACCT

GGCTGAGATAAGCACAATCGCAAGCCCTGAAACCAAGCCAACAGAGATGCACAACAGCTATAATTACTAC

AGCTCATTACCTATCATGAGAAAAGATGTCAACTGCAGTCCAGATTTCCTGGATAGCATCGAGGGCCCCT

TTTCCAGCATTTTGCCACCAGAAGACACCAGCCAGCTGAGTGTGAATTCTTTAAATGATGCATCCCCTTC

AAACTCTGATTTCTGTGAGGATTTCTACACCACCTTTATTGATACAAAGGTGAATGTTGACATGGTAATG

ACAAACACTATCAGTCAATCATCACTTGCGGACATTCTAAGTGAACCTATTGATCTTTCTGATTTCTCAC

TGTGTAAAGCTTTTAACGGCAACCACTCAGGAACCGTACCAGAATGTAATGATTCTGACTCTGGTATTTC

ATTGAATGCAAGTTCTAGTGTAGCATCACCTGAACACTCTGTTGAATCATCTGCCTATGGAGATAAGACT

TTTGGTTGTAGTGATTCTGAAATGGAAGACATGGATAGCGCTCCTGGAAGCGTGCCGCAGGGCAATGCTA

GTGCATACTCTTTGCAGTTCCAGGATCAAGTGTTTTCTTCGGTGGGGCCAAGCACTCAAACACCTAGTCT

GCAGTGTACAAGCACGCCAAAGAAAGAACCCCCTGCTGGTCCAGGTCACCCCAAAGCTCCATTCACAAAA

GATAAACCTTCAAGCCGCCTTGAAGCTCATCTCACAAGAGATGAGCTAAGAGCAAAAGCTCTGCTGATCC

CTTTTCCTGTTGAAAAAATTGTCAATCTCCCTGTTGATGACTTCAATGAAATGATGTCTAAGGAGCAGTT

CAGTGAAGCCCAGCTTGCACTTATTCGAGATATACGCAGGAGAGGCAAGAACAAAGTGGCTGCTCAAAAT

TGCCGTAAAAGAAAACTGGAAAATATAGTAGAACTGGAGCAAGACTTGAGTAACCTAAAAGATGAGAAAG

AGAAATTGCTTAGGGAAAAAGGAGAGCATGACAAAAGCCTTCGCCAAATGAAAAAGCAGCTAACCACCTT

ATACCTTGAGGTCTTCAGCATGCTACGTGATGAAGACGGAAAGTCTTACTCTCCTAGTGAATATTCATTG

CAGCAAACTAGAGATGGCAATGTCTTTCTTGTTCCTAAAAGCAAGAAGTCAGAGACTAAACTTTGA

>av_p_crispus lcl|XM_009483699.1_cds_XP_009481974.1_1 [gene=NFE2L2] [protein=nuclear factor erythroid 2-related factor 2 isoform X2] [protein_id=XP_009481974.1] [location=1..1743]

ATGAACTTGATTGACATTCTTTGGAGGCAAGATATAGACCTTGGGGCAAGGCGTGAAGTTTTTGATTTTA

GTCAACGACGGAAGGAGTATGAACTCGAGAAACAGAAGAAACTTGAAAAGGAAAGACAAGAGCAGCTCCA

AAAAGAGCAGGAGAAAGCCTTGCTGGCTCAGCTGGAGTTAGACGAAGAGACGGGTGAATTTGTTCCTGTT

CAGCCAGCTCAGTGCATCCAGTCAGAAAATACTGAGCCACCAATCGGTTTCTCACAGACTTCAAAACCAG

AAGCAGAGGCCTTGTCCTTTGATGACTGCATGCAGCTCTTGGCAGAAGCATTCCCGTTTATAGACGACAA

TGAGGCTTCTTCAGCCGCATTTGAGTCACTGGTTGCTGCTGAGATCGATAGCAACGCAGTCTTCATTTCC

TCTGATCAAACTCAGCCACCTGATTCACCTGTTCTAGTTCCACTTACTGATGCTGAGAATATGCAGAACA

TAGAGCAAGTTTGGGAAGAATTATTGTCCCTTCCAGAGTTACAGTGTCTTAACATTGAAAATGATAACCT

GGCTGAGGTAACCACGATCACAAGCCCCGAAACCAAGCCAACAGAGATGCACAACAGCTACAACTACTAC

AGCTCATTACCCATCATGAGAAAAGATGTTAACTGCGGTCCAGATTTCCTGGATAGTATTGAGGGCCCCT

TTTCCAGCATTTTGCCACCAGAAGATACCAGCCAGCTGAGTGTGAACTCTTTAAATGACACGTCCCCTTC

AAACTCTGATTTCTGTGAGGATTTCTACACTGCCTTTATTGATACAAAGGCAAACGGTGACACAGCAACG

ACAAACACTATCAGTCAATCACTCGCGGAAATTCTAAGTGAACCTATTGATCTTTCCGATTTCTCACTGT

GTAAAGCTTTTAATGGCAACCACTCAGGAACCATACCAGAATGTAATGATTCTGACTCTGGTATTTCACT

GAATGCAAGTTCTAGTGTAGCATCGCCTGAACACTCTGCTGAATCATCTGCCTATGGAGATAAGACTTTT

GGTTGTAGCGATTCTGAAATGGAAGACATGGATAGTGCTCCTGGAAGTGTGCCGCAGAGCAATGCTAGTG

TGTACTCTTCACAGTTTCAGGATCAAGTGTTTTCTTCTGTGGGGCCAAGCACTCAAACACCTAGTTTGCA

GTGTACAAACACACCAAAGAAAGAACCCCCTGCTGGTCCAGGCCACCCCAAAGCTCTGTTCACAAAAGAT

AAGCCTTCAAGCTGCCTTGAAGCTCATCTCACAAGAGATGAGCAAAGAGCAAAAGCCCTGCAGATCCCTT

TTCCTGTTGAAAAAATCATCAATCTCCCTGTTGATGACTTCAATGAAATGATGTCTAAGGAGCAGTTCAG

CGAAGCCCAGCTTGCGCTTATTCGAGATATACGCAGGAGAGGCAAGAACAAAGTGGCTGCTCAAAATTGC

CGTAAAAGAAAACTGGAAAATATAGTGGAACTGGAGCAAGACTTGAGTAACCTAAAAGATGAGAAAGAGA

AATTGCTTAAGGAAAAAGGAGAGCATGACAAAAGCCTTCGGCAAATGAAAAAGCAACTAACGACCTTGTA

CCTTGAGGTCTTCAGCATGCTACGTGATGAAGATGGAAAGTCTTACTCTCCTAGTGAATATTCGCTGCAG

CAGACTAGAGATGGCAATGTCTTTCTTGTTCCTAAAAGCAAGAAGTCAGAGACTAAACTTTGA

>av_t_guttatus lcl|XM_010215694.1_cds_XP_010213996.1_1 [gene=NFE2L2] [protein=nuclear factor erythroid 2-related factor 2] [protein_id=XP_010213996.1] [location=73..1836]

ATGAACTTGATTGACATTCTTTGGAGGCAAGATATAGACCTTGGGGCAAGGCGTGAAGTTTTTGATTTTA

GTCAACGGCAGAAGGAGTATGAACTTGAGAAACAGAAGAAACTTGAAAAGGAAAGACAAGAGCAGCTCCA

AAAAGAGCAGGAGAAAGCCTTGCTTGCTCAGCTGCAGTTAGACGAAGAGACAGGTGAATTTGTTCCAATT

CAGCCAGATCAGCGTGTCGAGTCCGAAAATACTGAGCCACCAGACAGTTTTTCGCAGAGCACACATACTT

CGAAACCAGAAACAGAAGCCCTGTCCTTTGATGACTGCATGCAGCTCTTGGCAGAAGCCTTCCCATTCAT

AGATGACAATGAGGTTTCTTTTACTACATTTCAGTCACTGGTTCCTGCCCAGATGGATAGTAGTCCAGTC

TTCATGTCTTCTAATCAAACTCAGCCTGAAGTTGAATCACCTGAATCACCTGCCTTAGTTTCACTTACTG

ACGCAGAGAATATGCAGGACATAGAGCAAGTTTGGGAAGAATTATTGTCCCTTCCTGAGTTGCAGTGTCT

TAACATTGAGAACGATAACCTGGGTGAGGTAAGCACGATCACAAGCCCAGAACCAAAGTCAACAGAGATG

CACAACAGCTATAATTACTACAACTCATTATCTACGATGAAGAAAGATGTTACCTGTGGTCCAGATTTCC

TGCATAGTATTGAAGGTCCTTTTTCCAACATTTTACCACCGGAAGACACCAGCCAGTTGAGTGGAAACTC

TTTAAATCACACCTCTAATTCGAGCTCTAATTTCTGTGAGGATTTCTATGCCACCTTTATTCATACAAAG

GAGAACAGTAACACTGCCACAACAAACACTATCAGTCAGTCACTTGTGGATATTCTAAGTGAACCTATAG

ATCTTTCCAGCTTCTCTCTATGTAAAGCCTTTAATGGTGACCACTCAGGAACTGCACCAGAATGTAACGA

TTCTGACTCTGGTATCTCACTGAACGCCAATTCCAGTATCGCGTCGCCCGAGCCCTCTGTTGAATCCTCT

GTGCTCGGAGATAAGGCTCTGGGGTGCAGCGACTCGGAGCTGGAGGAGGCGGACGGCGCTGCGGGGAGCG

CGGCGCCGAGCAGCGCCCGCGCGTGCGCCCGGCCCTTCCCCGAGCGCGCGCTCTGCGCTCTGGGGCCCGG

CCCGCAGCGCCCCGCGCTGCCCTACGCCAACGCGCCCAAGAAGGAGCCGCCCGCCAGCCCGGGCCACCCC

AAAGCTCCCTTTGCAAAGGACAAACCCGCCAGCCGCCCTGGAGCTCCTCTCACCAGAGATGAGCAACGAG

CAAAAGCTCTGCAGATCCCCTTTCCTGTAGAAAAAATCATCAATCTCCCTGTTGACGACTTCAATGAAAT

GATGTCTAAGGAGCAGTTCAGCGAGGCCCAGCTGGCGCTTATCCGAGATATACGCAGGAGGGGCAAGAAT

AAAGTGGCTGCTCAGAACTGCCGTAAAAGAAAACTGGAAAATAGAGTGGAACTGGAGCAAGATTTGAGTA

ACCTAAAAGACGAGAAAGAGAAATTGCTTAAAGAAAAAGGAGAGAATGACAAAAGTCTTCGTCAAATGAA

AAAGCAACTTACCACCTTATACCTTGAGGTCTTCAGCATGCTGCGTGATGAGGATGGAAAGTCTTATTCT

CCTAGTGAATATTCACTGCAGCAAACTAGGGATGGTAACGTCTTTCTTGTTCCTAAAAGCAAGAAGTCGG

AGACTAAATTCTGA

>re_a_carolinensis lcl|XM_003226765.2_cds_XP_003226813.1_1 [gene=nfe2l2] [protein=nuclear factor erythroid 2-related factor 2] [protein_id=XP_003226813.1] [location=524..2317]

ATGGAGGTCGAGATGCCGCAGGATATGAATCTGATTGACATTCTCTGGAGGCAAGATATTGATCTTGGAG

CACGGCGTGAAGTTTTTGATTTTAGCCAAAGAAAGAAAGCTTCTGAGCTTGAGAAACAGAAGAAGCTGGA

AAGAGAGAGACAAGAACAGCTTCAGAAAGAGCAAGAGAAAGCCTTCCTTGCCCAATTGCAGCTGGATGAG

GAAACAGGTGAATTTGTTCCTATCCAGCCTACTCAAGCCATTGAATCAGGAAACACTGCCATATCCAATA

ATTACTCTCAGAACGTACATATTTCCAAGCAAGATGCGGACAATCTGTTTAGTGACGTGTTTGATGACTG

CATGCAAGTATTGGCAGAAACGTTCTTGTTTGTAGAGGACCCAAAGGTTTCTCCAGTTGAATTTCAGCAA

GTGGCTCCGTCTGACATTGAGAGCAACCAAGTATTTGTTGACCCTAATCATATGCAGCCGCTTGATTCAT

CTGTCCTTCAGCCTGCCATTTCAGAGTTTGCAATGACTCCTGGTGAGAGCACACAGGACATGGAACAAGT

GTGGGAAGAATTGTTGTCTATTCCAGAGCTCCAGTGCCTTAATATTCAAAATGACAACCTGGCAGAAGTA

ACCCCAAACACCTGCACAGCAAACACAATGTCTGAGGCTGCAATCGACTTTACTTTCTACAACCCATTAC

CCCCCATGGAGAATGAAGTTTCTACCTGCAGTCCAGAGTTTCTGAAGCCTTTGGAGGCCTCTTATTCTGG

CATTTTACTGCCAGATCTAAGCCAAAATAACACATCATCCACAAGCAGTGACTTTTGTGAAGATTTCTAT

CCTGATTTTATTGATGTCAAAGCGAACAACAGCATAACCAGCCCACCACCCAATTTTGTGGACCAGGCTC

TTACTGGCTTTTTAAATGAACCTATTGATCTGTCTGATTTTGCTCAGTGCAAAGCTTTTAACTGTGATCT

TGCAGGAAACCCACAAGAATGTACTGATTCTGACTCTGGCATTTCACTGAACAGAAGTCCCAGTACAACT

TCTCCAGCTCATTCTATTGATTCGTCTTCTATCTGTAGAGATACAGGCTTTGGATGCAGTGATACTGAAA

TAGAAGAGATGGATAGCGCCCCTGGGAGTGTGCAACAGAGCAATACCCAAATGCCTGTGTTTCAGTTTCT

GCTACCACTCTCTCCACCTGTAGAGCAAAGGAGCCCCACAGCTGCTTCACCAGTTAAAGGTGAGGTCAAA

AGAGAATTGCCTGCCAACCCTGGTCATTCTGAGGCTCCGTTCATGAAGGACAAATCCTATAGCCAAGATG

AAGCACATCTCACAAGAGATGAGCTTAGAGCAAAAGCTCTGCAGATCCCTTTTCCTGTTGAAAAAATCAT

CAACCTCCCTGTAGATGACTTCAATGAAATGATGTCTAAGGAGCAATTCACTGAGGCCCAGGTTACGCTT

ATTCGTGATATACGAAGGCGAGGTAAAAACAAAGTGGCTGCTCAAAACTGCCGTAAAAGGAAACTAGAAA

ACATTACGGAGCTGGAGTATGATTTAGGTTACCTTAAGGATGAGAGGGAGAAGCTTCTGAAGGAGAAAGC

AGAGAATGATAAAAGCCTACACCTGTTGAAAAAGCAGCTAAGCACACTATACCTTGAGGTCTTCGGCATG

CTCCGAGATGAAGATGGAAAGCCTTACTCAGTTAACGAATACTCATTACAGCAAACAAGAGATGGTGGTA

TCTTCCTTGTTCCAAAGACCAAGAAACCAGGGACTAAAATGTGA

>re_c_mydas lcl|XM_007060512.1_cds_XP_007060574.1_1 [gene=NFE2L2] [protein=LOW QUALITY PROTEIN: nuclear factor erythroid 2-related factor 2] [protein_id=XP_007060574.1] [location=1..1899]

ATGAGCAGTTTTTACAATTACTGTTTTAACCACAAAAACCTGCTAGTCCATGAAAATAACAAATACAACA

TGTTAGTTTTTGACCAAACAATTATTTCCTCTGATATGATGCAAGAGCTTACACAACTGAGGAGAGAACA

AACGGCCACTTGTGAAGACATGAACTTGATTGACATTCTTTGGAGGCAAGATATAGACCTTGGGGCAAGA

CGTGAAGTTTTTGATTTTAGTCAAAGACAGAAGGAATATGAACTCGAAAAGCAGAAGAAACTTGAAAAGG

AGAGACAAGAACAGCTCCAAAAAGAGCAAGAAAAAGCTTTACTGGCTCAGCTGCAGCTGGATGAAGAAAC

AGGTGAATTTATTCCCATTCAGCCTGCCCAGCACATTGAGTCAGACAACACAGGCATGCCAACCAATTTT

TCAAAGACTACGCATATTTCAAAACCAGAAACAGATGCCTTGTCCTTTGATGACTGCATGCAGCTTTTGG

CAGAAACATTCTCGTATGTAGATGATGATGAGGTTTCTTCAGCTGCATTTCAAGTGTTGGTACCTGCTCA

TATAGATAGCGACGCAATCTTCATTACTTCTAATCAGACTCAGCCACCTGAATCATCAGTCCTTCAATCT

TCTGTTGCTGAAGTAGATAATATGCAAAACATAGAGCAAGTTTGGGAAGAATTACTGTCCATTCCAGAAC

TACAGTGTCTTAACATTGAAAATGATAACCTGGCTGAAGTAACCACTATTGCAAACCCAGAAACCAAGCC

ATCAGAGATTCACAGTAACTTCTACAACTCATCATCCATTACTGTTAATTGCAATTCAGATTTCCTCAAC

ACTTTTGAGGATTCCTTTTCTAGCATCCTACCACCAGAAGACCTCAGTCAGCTGGGACTGGACTCTTTAG

ATACCACTTCATGTTTAAGCTCCAACTTTTCTGAGGATTTCTACTCCACCTTCGTTGATCCAAAGGTGAA

TGGTGACACAGCAGCACCACATGTTGTCAGTGAGTCATTTGCTGAAATTCCATATGAACCTATTGATATT

TCTGATTTCTCACTGTGTAAAGCTTTTAATGGTGACCATCAAGGAAATGCACCAGAATGTAATGATTCTG

ACTCTGGTATTTCATTGAACGCACATTCCAGTACTGCATCACCTGAACATTCTGTTGAATCATCTGTCTT

TGGAGATACAGCTTTTGGATACAGTGATTCTGAAATGGAAGAAATGGAGAGTGCTCCTGGAAGTCTGCAA

CAGAGCAATGCTCAAATGTATTCATTGCAGTTCCATGATCCAGTCTCTCCTTCTCTGGGGCCAAGCACTA

AAAAGTCTGATCTGCAATGTGTAAATACACCAAAGAGAGAACTACCTGCCAGTCCAGGCCACCCCAAAGC

TCCATTTACAAGAGATAAACCTTCACGCTACCCTGAAGCTCATTTCACAAGAGATGAGCAAAGAGCAAAA

GCTCTGCATATCCCTTTCTCTGTAGAAAAAATCATCAACCTTCCTGTGGATGACTTCAATGAAATGATGT

CTAAGGAGCAGTTCAATGAGGCCCAGCTTGCACTTATTCGAGATATACGCAGGAGAGGCAAGAATAAAGT

GGCTGCTCAAAACTGCCGTAAAAGGAAACTGGAAAACATAGTGGAACTGGAGCAAGACTTGGGTCATTTA

AAGGATGAAAAAGAGAAATTACTTAAAGAAAAAGGAGAGAATGACAAAAGCATCCGTCTAATGAAAAAGC

AGCTTACCAACTTGTATCTTGAGGTCTTCAGCATGCTACATGATGAAAATGGAAAGCCTTACTCTCCCAG

TGAATACTCACTGCAGCAGACAAAAGATGGCAGCATCTTCCTTGTTCCTAAAAGCAAGAAGCCAGAGACT

AAATTTTGA

>re_p_bivattatus lcl|XM_007424606.1_cds_XP_007424668.1_1 [gene=NFE2L2] [protein=nuclear factor erythroid 2-related factor 2 isoform X2] [protein_id=XP_007424668.1] [location=12..1763]

ATGCCGCAGGATGTGACTCTAATTGACATTCTCTGGAGACAAGACATAGATCTTGGAGCAGGGCGTGAAG

TTTTTGATTTTAGCCAAAGAAAGAAGGAATATGAACTTGAGAGGCAGAAGAAGCTTGAAAGTGAGAGACA

AGAACAGCTTCAGAAAGAGCAAGAAAAGGCCTTTCTTGCCCAGTTGCAGCTGGATGAGGAAACAGGGGAA

TTTGTTCCTATCCAGCCAGCTCAAGCCATTGAATCTGGAAATTCCGCCATATCCAACAGTTATTCACAGA

GTGTACACATTTCCAAACAAGATGCAGATGATCTGTTTGATGACTGCATGCAGATTTTAGCAGAAACGTT

TCCATTTGTGGACGAGAGTGAGATTTCCCCAGCTGAATTTCAACAAGTGGCACCTTTGGAAATGGCTACC

AACCAGGTCTTTGTTGATTCTAACCATATGCAGCCACTTGACTCATCTGTACTTCAGTCTACGATTCCAG

AGTTGTTAGATACAAAAGTTGAGAACACACAAGATATAGAACAAGTGTGGGAAGAACTCCTGTCTATTCC

AGAACTGCAGTGTCTTAATATACAAAATGACAGCCTGGCTGATGTAACACCAAACTCATTTGCAGTAAAT

GCCACTTCAGAGGCTGCTGATAACTTTACCCTCTACAATTCATTATCTGCCATGGAGAAAGAAGTTAACT

GCAGCCAAGAATTCCTGAAGCCACTGGAGGATCCCTATTCTAGCATGGTGCTGCCAGAAGATCCTAGCCA

ACATAGCACTGATTTCTGTGAGGATTTCTATTCCCTTATTGACGGAAAGATGAACAGCAGAGTGGCTACC

CCACCATCTCATTTTGTGGATCAGGCACTTGCTGGCTTTTTAAGTGAACCTATTGATCTTTCAGATTTTG

CTCAGTGCAAAGCTTTTAACCAGGACCTTGCAGGAAATGCCCCGGAGTGTAATGATTCCGACTCTGGTAT

TTCACTGAATGCAAGTCCCAGTACAACATCTCCAGCTCATTCTGTTGAGTCGTCTTCGGTCTATAGGGAT

ACAAGCTTTGGATGCAGTGATTCAGAAATGGAGGAGATTGATAGTGCCCCTGGAAGCGTGTCACAGAGCA

ACACAAAGATGCATTCATTTCAGCTGCAGGCTCCTGTCTCTCCATCTCTAGAGCAAGGCACCCCAAAGCC

TGACATACCACTTACTAGTACACCCCAAAGAGAATTGCCTGCCAATCCTGGCCATGTCGAAGCTCCATTT

ATGAAGGACAAGTCCTTTAGCCAACCAGAAGCTCATCTCACTAGAGATGAGCTGAGAGCAAAAGCTCTGC

AGATCCCTTTTCCTGTTGAAAACATCATCAACCTGCCTGTGGATGACTTCAATGAAATGATGTCCAAGCA

GCAGTTCAGCGAGGCCCAGCTCACTCTGATTCGTGACATTCGAAGGCGAGGCAAGAACAAAGTAGCTGCT

CAAAACTGCCGTAAAAGGAAACTGGAAAATATTACTGAGTTGGAGCACGACTTGGATTACCTGAAGGATG

AGAAAGAGAAGCTCCTGAAAGAGAAGGCGGAGAATGACAAAAGCTTGCAGCTGCTGAAAAAGCAGCTGAG

CACCTTGTACCTTGAAGTCTTCAGCATGCTCCGGGATGAAGATGGCAAGCCATATTCCCCTAATGATTAC

TCACTGCAGCAAACCAGAGATGGGAGCATTTTTCTTGTTCCTAAGAGCAAGAAGCTAGAGACTAAATTCT

GA

>re_a_mississippiensis lcl|XM_006271088.1_cds_XP_006271150.1_1 [gene=NFE2L2] [protein=nuclear factor erythroid 2-related factor 2 isoform X2] [protein_id=XP_006271150.1] [location=61..1845]

ATGGAGGTCGAGGTGCCGCAGGACATGAACTTGATTGACATCCTTTGGAGGCAAGATATAGATCTTGGGG

CAAGACGTGAAGTCTTCGATTTAAGTCAACGAAAGAAAGAATATGAACTTGAGAAGCAAAAGAAACTAGA

AAAGGAGAGACAAGAACAGCTTCAAAAAGAGCAAGAAAAAGCACTATTGGCTCAGTTGCAGTTAGATGAA

GAAACAGGTGAATTTGTTCCCATTCAGCCAACGCAGCACATTGAGTCAGAAAATACTAGAGCGCCAATCA

GTTTTTCACAGAATACACATACTTCAAAATCAGAAGCAGATGCCTTGTCTTTTGATGACTGCATGCAACT

CCTGGCAGAAACTTTTTCATTTGTAGATGATAATGAGGTTTCTTCAGCTGCGTTTCAATCACTAGTTCCT

GCTCCGGTTGATAGCAACACAATCTTTATTAGTCCTAATCAGACTCAGCCACCCGAGGCATCTGTCCTTC

AATCCCCGGTCACTAATGGAGACAGCACACAAAACATAAATCAAGTTTGGGAAGAATTATTGTCCATTGC

AGAACTACAGTGCCTTAACATTGAAAATGATAACCTGGTTCAAATAATGAACGCTTTCACAAGCCCGGAA

GCCAAGCCAGCAGAGATGCACAATAACTGTAACTTCTACAACTCGTTATCTATAACGGATAAAGATGTTA

CCTGCAATCCTGATTTCCTTGATGCTTTTGAGGAGGGTCCCTTTTCTAGCATCTTACCACCAGAAGACCT

CAGCCAATTGAGAGTGAACCCTTCAAATACTACTCCCTCTTCAAGGTCTGACTTATGTGAAGATTTCTAT

TCCACCTTTATTGATACAAAGGTGAGCAATGACACAGCAGCCCCAAATGTTATCAGCCAATCTTTAGATG

ATATTCTAAGTGAACCCATTGATCTTGCAGACTTCTCCCTTTGTAAAGCTTTCAACAGTGACCTTTCTGG

AAGTGCAGCAGAAGGTAATGATTCCGACTCTGGTATTTCCCTGAACACAAGTCCTAGCACAGCATCACCT

GAATACTCTGGTGAATCATCTGTCTGTGAAGATAAAACTTTTGGGTATAGTGATTCTGAAATGGAAGAAA

CGGACAGTGCACCTGGAAGTATGCAACAGAGCAATGTTAATGTGTATTCATTGCAATGCCATGATCAAAT

GTCTCCTGCCTTGGGGCCAAGCACTCGAAAGTCTGATTTGCAATGTGCAAACACACCAAAGGGAGAGCTA

CCTGCCAGCCCAGGCCACCCTAAAGCTCCATTTACAAAAGACAAGCCATCTGGCCACCTTGAAGCTCATC

TCACAAGAGATGAGCAAAGAGCAAAAGCTCTGCATATCCCTTTCCCTGTAGAGAAAATCATCAACCTCCC

TGTGGATGACTTCAATGAAATGATGTCTAAGGAGCAGTTCAATGAAGCCCAACTTGCGCTTATTAGAGAT

ATACGCAGGAGAGGCAAGAATAAAGTGGCTGCTCAAAATTGCCGTAAAAGGAAACTGGAAAACATAGTGG

AACTGGAGCAAGACTTGGGCCATCTCAAGGATGAAAAAGAGAAATTACTTAAAGAAAAGGGAGAGAATGA

CAAAAGTCTCCGCTTAATGAAGAAGCAGCTTAGCACCTTGTATCTTGAGGTCTTCAGCATGTTACGTGAT

GAAAATGGAAAGCCTTACTCTCCTAATGAGTACTCACTGCAGCAAACAAGAGATGGCAATGTCTTCCTTG

TTCCTAGAAGCAAGAAGCCAGACACAAAATTTTGA

>am_x_tropicalis lcl|NM_001007489.2_cds_NP_001007490.1_1 [gene=nfe2l2] [protein=nuclear factor erythroid 2-related factor 2] [protein_id=NP_001007490.1] [location=88..1857]

ATGATGGAGATCGAGATGCCCCTGCCACTGCAGTCACAACAGGATATGGATCTGATTGATATTCTTTGGA

AGCAAGACATTGACCTCGGTGTTAGCCGTGAGGTTTTCGACTATAACCAAAGACAGAAGGAAAATGAATT

GGAGAAACAGAAGAAGCTTGAAAAGGAGCGGCAAGAGCAGCTGCAGAAGGAACGGGAAAAAGCACTCTAC

GCCCAACTGCAATTAGATGAGGAGACAGGGGAATTCATCCCAATCCAACAGGCTGCACCTATTGAGACTG

CAGCAGTCACGCAGGAGCTTGCCAGTTCCATTGAGGTCAAACCCAGTTTGGTACATGATTTATCCTTTGA

TGAATGCTTGAAGATTTTGGGTGAAACATTCCAGTTAGGACCAGCTAATGAGGAATCCTCTTTGGCATAC

CAGACACTAGAACCCAGTGATCCTATTGAAACAAACCAGACCTTCCTCCAGTCTGAACCAAATCCGGTAC

CAGCTGGCACGCTGAGCAGCATACCTGCGGAAGGAGAGATTATGCATGAAATGAATCAGGCTTGGGAGGA

GTTATTGTCTATTCCTGAGTTACAGTGCCTTAACAATGAGATTGAAAACATGGTGGACCTAAGCATGTAT

ACAAACCAAGAATCCATCACAATGACAGAGACTCCAGACACTTACAGCTTCCTTAGTCCCCTGTCCACTA

TTGAAAAACCACATGAAAGCAGCACTGTTTTCTCCAGTGATCTTGTGGATACGTTCACTAGTAGTCTACC

ATCAGTAAACACAAATACAGCCTTTAATGTTGAGTCATTCTGCGATGATATATTTACACTTGACCCAAAA

GTGACCAATGTTGTGCCTTTAACAGACAATTCAGGCCAGTTGCTGAATGAGCTTTTGAATGATAACGTTG

ATATTACAGACTTGTCATTATGTAAAGCTTTTAATGGGAACAACCAACCAGAATTCAATGATTCTGATTC

TGGTGTTTCTGTCAATGCCAGTCCATGTGCAACATCACCTTCCCAGTCCATGAGTGGTTCCGTCTATGGT

GAGCCTCATCATAGCTACAGTGATTCAGACATGGAAGATATGGACAGCACTCCAGAGACTGCACAGCAAA

AACCTCCAGACAATTTTACTGCAGCATTTACTGAGGACACATACTTCACTCTTTCGCCTTTTGTCTCGCA

TGACACAGATCCCTTTGATATAGAAGCTCACACCCCTTCGGCAAAAGAGATACCTGCTAGCCCAGGCTAT

AGCAAGGCTCCGTTTGCCAAGGACAAGTATTTAAGCCGCCAAGAAGCTCGTTTCACCAGAGACGAGCAAA

GAGCAAAGGTTCTCAACATACCATTCTCTGTCGATAAAATAGTTAACCTTCCAGTGGACAACTTTAATGA

GTTGATGTCCAAGTATCAGTTTAACGAGGCCCAGCTTGCCCTCATAAGAGATATAAGGAGGCGGGGCAAA

AATAAAGTAGCTGCTCAGAATTGTCGGAAGAGGAAGATGGACAACATAGTGGAATTGGAGACTGATCTGG

ACAAGCTTAAGTATGAAAAAGAGAAATTACTTGCTGAACGAGGAGAGTACAACAACAGCCTTAGTCAACT

GAAGAAGAAGCTTGGCGCCCTGTACATGGAGGTCTTTAATAAGCTGCAAGACGAGAATGGGCAACCATAC

TCCCCTCATGAATACTCCCTCCAGCAGACAAAGGAGGGAAATATTTTCCTTGTTCCAAAAACCAAGAAAG

TTAGCATAAAGAAGGAATAG

>am_x_laevis lcl|BC043997.1_cds_AAH43997.1_1 [gene=MGC53355] [protein=MGC53355 protein] [protein_id=AAH43997.1] [location=115..1890]

ATGATGGAGATCGAGATACCCCTGCCACTGCAGTCACAACAGGATATGGATCTGATTGATATTCTTTGGA

AGCAAGACATTGATCTCGGTGTTAGCCGTGAGGTTTTTGACTATAATCAAAGGCAGAAGGAAAATGATTT

GGAGAAACAGAAGAAGCTTGAAAAGGAAAAGCAAGAGCAGCTGCAGAAGGAACAGGAAAAAGCACTCTAC

GCCCAACTGCAATTAGATGAAGAGACAGGGGAATTCATCCCAATGCAACAGGCCACACCTACTGAGACTG

CAGCAGACACGCAGGCCCTTGCTGGTTCCATCCAGGACAAACCCAGTCCAGTACATGAATTGTCCTTTGA

TGAATGCTTGAAGATTTTGGCTGAAACATTCCAGATAGGGCCATCTAATGAGGATTCCCCTGTGGCATAC

CAGACACTGGAACCCAGCGCTCCTATAGAAACAAACCAGATCTTCTTCCAGTCTGAACCAAATTCAGTAA

CAGCTGGCACTCTGAATAGCATACCTGCACAAGGAGAGACCGTGCATGAAATAAATCAGGCTTGGGAGGA

GTTATTGTCTATTCCTGAGTTGCAGTGCCTTAGCAATGAGATTGAAAACATGGTGGAGCAGAGCATGTAT

CCAAACCCAGAATCCACTACAATGACAGAGACCCAAAACACTTTCAGCTTCTTCACTCCACTGTCGGCCA

TGGAAAAACCACAAGAAAATAACACTTCAGTTTTCCCCACTGATTATGTGGATACGATTGCTAGTAGTGT

ACCGTCAGTAAATACAAATGCCACCTTTAACGTTGAGTCATTCTGTGATGATATATTTACCCTTGTTGAC

CCAAAAGTGACCAATATTGTGCCTTTAACGAACAATTCAGGCCAGTTGTTAAAAGAGCTTTTGCATGATA

ATGTTGATATTACAGACTTGTCATTATGTAAAGCTTTTAATGGGAGCAACCAACCAGAGTTCAATGATTC

CGATTCTGGTGTTTCTGTTAATACCAGTCCATGTGCAACATCACCTTCCCAGTCCTTGGGTGGTTCTGCC

TATGGTGAACCTCATTATGGCTACAGTGATTCAGACATGGAAGATATGGACAGCACTCCAGAGATTGCAC

AGGAAAATCCTCCAGACAATTTTACTGCACCATTTACAGAGGACACATACTTTAGTCTTTCACCTTTTAT

TTCACATGACACAGATCCCTTTGAAGTTAAAGCCCACGACTCTCCAGCAAAAGAGATACCTGCTAGTCCT

GGCTATAGCAAAGCTCCATTTGCCAAGGACAAGTCTTTAAACCTCCAGGAAGCCCGTTTCACCAGAGACG

AGCAAAGAGCAAAGGTTCTCAACCTACCATTCACTGTCGAAAAAATAGTTAACCTGCCCGTGGACAGCTT

CAATGAGATTATGTCCAAGTATCAGTTTAACGAGGCCCAGCTTGCCCTCATAAGGGATATAAGGAGGCGT

GGCAAAAATAAAGTAGCAGCTCAGAATTGCCGGAAGAGGAAGATGGAGAACATTGTGGAGTTGGAGACTG

ATCTGGACACGCTGAAGTATGAAAAAGAGAAGTTACTTGCCGAACGAGGAGAGTACAACAACAGCCTTAG

TCAACTGAAGAAGAATCTTGGCAACCTATACATGGAGGTCTTTAATAAGCTGCAAGACGAGAATGGAAAG

CCATACTCTCCTCAAGAATACTCCCTTCAGCAGACAAAAGAGGGAAATATTTTCCTTGTTCCGAAAACCA

AGAAAGTTAGCATTAAGAAGGAATAG

>fi_d_rerio lcl|NM_182889.1_cds_NP_878309.1_1 [gene=nfe2l2a] [protein=nuclear factor erythroid 2-related factor 2] [protein_id=NP_878309.1] [location=42..1802]

ATGATGGAGATTGAAATGTCTAAAATGCAGCCAAGCCAACAGGACATGGATCTGATCGATATCCTGTGGC

GGCAGGATGTGGATCTGGGCGCGGGCCGTGAGGTGTTCGACTTCAGCTACCGGCAGAAGGAGGTGGAGCT

GCGCAGGCGGAGGGAGCAGGAGGAGCAGGAGCTGCAGGAGCGTCTGCAGGAGCAGGAGAAGACACTGCTG

GCTCAGCTGCAGCTCGACGAGGAGACCGGAGAGTTCCTGCCGCGCAGCACACCGCTCACACACACACCTG

AAGCAGACGGAGGAGGAGCGGGAGAAATCACACAGAATGGGGCTTTTGCAGAACAGGAGGCCGATCCCAT

GTCATTCGATGAGTGCATGCAGCTCCTGGCTGAAACCTTTCCACTAACAGAGCCGGCTGAGTCGGCTCCG

CCTTGCCTGAACACCTCCGCTCCACCTTCCACTGATCTCATGATGCCCGCAGACGTCCCGGCGTTTACCC

AGAATCCTTTGCTGCCAGGATCTCTGGATCAGGCCTGGATGGAGCTGCTGTCACTCCCAGAGTTGCAGCA

GTGCCTCAACATGCCAATGCAGGAGACGTTGGATATGAATGCATTCATGAAACCTTCCACAGAAGCACCA

ACCCAAAACTACAGCCAATATCTACCCGGGATGGACCATCTCGGCTCGGCTCAAACAGAAGTGTGTCCTC

CTGAATTCACCAACACCTATAATAGATCCTTCAACACTATGGTGTCACCCAACATGAATCAACTGAGTCT

GAACGTCCCGGATGTGGGAGCCGAGTTTGGCCCTGAAGAATTTAACGAGCTGTTTTATCCAGAGATGGAG

GTAAAAGTGAACAACCCTCCGATTACATCAGATGGCGGAAATATGGTCGGCGATCCTCCTGTAAACCCAA

TAGATCTACAGAGCTTCTCACCGGGAGATTTCAGCTCAGGGAAACCAGATCCAATCGTGGAGTTTCAAGA

TTCTGACTCGGGTTTGTCCCTAGATGCAAGTCCTCACATGAGCTCTCCGGGGAAGTCCATAACCGAAGAC

GGATCCTTCGGATTTAGTGATTCTGACTCGGAGGAGATGGAAGGAAGTCCGGGAAGCATGGAGTCAGATT

ACAATGAGATATTCCCATTGGTGTACCTCAACGATGGCTCCCAGACTCCACTCTCTGAGAAATCCTCGAC

GGAGAAACAAGAGATGAAACTGAAAAACCCAAAGATGGAGCCGGCGGAGGCTAGCGGACACTCCAAACCT

CCGTTCACCAAAGACAAGCTGAAGAAGCGCTCCGAGGCGCGGCTCTCCCGTGACGAACAGAGAGCGAAAG

CCTTGCAGATCCCGTTTACCGTGGATATGATCATCAATCTGCCCGTAGACGACTTTAACGAGATGATGTC

CAAACACCAGCTCAACGAAGCCCAGCTAGCGCTCGTTCGGGATATCCGCCGACGAGGCAAGAACAAAGTG

GCGGCGCAGAACTGCCGCAAGCGCAAGCTGGAGAACATCGTGGGATTGGAGTACGAGCTGGACTCGCTGA

AGGAGGAAAAAGAGCGTCTGATGAAGGAAAAGAGCGAACGCAGCAGCAACCTGAAAGAGATGAAGCAGCA

GCTGAGCACGCTCTATCAGGAGGTTTTCGGGATGCTTCGAGATGAGAACGGAAAGGCTTTCTCGCCTAAC

GAATTCTCCCTTCAGCACACCGCGGACGGCACTGTTTTCCTAGTTCCTCGCCTTAAAAAGACTCTTGTGA

AGAATATCTAG

>fi_c_milii lcl|XM_007890062.1_cds_XP_007888253.1_1 [gene=nfe2l2] [protein=nuclear factor erythroid 2-related factor 2 isoform X1] [protein_id=XP_007888253.1] [location=46..2028]

ATGCGCGCAGGGGGCGTCCGGCACAGACCGCGATCCGAGCCGCTGAGAGGAACGGGGAGGGCGGCGCCGG

GTGTGTTTTACGCTGAAAGTCAGCAGTCGGTCGTCAGTCGTGTCAGTCAGACAGCAACCGGGAACAACAT

GACTGACATCCAGCGGCTCCCGATACAGCAGCAGAGTCAGCAGGACAGAGATTTAATTGACATCTTGTGG

AGGCAAGACATAGACCTTGGTGTTGTTCGTGAGGATTTCGATTACAACTACAGACAAAAAGAATATGCAC

TAGAGAAACAGAAGAAACTTGAGAAGGAAAAGCAGGAACAGCTCCAGAAAGAGCAGGAGAAAGCTCTACT

GGCACAACTGCAGTTAGATGAAGAGACGGGTGAATTTGTTGCCATTCGGCCAGCAAAGAACAGTGAGCCT

GCAAACACTGAAGGATTGACGGAATCTATACAGATACCAAGGACAGCAGAACAAGATAATGAGGCTCTGT

CATTTGATGAATGCATGCAGCTTCTTGCAGAGGCATTCCCATTTGTAGAGGATATAGAGACTGTTCCACT

TGAAGCTACAGTTCCTCTAGAACCTTCAGTTCCAATTCCAGCTCAGACTAGCTCTCAGCAAATGGCTCCT

CATGAGACCCAGCAAGCAGAAACTTCAGTCCTGCCATCTGCAGCACCCGAGTCAAACTCTCTGGAGGACT

TAGAGCAAACCTGGCAGGAGCTACTTTCAATTCCAGAGCTTCAGTGGCTGCACATGCAGAATGAGCACTT

CGGTGGTACTGCAGGTTTTTCTTCAAGGAGCAAAGCATCCGAGATACAAAGTGACGGTTACATTGCCACA

TTGCCAAGCGATCAAATGGTGACGGAAAGCAATCACAGTTTCCTCTCTCTTTTTGACCGTCCCTATCAAG

AAATAATGCCACCCGAGAGTCAAGACATGATCCAGCTCAAAACAAATGCCTCAGACAATGCAAACTCTTC

ATTCAGTAACAATTTCAATGGTCTGTTTTGCTCAACCTTTGTGAATACCCAGAGAAACAGCAATCTTTCC

CCACTTACCACCATGACCGATTCCCTTACTGGGATACTGGATGATCCTCTTCTTGAACAAATTACCATTT

CTGACTTGGCAATGAATGAAAATTTTGACTGTAAGCAACCACCAAATTTTCCTGAGGTTCCGGATTCAGA

CTCTGGCCTGGGTTCAAGCCCCAATACAGCTTCTCCACACAATTCAATGGGATCATCTATCTGTGGAGAT

GCTCCTTATAGTCATGGTGATTCTGACATGGATGACCTTGAAAGCAGTCCTGACAGTGTTAAACCAGAGT

TTCCTGAGATGTACCCAATGCAGTATCAGAATGAAGACCAATATCAGACTCCCTCTCTTCAAGATCTTAC

AAAACCCAGTCCTTGCCTGAATCTTGACACCAGACAAACACCAAAGGATGAGCTACCAGTAAGCCCCGGC

CACAGGAAAGCCCCGTTCACCAAAGACAAGCATTCAAAACGGGTGGAAGCCCGGCTCACCAGAGATGAGC

AGCGAGCTAAAGCACTGAAAGTTCCTTTCTCTGTCCGCAAGATTATCAACCTCCCTGTGGATGATTTCAA

TGAGATGGTATCCAAGTACCAACTTAATGAGCCTCAGCTTGCCCTAATTCGTGACATTCGCCGTCGTGGC

AAGAATAAGGTGGCAGCTCAGAATTGTCGGAAGCGGAAACTGGAAAACCTTGTTGGTCTGGAACAAGATC

TGGATAGTCTTGAAGATGAAAAGGAAAAACTCCTTGAAGAAAAGGGAGAGCATAACAAAAGCCTGCACAT

AATCAAGCAGCAGCTGAACAGTCTATATCGTGAAGTGTTTAGTATGTTACGAGATGGAGACGGCCACCCA

TATTCCCCCAGTGAATACTCATTGCAGCATACAAGTGATGGCAGTGTCTTTCTTGTCCCAAGGAGCAAGA

AACTAGAGATAAAGAGAGAATAA

>fi_l_chalumnae lcl|XM_006004969.1_cds_XP_006005031.1_1 [gene=NFE2L2] [protein=nuclear factor erythroid 2-related factor 2 isoform X1] [protein_id=XP_006005031.1] [location=162..1934]

ATGATGGAGATTCAGTTGCCACCAACACAACAAAATCAACAGGACATCGATTTGATTGATATTTTGTGGA

GACAAGATGTAGACCTTGGTGCACGACGGGAAGTTTTTGATTACAGTCATAGACAGAAGGAGTATGAGCT

TGAGAAAAAGAAAAAACTTGAAAAGGAGAGACAAGAACAGCTTCAAAAAGAAAAAGAAAAATCCCTGCTT

GCTCGGCTGCAATTGGATGAAGAGACGGGTGAATTTGTTCCTATTCAGCCAGCACAGCAATTCGAACCTG

AACCTTCTCCTGTACCTACTGAGCCTGCACAGAATACCAGCATTTCAGAGCAAGAGAGTGAAGCCCTATC

ATTTGATGAATGTATGCAGCTTCTCGCAGACACATTCCCATTTGTAGATGATATTAAGGTTGAAAATAGC

CCCGTCTTTGTGGCACCTCGGCAAACAGACTCTTCTATTCTTCATCCCACAATGCCTGATCCAAACTCAA

TGCAGGATGTGGAGCAAGTCTGGCAAGAGCTGTTATCTATTCCAGAGCTGCAGTGTCTGAATATACAGAG

TGAGAATATGGCTGACCAGACCACTACAACCAGCACAGCAGGGACTGACAATGACAACTGCAGTTTCTTT

ACTTCTCTTGTGCTGATGGATAAACCAGTATTGGGCTGCAGCCCACAGGTTCCCAACACATTTGAGAATT

CATATCATACTCTAGTGCAGCCTGAAAACCTTAATCAGATTGGAGTAAGTCTTTTAAACACAAATGTATC

TCAGAGTTGTGACAGTTTCTGCAACATATTTTATTCCAGTCTTCTAAGTCCAGAAAACGAGAACAATATT

CCTCCAGTGAATAATGCAAACCATTCACTTACTGGTCCTTTAGATGAATCTCCTCTTAAATCTATTGACA

TCGTTGATCTATCAGTATGTAAAGGTTTTGAAGCTGATTGTTCTACAGATATGTCAGAATTTCCTGATTC

AGACTCTGCAATGTCTATAGATGCAAGTCCAAATGCAACTTCACCAGTAAACTCAGCAAAATCTTCTATG

TATGGAGATGCTCCCTTTGGATACAGCGATTCTGAAATGGAGGACATGGATAGCAATCCAGGAAGTGTTC

AAAAAAACTATTCTATTTGCCCAACAGAATTCCAGGGAGATGCTCAATATCAAACTGCACCTTCTCTGGT

GCCACAGAAACAAACTTTTAACTTACAGGCTACAAGATCACCAAGAAAAGAAGTGCCAGCAAGTCCTGGC

CACAACAAAGCTCCATTCACCAAAGACAAAATATCCAGCCGAATTGAGGCTCGTCTCACAAGAGATGAGC

AACGTGCAAAAGCACTAAATATCCCTTTTTCAGTTGAAAAGATCATCAACCTTCCTGTGGATGATTTCAA

TGAAATGATGTCCAAGCATCAGTTCAATGAGGCTCAACTTGCTCTTATTCGTGATATCCGAAGAAGAGGT

AAAAATAAAGTTGCAGCTCAAAATTGCCGTAAACGGAAACTTGAAAGTATAGTGGGTCTGGAGCATGAAC

TGAATCAGCTTAAAGATGATAAAGAAAAGCTACTTAAGGAAAAAAGGGAGTATGATAAAAACCTCCGTCT

GATAAAACAGCAGCTTAACAGTTTGTATCATGATGTTTTTAGCATGCTGTGTGATGAAGATGGGAAGCCT

TACTCTCCTAGTGAGTATTCATTACAGCAGACAAGTGATGGTAGTGTATTCCTTGTTCCAAGGGTCAAGA

AGCCAGAGATTAGGAGAGAATAA

>mo_b_glabrata lcl|XM_013211185.1_cds_XP_013066639.1_1 [gene=LOC106055065] [protein=nuclear factor erythroid 2-related factor 2-like isoform X1] [protein_id=XP_013066639.1] [location=390..2813]

ATGATAAAAGAATATTTTACTGATGGCCTCATAGGGCTAGCAATACTTCTCAGTATATTTCGAACAGATC

TGGTTGGAATTAATAATCTAATTAACTATCCAGAAGTTCAAGAGATCATTCTAGGACAGACAGTGGCATA

TTTGCCTGCAAGCTATAATCACATCATCAACTCACATCATCCTTTTGAAAGCTCAAAGCATTTAGAGCTC

AGCAATGAGGCGTTTTCTGCATGGCATCTAAATATAAACAATTTGCCCTTCCTTAGAGAAAGGCACAGGA

CTGAAATAGAAGCCTTTCTAGTCAGTGGAACTCAGCAGAATGTAGAGTTGGAAAACTTTACTGGCACACC

CACTACCTTGAATGTAGAGCATGAAGATAATCAAATTGTGGATTTATCTGGTCAAAATCTACCAGAAGAA

AGTGCTAGTTCATATTCTGAGGCCACTATTTCAAATGATAATGTCCCCGAAAGCTCAGAGGATACTCTTC

AAACAGCCATTGCAGATACATCAATTGTAAATAATCCATTTCCTGGTTGTAATTTAACTAAAGAGGATTT

GGATTTAATAGATGTACTCTGGCAACAAGATGTTGATCTTGGTGTTGGCAAAGAGGTGTTTGACTTATTT

CTGAGGCAGGAAATTGAAACTAAAAAGGAGCAAGAATTACTCAAGCATCAAGAATGGGAAAAGTCCCAGT

TGCAATTGAGAGAGAAGCAAGAGAAAGAAAGACAAGAAGAGGCTGAAAAATGGTTGAAAGAAAATTTCAG

GAGAGATGGTGAAACTGGTGAGTGGATTCGTAATTCAAATGGACCATCAAGTTCTTTGGATTATTTTGAA

ACTGATGATTCATTTACCCTTGAGGCTGCTCTTGACTATCTCTCAACAAACATTGACCATGCTCTCATTA

AAAACTTGGATATTTCGCCTGGCATTATTCCAAGTTACTCAAGTGATCAGTTGACTGACAACTGTCTAAT

CGGTACTCCAGAAGGTCAGGGTTTTCAACAATCACAACAGCTTCTGTCTCACCAAGACAGTCTTGAGGAA

AGCTTAAATGGTCTTCTGGATTTTCTAAGCAATGAGCCTGTTGAGGATAACTCTGCTTCATTGGATCAGT

TTGGCATAGACTTGAACACTAGTAATCTAACATCAGACATGAAGCTAGACCAAGATTTGGATGCACAGAC

TGATTACCTTATTCAGAATGTTACTATGCAATCTCAAGAAATTCAGCCTGTGGCCAATGAGATGAATGCA

ACATTTCCTTTCCTGTCAACAAACAGTAGTGACCAAATGAATTTTGACTTGGACAGCCTCAGTTTCCTTA

ACAGTTTGGCTACAATGCAGAATGGTGGACTGGATGACGGTGAACTGTTTGAAAATATTCCAGTAGACTC

AAATGAAACCTTACTGAATGCTAATATACCTTTGAATGAAAGTATGGATACTTTTACAGTTCTACATGAT

AATTTTAGTCAGTCACTTGGAGCAATGGCAAGCCCTTCATCTTATGATGGACTGTGTGATTCTTTAGATG

GTCTTGAAGGAGCCATAGGAGGCTCAGATCTTAGTGCGAGTAGCCAAAACAATATCACAAGACCCTACTC

TAAAGTCAACAGACTTTCTGAATCTTCCAATGACTCTGGCTATCCCTTCCAGAGTGGATTTTCATCATCA

TCACCTGCATCATCCAGCGCTAGCAGTCCTGCAGGTTGCCACTATAGCAACATTTCTACTTCAGGCAACG

ACACTGAACACGATCCCCAAAACACTCAGACAGTGGCTCGCCATGCTGTAGCTCATAACCACACTTACAA

TACTCCACCTGGACAAGTGCCTAGAGAGGTAAAAAAATATGCTCCAAAAGAGCCTTCTAGAAAAGGACCT

CATAGCCGTGACCAAAGACGCTTGGAGGAATTCAAAATACCATATACAATCGATGACATAATTGAATCCC

CAGTAGAGACATTTAATGAAATGTTAAAAAGTCATAAGCTTAGTGAGGCACAGTTATCTCTTATCAGAGA

TATTCGACGCAGAGGAAAAAATAAAATAGCTGCTCAGAACTGCAGGAAACGAAAAGTGAATGTCATTGTA

AATTTGTCTGATGAGATGGTGGACTTGGAGAAAGCTAGAGACAAACTACTCAAAGAGAGAGCAGAGATTG

AGAAAGAAACTCTCAAGATGAAAGAAAAGTTTGGGCACTTGTATACCCATATATTCCAGTCACTGAGAGA

CGAACATGGACAACCTTATGACCCCAACCTCTACTCCTTGCAGCAGACCAGTGACGGAGATGTGTTGTTA

GTCCCACAGAGCATGAATAGAAACAAGTACAGCAACTCTTCTTCGGCTTCATCATCTCCAACATCTTCTA

AAGATTCTGTCAATTCAAAGAAACGCAAATCCTTTGATGAGTAA

>hc_s_kowalevskii lcl|XM_002737074.2_cds_XP_002737120.1_1 [gene=LOC100369481] [protein=nuclear factor erythroid 2-related factor 1-like isoform X1] [protein_id=XP_002737120.1] [location=176..1966]

ATGACGACAGTTCAAGTAATACCAAGTCCGGCAAAGGACATGGATCTCATAGATGTACTGTGGAAGCAGG

ACATTGACATGGGTGTGTGCCGTGAAATCTATGATGGAACATATCGACAGAAAGAACTGGAGAAAGAGAA

GCAACTTGAACTAGCAAAACTGAAAGAAGAAAAGTCACCGAGTATTGGTGATGAATGGTCTGGATTGGAA

TATGGCGTTGACAGTGAAACTGGGGAGTATTATCTTATCCCACATGTGCATGAGCCAACATCAGAACCCG

AAGCCACGGCTGCAGACATCCCTCTTGATCCAGATGCAATTGATTATAATCTCGATGAGTGTATTCAGAT

GTTGCGCGAGCATGCACTTCAACAGAATGGTCCCCAGGCTGTGGACGACGAGGGCCTGCCCCTCTCTGTG

GGTATTACCAGTCCACAGATCCTGCCAGCAGAAGATAATGCCACTGCTGAACAGCAGTGGCAGGACCTGG

CTAGTCTTACCGAGTTACAGCTTGGGTTGCCTCCGTTGCCACCACAGAATGACACGTATGCTGTAAATGC

AACAACGAATGGGAACGTCAATCTACAGAATGCTAGCATGTCACCGGAATTGAACAATCTCACTGACTTC

ATGTCTGTGGGTGCATCGCTGCTTTCCCCAGTGGTACAAACACCAGAAGACTTCACCTACAACGCCAACA

ACACCGATTCCTTACTAGCAGCTCTGCTGAGCGGTGCAGTGATCGGAGATATTGACCTGATGAATGACAT

AACCATGGACGAAACACTGAGTGCATCGCTGGATGACATTGATCCATTAAACGCATCTATGGAAGATGAA

CATGTTCCATCAGAGAATGGTGAACTCACCACATTACTGCCTAGTGTTACAGATGATGCTGATTCTGCCG

TATCTGTTAATAGTGGAAGTGTTAGCAGCGGAATTAATTCTCCGTACAGTTTTGATAATGACTGGAATGA

CACCACATCATCACATTGCAATGATACCAGTGATGACGGTATCTATGACGACATGGAAGGAGCTACCGCT

ATGGATTATGATAGCGCTGATGATGATATGAATGCATACTTTGCCGGTAAACAAGTCGTGAAAGAAGAAA

GTCAGTACGATAAATACCAACGTTTGAGTACGCAGCCACCGAACATGGATAACATCAAACACAACCACAC

CTATCCACAGCCCCACGACTCGGAACAGAAACAACAAAACAATAAAGGCAATCATAATACTCCGGGCTAC

AGCGGTTCCTCGTCTAAGAAAGACAAGAAGCACAAGCTGTCGCGCGATGAGAAACGTGCCAAGGGTCTGA

AAATACCGTTCACCACTGATAAAATAATCAATCTCCCCGTCGACGATTTCAACGAAATGTTATCAAGTAG

CTCCCTGTCTGAAGCGCAGCTAACGCTGATCAGAGACATTCGCAGACGTGGGAAAAACAAGATAGCTGCG

CAGCATTGCAGGAAGCGGAAACTTGAATCAATCTCAAACCTGAGTGATGGCTTAGCGGAGCTGAAAGCAG

AGAAAGAAAGGCTATGCAAAGAGCGGCGCATGATTGATAAAGAAACCATCAGTATGAAAGATCGGTTTCA

AGTTCTCTACCGGGAAGTGTTTGAGAGTTTACGAGATGAGAGGGGAGCACCATACGACCCGGAAGAGTAT

TCATTGCAACAATCTACAGATGGTAACGTCTTCTTAGTACCAAACACTGCCACTCAAAGACAACAAGAAG

ATCGTAAAAACAAAAACAGAAGAAAGGGCCGCTCCAAATAA

>ed_s_purpuratus lcl|NM_001129806.1_cds_NP_001123278.1_1 [gene=Nfe2] [protein=nuclear factor, erythroid derived 2] [protein_id=NP_001123278.1] [location=81..1898]

ATGGATGTGGATTGTGACGAACTGGGCATTGCCATGGTCCCTCAGAAAACACTACCACAACTTGACACTC

AAGAGGACATGGACTTGATTGAAAGCCTATGGAGACAGGATATTGATATGGGAGTTTGTCCTGAGACCTA

CCCTTACAAGACAGACCTCAAACCAGGCTTTACTGAGGACCTCAAGAAACAAGGCCTAGACCAATGGGAT

TACAACTACAACATAGATGGAGAGACAGGAGAGTATGTTGCTGGTCCTCGACAAGACTTTGGAGAAGAAG

TCCAACGAACAGTCCAACCTCAAGAGCCACTTCCTTGCTCTCAGCCCCAACAAGACGCACAGCCTCAGCA

AGATAGCAGTCTCTCATTGGAGGACTGCTTACAGCTCTTAGAAGACGAGTATTCACCAGACACAGCACAA

ACTGAACTTTCCCCTGGGCTTAGTCAGCAAGAAACAGAACAGCGATGGCATGACCTTGCCACTATTCCGG

AACTTCAAGGTAGTATCCCTGAGCTACTGCCAACCACCCCTACTCTGAACAACACACAAGAAGGGTACTC

CACTCCCCAACAGCTTTTTATCCCACCAGTGGCTAATGTCACTGGAAGTCTGGTTCCAGAGGTCACTGAA

CTGTCGCAACCTCAGCTGTTGAATGATGTCTTTGCTGTAAATGCTTCCACCAATGGGATGGTGAGCTTGC

AGAATGCCACCCAGTCACAGCAGCAGCCGATACTTTTGCCTCCCACACGCAACGACTCATTTAACAACTT

TCCAAACGTGGATGCGCAGAGTCACTCTACAAATGCCACTGCAAGTGCTCCTAATGACAACATGACAGAC

TACTTGATGCAGACATTGCAGCACCAGATGAATGCAGTGAATGCCTCAGCACCTTTCCCTGCTGCCAACC

TCAGTACAGCAGAAAACGGCAGCTCTCTCCTGATGGATCTCCTCAACTCACCAGGCTCAGCTCATCCAAT

CAATCCATTGGATCTGGACTTTGATGAACAGATGCATGATGTCATTGCTGCTCTTGGAGAAGACTCTGAG

TCCCTGAGTAATGAAGAAGATAGCGATGGATCCCTGTTTGAAGCTGAGGGTGCATCAGGCTACTCATCTG

GCTCAGATGAAGATGACTACAAGGGTGCTGCTGGAGGATATGGTTTCTCAAGAGGTCATGAGAGGCAGTA

CGGGAGTGATTCCAGCAATAACAGCTACAGTGGTCAACCCCCTAAGATGGAGAATGTGAAGCACAACCAT

TCCTATGCTGCTCCCTCACAGTCCCAGATGCAGAATGGCAATGGTGCACAAAACATAAGCTTCAACGGGA

CAAACGGCAACTACATGAAGTTAAACCGTGACGAGAAACGTGCCAAGGCCCTCAAACTTCCAGTCTGCCT

TGAAAAGATCATCAACCTGCCTGTCGATTCTTTCAACGACCTGGTCAAGAAATATGAGCTCACAGATCCC

CAGATGCAGCTCGTCCGTGACATCAGGAGGCGTGGAAAAAACAAAGTTGCCGCTCAGAATTGCCGTAAAC

GTAAAATAGATGCCATCCAGATCGTGGAGTCAACGGTTGGTGAGCTAAGGATGGAAAGGGACAAGTTGGT

CAAAGAGCGTGACAGCATTGACAAAGAAGTCAATGAGATGCAGCAGAGGTATGCAGAACTCTGTGAAGAA

GTCTTTGCCTCGGTTCAGGATGAGCATGGTAGCCCAGTTGATCCCAATGACTATAACGTACAACAGATGC

CTGATGGTACTGTCTACCTTGTCCCTCGCAATAGCAATCAAAGAGACAGTGATGAGCAGTCCATGTAA

>ur_c_intestinalis lcl|NM_001078302.1_cds_NP_001071770.1_1 [gene=nf-e2] [protein=transcription factor protein isoform 1] [protein_id=NP_001071770.1] [location=143..2911]

ATGCAAGTTATACGGAATATTCCGGAAAAGAAAAACTTAGCCTTTGGGCTTTTAGACATTGGGATTTGTT

TAGCTATTTTACAGCTGACAAATGTAAACAACCCATCCGACAATGAATCGTATATTGAAGATATTTTTAT

TGGGGGACCAATGGTTGGTATTATCCCATATCAACCACTTCAACAAAGACTTCATTTCCAAGATATGAAA

GTGTTGGATGATTATGAATATGCGATGTCAACATACAGATCTATAATTCGGGATGTGCAGACGCGAATAC

CCACTGCAAGTGTAAGCCAACAGCAAAATATTAATAACGTCACTGCAATGATGCTGAGAGTTGGAAGGAC

CTCAAATGCAGTTGAACGTAATTCTGATACTGAACATAGAAATTCTAACGTGTCAAGTTTTCCTGTTCTG

GATGAAAACAGAGAAAGTTCTGGTTTAAACTACACTGTGCCTCGGTTTGATGTATTTAATGAAACTCAAC

AAGATGAAATAGACACAAACATTGTGGATAATGATGTTTTAACACAGTTGTCACAAAACCTTGATGATGT

GAGATGGCGATGCTACATCAATGATCCATCTGTTCGAGAATGTTTAACTCTTCCTATCATGCCAAGTTCC

AGTGCTCCACTGAATTGTAATGAATCAGCCGAATATTATGAAGGAGTTCAAACTCATTTAGTATTAAGTG

AAGAATTTGGTTCGAATATTGGAATCCAAGAAAGTGAAGACTCCGCATCTGACTTTAATCCTTCTCCAAT

TGTATCTAACCAAAATACTGTAATGGATACTTACAGCAAAAATTCACTTGAATATGAACCTGAAGCAACA

ATGTCTTTTGATTACAATAGTCAGATAGATGTCACATATTTAGAGGATTCTGACATGATGCAATTGATGT

ATCAACAAGATGTAGATCTTGGATTCAGGTGGCCATCAAGGGATCCTGTGAAAAACTTGGACGAAATCGA

TGAAGTTTTAAATATGAAAAAAACAACAGATGTCGGTGAATTCTTTGTGGATGGTGAGACAGGGGAACAA

ATCCCGATTTCAAAAGTGCAGAACAATTCAAAACAACAAGAGCTTAATGATAAAGTTGAAACATCACAAG

CGGTGGAACCTTCTACTGAACCTACATCGAGCTACTTTAGTATTGATGAATATCTTGAAATATTGAATGA

ATCTATTGATGATATACAACCACAAGTTGTTGAACAAACTAATTTAAATCAAATCTCGGAAGAATCTATT

ATCATTGAAGATTTAACTGATCTTCTAACAGATAATGAAAACGGTAGCACAATGGAACAAAATAATGCAC

ATTTATGGCAAGATATGTCTGCCATTCCACAACTACCTGGAAAAAGTTCAGAAGCTGAGGGGCAACTTGG

AACAGTGAATGTTATAAATACCAATGTCAGTCTGACAAATGCAACTGTGGAATCGAATCAAACAAACGAA

AATACTCTTCACCAACAAGGAACAAGAAATATAAACTTAGGATACAAACCAGTTGCCTTTGAAAATGAGC

AACCATTTGCTACACAACCATACAATGTGTACAAAACACCAGCATTCAATCCAACGGTTCCCTGTAAACC

AGCTTTTTCATCACCAAGTTACAACACGTCATTGAATGGTGAAGATGGAATACCTATGGATGGTTGCCTC

TCACTACCCATTGTGACATCACCGCAGGGATTGTTACACAATCAAATAAACAGTTATCAGGCACCAATGG

AGACGGGAGATTCCCCACCATATGATGAAGTCAGTCCAGTGTTAAGTTTCCATGGAGAACCAGGTTCTAT

GTTCTTTGATGAAGCAAATGTGAATGAGATGCTTGATGACATCATCAATATGAACTCAATGAACCAAGTT

TATAATAATAGCACGGAAAGTGCTTATTTGAACATGAACCCTGCTGTGTTTGTTCCACCACCATTGCAAG

CCAATCCACCTCAGATCTCATTCAATGATACAAAATCACTCGATGGCAGCACAAGCGACCAACTAAAGAT

TTCTGAAACTTGTGATTCAGATTCTGGTGTTTCCATGAGTCCACATTCTTTTTACATGGGACATCAAAGA

ATGGAAAGAGATAGCATTGATAATGAAACATCTTTACACGAAACATCGTTTGCAACAAACGCATTGAAAG

TGATCAACCATAACCACACCTATGATGGCACTGTTGGAAAACCAAGAATCATAAAAAAAAAACCTGACAA

ATCTGCTTCTCATATTCGTGAATCTCGTGACGAGCGCAAAGCTCGTGAGTTAAACATCCCATTTACTCTG

GATGAGATCATCATGTCACCAGTGGAAGAGTATAATGAAATGCTTGCTCGAACTCCTCTTACAACTGCTC

AACAAACATTAATCAAAGACATTCGAAGAAGGGGGAAAAATAAGGTAGCAGCACAGAACTGCAGAAAAAG

AAAGATAGAAACCATTACTACAATGGAAGAAGATGTGGATGTTCTTCGTGGAAGGAAAAATGATCTTGAG

ATGGAACAAGATGAACTGGAAGCAAGAAAACAAAACCTGAAGTCACAATACAATGCACTATACCAGCAGA

TATTTCGTTCACTTCGTGATGAATCCGGTCGACCATACGACCCATCACTCTACACACTTGAGCAGGTAGA

GGGCGCTGTTTTGCTTGTTCCACGAAATCTTCGTAATCGTGATCACAACAACTCTGATCATGAGGATCAT

GTGGATTTCACAAGGGTAAAGATGGAAAAAAGAGATTAG

>ar_m_occidentalis lcl|XM_003741108.1_cds_XP_003741156.1_1 [gene=LOC100906298] [protein=uncharacterized protein LOC100906298] [protein_id=XP_003741156.1] [location=1..2199]

ATGTACCACGTATCTATTGGGCTTCTTCGAAAGAAACCCTTTTTGCATTCGCACCTTCTGCAACTTCTGT

TGGCCGTTGGACTTCTTCGCTGGTCGGCTCCGCCAGAACCATGGTCCCCCCTGTACATAAGCAACAACCC

ATCGAGCGGGAGCCTCGACGCGTTACAGTTGGCCCCTTGGGAGACGTATGCACCTCAGGCGTCGCTCATC

CACCCGAAAGCTCTGCGGCACGACGATGGCTACTTCGACCTGTTGGAGACCTTTTGCGATTATGACAGGT

TAGCCAGGGCGGCCTCAAGAGGTCCTCTGATCGCGTATCTTGCGGAGGACGGTGCGCCATCCAGAGCTCC

TCACGCATCCACGGGCTCCACCGCTCTCGAAGAGTGTCAAGCCGAGACTCTCAGTAGAGAAGACGCGGAT

CTGATCGAGATTCTATGGAAACAGGATGTCGATCTTGGGATCCCTCTCGAAGACTATAGACCGTGCAATC

AACCAGCGTCGCAGTCTGTGGCGGCAGCATCGCCTCCCCCCAAGTCCGTCGAAGGGAGCACGGTCGTGCC

GGTTCTCGACACGTTGGAATCGAAGAAAGACTTTGAACCTTCAACCAACGAGCCCCGCGTGACGTCAATT

GACTCAGAAACCGGCGAACCCATCTTCGAACCTGTTGCTGGACCTTCGACGCTCGATAGTGTGCTTCAGA

ATAACTCGTCGTCCGATTCGTTCCTGGATATAAACGCTCTCATGGGTGTTGTCAGCGAGGAGTTGTTCTG

TGCGGATCCTGAATGGGCTCAGATGCCTAGCTACTCCGGTGTTTCGAGTCCCCCCTTCAATGGAACGGAT

CCTTTCGGAGGAGTTCTCTTGCAGAATGTCTCGATGCCGTCCTCATACGACGCGTCCGTGACCGCTGTGA

ACTCGACGATGCAACAAGCTTCACCGGCTGCCGGCTCCACATTCGCGAGTACTCCAACCGCTGCCGCTTC

AGGAGACGCTCCCGTGGGGTGCAACGGTTGCAGTGATTTGTCGAAGCCAACGGTGACCGATCAACACGCT

ATCCGAGATCTCTTCACTTACAACAACTCAACTCAGAGCGGCTGGGATTCTTTCGAGCTATTCTATGAGG

ATCTGGCGGCTCTCGCATCGAACACTACAAATGCCACGAACAGTCACTCCGAAAGCAACCGTCTCGTTCC

TCTTGGACCCACGACCGTCAGCCAACCCGCGAGTAGAATGAGTTCTTCATCTGCGAATGGCGGAAGTGTA

TCCCTCAAACACACGAGCATCGGGGACGGAGGATACGCCGCTAGCGATACCGGAGTATCGTCTATGTACT

CTGACGAGGCGAACGAGGAATGGATGGAGAGTTCTAGCGAAACTTCTCACGACCACGACCAGGAACAATT

GGTGTCGACGGACGTCAGTTACCACTCGAACTCGTCATCGATGTCGTTCGGTTCCGCTGAAGGGTGCACA

GTCCCGCAGAAAAAATACAATTTCTTCGGACGAAAACCCTATCACCAGACTTCCAACGGCACGGTGGACC

GCTCGATCGAGGAGAGTCATGTCGTGCCCCAGCCTCTCAAAATCGCCCACAACCCAGCCATGGACGGCTT

CCTCCACAACCATTCGTACGGCCAAGAGCAGCTGAGCGCCGGTTACAACGTGCCGCAGTTGCCGGTCGGG

GTGAAACTCGAGACTCAATTCCACTCCGAGCCGAGGAAGCAGAGTTCCGCGTCTCAGGCCGACGGCTACG

AATCGTCCGACCCATCGGAGTCTGGCGCCGGATACATCCAAGTGACGTCGGCCAAGGACGAACGCCGCGC

GCGGGAACTGAAGATCCCGATTCCGACCGAAGAAATCGTGACCCTCTCGATCGAAGAGTTCAACGAGCGT

TTGACTCGCTACGAGTTGAGCGAAGATCAGCACGCCCTCATCCGCGACATCCGTCGCCGAGGCAAGAACA

AGGTGGCTGCACAAAATTGCCGAAAACGGAAACTGGATCAGATCTCTGCTCTGCAAGACGAAGTTGAGAA

TTTCCAGGACACGTGTCGCTCTCTTCAGAACGAAAACGATGAATTGACCCGACGCGAACTCTACGCTCAG

CAGAGGCTGAACCAGCTACGAGATGTGATAGCCAACGCTGCGAATTCCGGCGCGATTCCCAAACACCACG

GCCACCATAAAATGGCGAATTCGGAGTAG

>i_dp_a_aegypti lcl|XM_001650266.1_cds_XP_001650316.1_1 [gene=AaeL_AAEL005077] [protein=AAEL005077-PA] [protein_id=XP_001650316.1] [location=1..1890]

ATGGAAGTGCGCCGGTATGCTGGGTTCACGGATGAACGCTGGAGCTGGAACTGGCAGAAGAACGCGTCCC

GCGTTCCGCTGAGCCGGGCCGTTTCGATGGAACAGCGCTTTCAGGATCTGGCCAATCTGCTCAGCTTTCC

GCCCGGAATGGGCGTCGGCATGGGGGTGGGCGAAATGCCTCCGGCCCATCCGCACCCGCACTACCCGCCG

CACTACTCGTACCAGGCTAACGGTGCCATTCCCCAGCACGGTCAGTACCACTCACATGCAGTACTGCAAA

ATGCTTCGCTGGCGGATATTGGACCGACCCAGCCGTACTATGCACCGAACCTGGGTTCGGCCGTGGCCAC

CAGCATGCACTTGACCAACTCGACGTCGGAGACGGACGCCGGAGCCACCGGGTACAAAATGGATCACGAG

ATGATGTACTATTCGAATACATCATCGGAGATGAATCACACCGACGGGTTTCTCAACTCGATTCTGGATG

ACGACCTGCAGCTAATGGACATCGCAGTGAACGAGGGTATGTACACCATGCGCATGCTGGACCACAATGC

CACCAGCAGCAATTCGTCCGTGCTGGGCGGTTCTCTCGGAGGAAGTGCTGCCGCTGTTGCCGCAGCAGCC

GCCGCCGGTCTGAACGGGCCTGCCCATCTCGGTGGTCTGATGTCCAGTGCATCGGCTGCCGTCTCTTCTG

GTGCCATGCAAACCTCCCTGAACGGTTCGACGGGAACACACGGAGCCACCGGTGGCACCACGAGCGGTGA

CCGCCTAGATGCCTCCAGTGACAGTGCCGTATCGTCGATGGGATCGGAACGGGTTCCGTCGCTCTCTGAC

GGCGAATGGGGTGACGGTGGAAGCGACTCTGCCCAAGAGTATCACAACAAGTATGGAGGACCGTTCGATT

ATAGCTACAGCGGAAGCAATCGGCTTGGCGATGGAACAAGACAGCCACCGGTTGCCCAGAAGAAGCACCA

CATGTTTGCGAAAAGATATTTCCAAGAACAGAACACCTCGATCCCCTCGCTACCGTCAGCTACCAATCCC

TCCGCGACAGGACCGACCGTTGATCCCCAGAGCCAACTGAATGCAAGTATACCAATCAAATATGAGTTCG

ATTACATGAACCCGGCTTCGCTCAGTCACTTAGAGGGAGCCGTCGGCCCGGTCACCAAGCAAGAAGACCA

AACCAGTGCCCATAATAACCCACTCTCCTCGGTGGACATGAAATACCCGTACTCATTGGATTTCTCCCGG

CAGAATCCTGCCTCGGCACCAGCCGCCCGAAGCCATCATCACGACGTGATCCACCATAACCATACGTACA

CCTTGCCGCACAATAGCGGTGCCAATCCAAAGCCCCAAACCAGAGACAAACGCATCCGGAAAGCCGAGGA

GGAGCACCTGACTCGGGACGAGAAACGTGCCCGTGCGCTCCAGATCCCCATCCCCGTGCAAGACATCATC

AATCTGCCGATGGACGAGTTCAACGAGCGCCTGTCCAAGTACGACCTGAGCGAAACGCAACTGTCCCTGA

TCCGGGACATCCGGCGGCGGGGCAAGAACAAGGTGGCCGCCCAAAACTGCCGCAAACGCAAACTGGACCA

GATTGTGACGCTAGCCGACGAAGTCAAGGACATGAAGATGCGCAAGGAACGGCTGTTGCGCGACCGCGAG

ATCATCCAAACCGAACGCAAGAGAATACGGGACAAGTTCTCGGCGCTGTACCGCCACGTGTTTCAGAATC

TGCGTGACAGCGAGGGTAACCCGTACTCCCAGGAGCACTGGAGCTTGCAGCAAAGTGCCGATGGAACGGT

TGTCCTGGTGCCCCGAAGCGTTGACCGTCAGCAAGATCTGACCGACCGAAAGAGTGAGACCGGTCCGTGA

>i_dp_d_melanogaster lcl|AF070062.1_cds_AAC72896.1_1 [gene=cnc] [protein=cap 'n' collar isoform A] [protein_id=AAC72896.1] [location=476..2077]

ATGGTTGACAACAGCACTAGCAACAACTCCTCGGTTCTGGGCTTGCCCAGCAGTGGACATGTTAGCAACG

GCTCCGGTAGCTCGGCACAACTTGGGGCGGGAAATCCGCACGGTAACCAGGCCAACGGAGCGTCCGGCGG

CGTTGGCTCAATGAGCGGATCAGCTGTGGGCGCTGGAGCTACGGGAATGACCGCCGATCTCTTGGCAAGC

GGCGGTGCAGGAGCACAAGGCGGTGCGGATCGCTTGGACGCGTCCAGCGACAGTGCTGTCAGTTCGATGG

GCTCCGAGCGAGTGCCGTCCCTCTCCGACGGCGAGTGGGGTGAGGGCAGTGACTCCGCCCAGGATTACCA

TCAGGGCAAGTACGGAGGCCCCTACGACTTCAGCTACAACAATAATTCACGGCTTAGCACCGCCACACGT

CAGCCGCCGGTGGCGCAGAAGAAGCATCAGCTGTACGGCAAGAGGGATCCCCATAAGCAGACGCCATCGG

CTTTGCCACCAACAGCTCCACCAGCAGCCGCGACTGCAGTCCAATCGCAGAGTATCAAGTACGAGTACGA

TGCTGGGTACGCCTCCTCGGGAATGGCCAGCGGTGGAATCAGTGAGCCAGGAGCGATGGGACCCGCTCTA

TCCAAGGACTATCATCATCATCAGCCTTACGGCATGGGAGCCAGCCGCAGCGCCTTTTCCGGCGACTATA

CAGTACGACCATCGCCAAGGACTTCGCAGGATTTGGTGCAACTAAATCATACCTACTCGCTACCCCAGGG

AAGTGGATCCCTTCCCAGACCCCAGGCACGCGATAAGAAGCCCCTGGTCGCCACTAAAACCGCATCGAAG

GGAGCGAGTGCCGGCAACAGCAGCAGTGTTGGCGGAAACAGCAGCAACTTGGAGGAAGAGCATCTGACAC

GCGATGAAAAGCGCGCTCGATCCCTGAACATACCCATTTCAGTGCCGGACATCATTAACCTGCCCATGGA

CGAGTTCAACGAGCGCTTGTCGAAATACGACCTTAGCGAGAACCAGTTGTCGCTGATTCGCGACATTCGT

CGGCGTGGAAAGAACAAGGTCGCTGCCCAGAATTGCAGGAAACGCAAATTGGACCAGATCCTGACTCTAG

AGGATGAGGTGAACGCGGTGGTTAAGCGCAAGACCCAACTTAATCAGGACCGCGATCATTTGGAGAGCGA

ACGCAAGCGCATCTCGAACAAGTTTGCCATGCTGCATCGTCATGTCTTCCAGTACCTACGGGATCCCGAG

GGAAATCCCTGCTCGCCGGCGGACTACAGTTTGCAACAGGCTGCCGATGGCTCCGTCTACTTGCTACCAC

GTGAAAAGTCCGAGGGTAACAACACGGCTACGGCTGCCTCCAATGCTGTTTCGTCGGCCAGTGGGGGAAG

TCTTAATGGCCACGTGCCCACTCAGGCTCCCATGCACAGCCATCAGAGCCACGGAATGCAGGCGCAACAT

GTTGTCGGTGGGATGTCGCAGCAGCAGCAACAGCAGTCGAGGCTGCCTCCACACCTGCAACAGCAGCATC

ATCTGCAGTCGCAGCAACAGCAGCCGGGAGGTCAGCAGCAACAGCAGCACCGCAAGGAATGA

>i_ho_a_mellifera lcl|XM_003251740.2_cds_XP_003251788.1_1 [gene=LOC725081] [protein=segmentation protein cap'n'collar-like isoform X1] [protein_id=XP_003251788.1] [location=907..3837]

ATGTTGTGTATAAAGAAATTGTACCACGAGGAACTATTCCAATTAACATTGTTACTTTCTTTATTAAGAA

TAGATCCTGAATCCTACCTTGGTTTGGATATTCAAACCATAGGTGTAGGCTCTTTAGATCTAAATAATGG

TTCAAGATGGCATACTGATGTTCATACAATAGTTCATCGTCCTATCTTTGTTCATCCTAAAAACTTAGAT

TCTATGCTTTTAAACTATGAGAGAGATTTATTCGAAGACTTGAATTCATTAGGAAGATATAATAGAATAA

ATTCTGGTTTAAATGATATCCATGCATATTTGTTAAATGTTGAAGAATCTACAAGAGATATCGCTATAGC

AGGACCTAGTATATCACTTTCCACAGACCCAACTAGGAATATGACATCACCTGATTCATCAAATTCTTCG

CAAAATCCAGACGAACCAACTAATACGGCAGAACTTACTCAAGAGGACATGGATTTGATCGAAGTGCTTT

GGAAGCAAGACGTGGATCTGGGATTCACGTTGGTGGAACCGACTACCACGGCGACCAAGAAGTTGTCCAC

GGTGGAGAAGGGAAGCGACGATGAGATCGAAAAATTAAAAGCTTTAGAAGCGATCAATGGAAGCAACGAA

GAGAAGGATACAAAGGAATACGACGAAGCGCAAGACGATCCATGGGCAGGCCTCCCTTACACCATTGATC

TCGAGACCGGTGAATATATACTCAATTCTGGAAATCAAGAGGGAGACGGGAACAACGCGATCGAAGAGGA

CGATCGCCTCCTTAGAGAAGCGTCGCTAGATTTAGACAATCACCCGTTGGCTGGATTAACCGATGATTCT

TTGGGATTAACCGACACTTTAGAACTCGAGAATGATCTTCCTTCGGACTTGCTCGGAGGTAGTCTCTTAG

CGAGCGCAAACGTCGAAAGTCTTCTTAACAACGATAGTTTGGATCTACCCGACGGATTCAATCTCGAGGA

GGCACTCCAACTCGTTGGCCTCGATGAGGCGCAATCAGAGGAAACGAAACCGGAAGTGAAGAAGAAGGAA

AAAGATAGCATCGAAGAATCGACGAGCGAAGCAAAGGACGACGAGTCGCCGATTATAAGCAGCTCGAGCA

GCGTCGAGGTCGCGAAGTCGTCGAGGTGCGAGGATCCTGAGACCGGCGACATGATTCACACACCGCAATT

TCATCATCCCCATCATCCTCACCATCGTTCCTTCCAGGGTCGCATGCCATTCATGCGTGCAATGAGCATG

GAACAACGGTGGCAAGATCTGGCATCTCTTTTATCGCTACCTGGTGCACCAGAACATTTTCCACATACAC

ATCCTGGATACCCAGGGCACGGTATAAGCCACAGTCATTATGAAGCACAACGTAACGTGTTGCTTCACAA

TGCCACTCTGGCTCCACCGGTGGGTGATCTTAATTCGACCAGTCCTTACCACAATGTAGGTGGATCGTCG

AATTTGGGCTCGGCTGTAGCAACGTCGATGAATTTAACGAATAGTAGCGAACCGATGGGTGCGGAAAGTG

GAGCAGCTTATAAATCCGAGCCGGCAGATATGATGTACTATCACACACCAACATCCGATTCGATAAATCA

AACTACCGATGGTTTTCTTTCGTCTCTACTTAACGACGAGGATTTACATCTGATGGACATGGCTATGAAC

GATGGCATGTACACCATGCGCATGCTAGATAATGGTAATAACAACGCTTCTGGTCCAACGGGAGCAGCAG

CCTTGTCAGGAGTTCAGACAGCTGGTGGTACAACCTCTTCGGCAACAGGAGTTACGACACTTCCGGGTGT

AACGGATGAAAGGATGGATGCATCGAGCGATAGTGCTGTCAGCAGTATGGGCAGCGAACGTGTACCATCC

CTTTCGGATGGCGAGTGGATGGAAACTGGATCTAATTCCAGCCATACACAAGCGGATTCTCATTACACTA

TGGATTATGCTAGCAAATACCGCATGTCGTACGATTGTAGCTATTCCGTTTCCGGAAGAAATGCTGGCTC

TCCGAGATGTCAAACGGAACGTACGATGCCTCCGGTTGCTCAGAAAAAGCATCAAATGTTTGCAAAACGA

TATTTTCAGGAACAAGGAACTGGTTCGCCACTCGGAGCTACAGCGCATCCGACAACTCCAATGAAATACG

AATATGATTCTCATACAGTTGGTGCTGGAGCACCTGGAAATGCTTATTCCGGACCGATCGAGGGTGCAAC

TGGACCTCAACCTGAAATAAAATATAGTTGTAGTGTAGATTTCAGTCGGCATCAATCAGGACGTTCAGCT

ATAGAACATGTTCATCATAATCATACGTATCATTTACCTGCAGAAAGTTCTGGATCTCTTCAGCGTCCAG

TTTCTCGTGATAAAAAAGTGCGTAAAAACGATGGTGAAGAGCATCTAACTAGAGATGAGAAAAGAGCAAG

AGCATTGAACGTTCCTATTCCGGTGAACGATATTATTAATCTTCCAATGGATGAGTTTAACGAACGTCTT

AGTAAATATGATCTCAGTGAAGCTCAATTATCTTTGATACGTGACATTAGAAGACGGGGAAAGAATAAAG

TTGCCGCCCAAAATTGTCGCAAGCGTAAACTAGATCAAATCATTAGTCTCGCTGATGAAGTGAAAGAAAT

GAGGGATCGTAAAATGAGGCTCGTTCGTGAACGAGAATTTATGCTTATAGAGAGACAACGCGTGAAAGAT

AAATTTAGTCAACTTTATCGTCATGTGTTTCAATCTTTGCGCGATCCTGATGGCAATCAATATCATCCGT

ACGAATATAGTCTTCAACAATCTGCCGATGGAAATGTATTGTTAGTGCCAAGGAATCAGACGAATCCTCA

TCATCCCCGTTCTACGACAATGGAACCAAAAACGAAACCTGATCCTGAACATAAGGAATGA

>i_ph_p_humanus lcl|XM_002431067.1_cds_XP_002431112.1_1 [gene=Phum_PHUM512570] [protein=hypothetical protein] [protein_id=XP_002431112.1] [location=1..2046]

ATGAGGAGAACAGGATGGGGGGAGGGGAGCTCCTCGAATTTGGGTTCGGCTGTGGCGACTTCAATGAATT

TGACAAACAGCAGCGAACCTATATCGGATGGTAGTTCCGTTTTCAAAATGGAAAATCCTCACGATTTGAT

GTACTATCAGAATTCGACTTCTGAAATGAATCAAACGACCGAAGGATTTTTATCCTCCATATTGAACGAC

GAAGATTTACAATTGATGGACATGGCCATGACGGAAGAAAGGAACGAGGAGGATCGCATTGGTCACGCGG

GTCGGATGCCTTTTGTTCGAACCATGAGCATGGAACAACGTTGGCAAGATTTAGCCAATCTCCTAAGTCT

TCCGGGACAGGAGGGAGTTGGCGTTCATCATCCGTTCGCACATCATTCACACTCGCACCACCATCATCAT

CATCATCATCACCACGGAAATTATAATGGTCATAATTATTCGCACGACGGACGAGGGGTTCTCATACATA

ATGCGACCCTTGCACCACCCGTTGGTGATTTAAACGGATCCGGTCCGTACAACAACAGCAGCACGACGAT

GGGGAGCTCTTCGAATTTGGGTTCGGCTGTGGCGACTTCAATGAATTTGACAAACAGCAGCGAACCTATA

TCGGATGGTAGTTCCGTTTTCAAAATGGAAAATCCTCACGATTTGATGTACTATCAGAATTCGACTTCTG

AAATGAATCAAACGACCGAAGGATTTTTATCCTCCATATTGAACGACGAAGATTTACAATTGATGGACAT

GGCCATGACGGAAGGCATGTACCCGATGAGAATGCTCGAAAGCAACAGTTCACATAATGGAACAACAACC

GGAGGACCCCCAGTGGGTCACACGGAAGAAAGATTGGACGCTTCGAGCGACAGCGCGGTGAGTTCGATGG

GATCCGAAAGAGTTCCTTCCCTCTCCGATGGGGAATGGATGGAAACGGGTTCAGATTCTGGACACAACAC

GGGTGATCATTACGGAAACGTTGATTACCACGGAAGTAAATTTAGACCGTTCGACTACAGTTACACGGGG

AGACCACTTTTAGGTAATACAGGTCCGCAATCTGCCACTCCCGACGGTCACATACCTCCGGTGGCACAAA

AAAAGCATCACATGTTTGGTAAAAGGTATTTTCAAGAACAAGGAAACGCGACCACGGGTTCGACGTTGCC

TCCTCACAGAGCTCTCACACTATTACCGACGGCAACGCCAACACCGGCTCCCGTCAAATACGAATACGTT

GAAACAGGAGCCGAAGCCATTGTGCCTCCCGGATTTAACAATACGGTAGAGCCGTCTTGCGGAAATAAAA

TGCCGGAAGTGAAATATAGTTGTAGTCTTGATTTTATACGCCATCATCAAACGGGTGCGAGATCTTTGGA

ACACATTCATCATAATCACACGTATCCTCTACACGCGGAAGGAAGCGTGTCCATGGCGGCGAGACCGTCA

CACAGAGAAAAAACAAATTCTAGGGGACGTAAATCCGAAGAGGATCACTTGACGAGAGATGAAAAACGAG

CCAACGCAATGAACATTCCCATGCCGGTAGAAGAAATCGTTAATCTTCCAATGGACGAATTCAACGAACG

TTTGTCCAAATACGATCTTAGCGAAGCTCAATTATCACTTATAAGAGATATAAGGAGAAGGGGTAAAAAC

AAGGTAGCAGCGCAAAATTGTAGGAAAAGAAAACTCGATCAAATAATATCGCTCGCGGACGAAGTTAAAC

AAATGAGAGATAGAAAACATCGTCTGCTCAGGGAAAGGGATTACATGGTCGCCGAACGTTTGAGAGTAAA

AGAAAAATTTAGTCAACTTTACAGACACGTTTTTCAAGCGTTGAGAGATCCCGAGGGAAATCAATATTCC

CCGTACGAGTACAGCCTTCAACAATCCGCCGACGGCACGGTCGTACTCGTACCGAGATCAACGTCGAACA

CATTATTGGATCAAGATGGTGGTACAAACAGAGTCAAACACGGGAAAGATCAAAATCATCACGAAAGTCA

TCAGCAAAAGGAATGA

>cn_h_vulgaris lcl|XM_002160512.3_cds_XP_002160548.1_1 [gene=LOC100209530] [protein=uncharacterized protein LOC100209530] [protein_id=XP_002160548.1] [location=114..1721]

ATGATTTGGGACCAAGGTGTAAGTAACTTTATCGTAGCTTTATTGTTCACAAAATGGATTTTACAATCTT

CAAATGACACTCTGCAAATAAATCAGATTTTTAATAACGTTCCGTTCAATAAGAGACTTGTTTCAACATT

TAGAAATCTTTATAAAAATGCTGAAAATACTCAATTAGACTTAAACATAGCTGGGTACCATGATCTGCAA

AATTTAAAGGGTTCAAATGTTTATTCATATGAAGTCCAAAATCTTTTAAGTTGGCAAAATGCATATCATA

GAGGCGATCTAAGATTGCACTCAGAATTGCAGTTGAGTATTTTACCAGTCAATGCTGTGCATAAAGGCGA

TAAAAAAATTAGCAATTCATTACAAACAATTTCTAATGTATTTATTGATGATGGGTATGATTCAAGTGGA

TTGAGTTTAGATTCTCCAACATCTAGCACATTATCAGAAACCAGACCTGATTATTTATTTTCATCCTCAT

CACCGCCATTCTCTGATAATAATTTTGAAAATTTTAGTAGCTTACCTGGAGCTTATTCAGCTGATTTTGA

CATAGATGATCTTCTAGATAATAATGATTTAAATTTAGGTGTTGAAACAAATTACAAATCTCAAAGTTGT

TGTAATGAAAAAGAAAAATTAAGTTGTTTAAATCCATTTTCAAAGATATTAGATATAGATCATGATTTGG

ATTATGTTTCTTCCCCTCTTAAAAGCCCATCATTTGATGAGTTTTTAACAAGTCCTAATCAAGGCTTTTT

AACAAAATATGGGATTGATATATCTTTTGAAGACCCATTTGAAATTTGTTTCACTAATTCATCTTTAAAA

GAAAAGTTATTAGATAGTTCATTAAATATAGAAGACTATGATATAAATGAACTAAAGCTAGAAGAAGATG

TCGATAGAGCTCTAGTAGAGTCATTATCTCCTAATGTATCAAAATACAAAGTAATGAATGATGAAAGTAA

TGAAGTATCAAAAGCGCTCATTGAAAAAAATGCATTTGAAGTTGATCATGACTACTACCAGAGGTCAGAT

TCAAGTTTAAATTATACAACTAAAAGTTACCTAAATGACGTTGCAGTAGGTGCCAGGCAGTTAACAGAGT

TATCTCATACCTTTGCTCCATCTCATTCTGTCACAAACTCTAAGGAAACAAAATCAGAAAATATATGGCC

TGATTTTCCATACACATCAGAAGAACTTGTTACTATGCCAGTTGATACTTTCAACGAAGTTATTAAATTG

TTAGATGAAATCAGAAAACACATTGCAAAAGATGTTAGAAGAAAAGGAAAAAATAAATTTGCTGCTCGTG

GTTGTCGTAAAAGGAAAAATGATCTAATTAAGTGTTTAGATATTGGCGTAGACGAACTTATCAGGAAAAA

AAACAACCTATTAGATGAAAGAAATAAAATCATTGCAGAAACTTTAGAAATTAGGAGAAAAACAATGTGG

TTAAACAGCTACATATTTATGCATCTGAGAGACAGTAATGGAGGACTATATTCTTCTGTTGATTACTCAT

TACAGTACACATCTGATGGTAATGTTTATATTGTTCCGAGTGACAAAACAAGCAAAGTACACATCTGA

>ne_c_elegans lcl|M84359.1_gene_1 [gene=skn-1] [location=join(459..597,760..1184,2492..2813,2867..3019,3689..3947,4101..4468,4520..4593)]

GTCCATATTCATCTGATACTTGCAACATCATCACTGATTTTGGTGATCAGTTCACCATCGTCCAACACCT

CAATCCAATCATCGTCATACGATCGGATCACGACAAAACATCTTCTGGACAATATATCACCGACATTTAA

AATGTACACGGACAGCAATAATAGGAACTTTGATGAAGTCAACCATCAGCATCAACAAGAACAAGATTTC

AATGGCCAATCCAAATATGATTATCCACAATTCAACCGTCCAATGGGTCTCCGTTGGCGTGATGATCAAC

GGATGATGGAGTATTTCATGTCGAATGGTCCAGTAGAAACTGTTCCAGTTATGCCAATACTCACCGAGCA

TCCACCAGCATCTCCATTCGGTAGAGGACCATCTACAGAACGTCCAACCACATCATCTCGATACGAGTAC

AGTTCGCCTTCTCTCGAGGATATCGACTTGATTGATGTGCTATGGAGAAGTGATATTGCTGGAGAGAAGG

GCACACGACAAGTGGCTCCTGCTGATCAGTACGAATGTGATTTGCAGACGTTGACAGAGAAATCGACAGT

AGCGCCACTCACTGCCGAAGAGAATGCTCGATATGAAGATCTTTCGAAAGGATTCTATAATGGATTCTTC

GAGTCGTTCAATAACAATCAATATCAGCAGAAACATCAGCAACAACAACGAGAACAAATAAAGACACCAA

CTCTTGAACATCCAACTCAAAAAGCCGAATTGGAAGATGATCTGTTTGATGAAGATCTTGCTCAGCTTTT

CGAGGATGTTTCAAGAGAAGAAGGACAATTGAATCAACTTTTTGATAATAAGCAACAACATCCAGTTATC

AATAATGTTTCTCTGTCGGAAGGAATTGTTTATAATCAGGCAAATTTGACCGAGATGCAAGAGATGCGTG

ATTCCTGCAATCAAGTTTCCATTTCAACAATTCCAACAACATCGACTGCTCAACCAGAGACTTTGTTCAA

TGTAACCGATTCACAGACTGTCGAACAGTGGCTTCCAACAGAAGTTGTACCAAACGATGTGTTCCCAACA

TCCAACTACGCCTACATTGGAATGCAAAACGACAGTCTTCAAGCAGTTGTATCAAATGGACAGATTGACT

ATGATCATTCCTATCAATCCACTGGTCAGACTCCACTGTCTCCTCTCATCATTGGATCTTCAGGACGTCA

ACAGCAGACTCAAACGAGCCCAGGAAGCGTCACAGTGACTGCAACAGCTACTCAATCGTTGTTCGATCCA

TATCACTCACAGAGACACTCGTTTAGTGATTGCACTACTGATTCGTCATCAACGTGCTCTCGCCTCTCTT

CGGAATCTCCACGATACACGTCAGAGAGCTCAACCGGAACTCACGAGTCTCGTTTCTACGGAAAGTTGGC

TCCATCCAGTGGATCACGCTACCAACGATCATCGTCTCCACGTTCATCACAATCTTCGATTAAGATCGCG

AGAGTTGTTCCACTGGCCAGCGGACAACGGAAGCGTGGACGTCAATCCAAGGATGAGCAGCTCGCCAGTG

ACAACGAGCTTCCAGTGTCGGCGTTCCAGATTTCGGAGATGTCATTAAGCGAGTTGCAACAAGTGTTGAA

GAACGAGAGTCTCAGCGAGTATCAAAGACAGTTGATTCGCAAGATTCGTCGACGCGGAAAGAACAAGGTT

GCTGCCCGCACTTGCCGTCAAAGACGCACGGATCGTCACGACAAGATGTCCCATTACATC

>pl_t_adhaerens lcl|XM_002116532.1_cds_XP_002116568.1_1 [gene=TRIADDRAFT_60616] [protein=hypothetical protein] [protein_id=XP_002116568.1] [location=1..1959]

ATGCTTTCAGCAACGGATCTACAACAGTTCATCGTCGGTATTTTAACCGATGTAGCAACGTTATATGTTT

GCGCATCAAGACAAGAACTGATGCCATATTTAAAATTGGATGACAATGTCATCCATAAAATGAAATCGCC

AATGTGGGATGAAGCTGTAAAAACGATGAGTAAATATCCACAAAGTACTTTACAATTTCCTTCGGGTTAT

CATAACAGCTTTTCACATAATATTCACTGGCCAACTCAAGCGGTTGTTGCTTTCTTTGATGTGGTACCCG

ACGAACAAAATATCAACGAGCAGGCTGACAACGGCAACAGCAATGAAATGCCGAATGAACTTGCCAATGA

TGATGAAATGGATGATATTTTCTCAAATGTTGAGGAAGAAATTACCACAGACTACAATACCAACTTGTTA

ACTCCTGGAGAACTATCATCAGCTGTTCTCTCCATGAAATCCAATACCACGGGTGAATTCGATTGGGATT

ATTGGCATTTTGGTAATGATGACACCCCCAACATGCTGCCATTAAATGGTTGGAAAGGCGATGACAATAA

AGGCATTGCATCATTGACTGGCAACGCTGTCAATGGTCTCAACGATGAAGACTCATTCGCGTTAGCTAAT

ATCGTTGATGATGATGATAATAACAATTTGGGTGTGAGTGAAGATTGGTGGGGGCAATTGCCGACTGATT

TCGTCGATAGCCCAAGCGAAGGATGTAGTAATGGCTATAGAAGTGACGGCAATAATACCGATCAAAGTAT

CTCACCATCTGTGATTGCTGCCGACAGCGACAGCAACAGTCGTAGCGAAGCATGGGATCAAATCAAGAAA

CATATCAATGATGATGAGGTTCTACTTCACTCTATCAATAGCTTAGCATCTCATCAAAGTTCGCAGGGAT

TTCTACCATTTATTCCTCCAAATATAGCAGATATTAGCCCTATTTCTCGTAATGGATTTAGTTGGGATCA

TTCTGGACAAGATGATGCATCTGGCTTTAATACAAATCAAAACGATTTATTTCACGAATTAGGATTAGAA

TTAGATGTTCTTGGTAGTCAAAGCAATAATGAAGCTTCCTTACATCTTAATAGAGATGGACCGCTCAGTA

TCTCGACCACGAATGATAATCATTTTCTACATCATGGAATACCCAATAGTTCTTTTAATAATGCCACCGG

TAATCCTTATGGTGAGTTCTTTGATCTAATGGGTTTAGATAATCAATCTCCAACTGGCGAATTAAAAGGC

GACTTACCTTTCCAACCAATACAAGCGTCCAGAAATGGAGTTGCTTCAAATGCTAGCCAACCTCATGTCC

CACCTTATAGCAGTAGCTCATCCTCTTCAAGGGATGCAGTATATGCGTTATTTACCGATAGCCGCCCTTA

CGAATGTAACCCTCAAACCATGGGTGCAACGATGGACATGGGAACTTTTTTCGGTTATAACAATGATGCT

ATTCGGCTAGAAAATTCCATGGTTGATTCTCATCAGGTTGCCAGTCAAAGTCAGCCTATTGGCGAATGGG

CGTCTGCTAATGGGACTGTTGTTTATGGTGAGACGCAAAGCGAATTTGCTGAAATATTTGGTGTACCAAC

CAATAGCATGTTAGTTTTACCAGTACCTGAGCGCGAATTAGTTGACATGCCTGTTAATGAGTTTCTAGCC

ATGATTGAGCGTTTACCCAGTGATGTTGCAGCCCTTGCTAGAGATGTTAGGAGACGTGGCAAAAATAAGT

TTGCGGCTCGTAACTGTCGAAAAAGAAAGATCGACGACATAGATGGCTTAAAAGATGAAGTAGACGAATT

AGAAGTGCAGAAAGAGAGTCTGCTTGCAGAAGTTAAAAAACTCGAAGAAGAAAGCGAGAAATATCGTAAA

AAAAGTGAAGCCATGTACAAGAAAATCGAAAAATATGTAAAAATGAAGGAAGCTAACAATCGATCTTGA

>po_a_queenslandica lcl|XM_011404248.1_cds_XP_011402550.1_1 [gene=LOC105311980] [protein=uncharacterized protein LOC105311980] [protein_id=XP_011402550.1] [location=1..1728]

ATGGAATCATTTGAGATACCATCCACTATCAAACAGGAGCAGAATGTCCTGCCTCCTAGTAAATTCAATA

CTGGGTTTGGTCTGCCCGTTGGCAGTAATGGAGGGGTTCCTTCTTACAGTAGCCCCTCTCCTCCTTTCAT

GCAGTATGAAACTGGCATATATGTACTACCCATTGATCAACTCTCTAACAATCCTTACAAGCAAGATGGA

GGGGAAGACGACAGCCAGTCCTGGCTATTTCGAAAGTATCCAACAAGCAGCTCTGGTGATGCGAGCTGGC

TGCCTCCAGTCACTTCTGGAGACGATTTTAATAATGTCCTTTCTCAAGTTCCTTCGACTTCCTCCTCCTC

CTCTTTCCCTTTTGCTAAAGAAAAGACTGTCGAGAAAGACGAAGACTTTCTCGCTCAGTTGCTAGACGTC

GGTGGGTTTCTTGATGGATTTGACAATGACTCTTTTTACAGCGCGTTCAACCAGTCAAACGAGTTATCAA

TACCGGTTCAGCCGGTTGCCGGTGCGATCCCACCGCTGCCTAATCACCCGTCGCCTCCTCAGTTGTCCCC

AATGCAACTGATTCCCTCATATCAGCAGTATGACATACCCCAGCCACTGGAGGCCAATACAGAGTATAAC

GGGCTAGAAGACCCCGGACTAACTGTTCCAATAATGGCAGAGGAGCATGATCCTCTTGTTTCTGGTGTGA

ATGAGGTCATGCCTTCTCCTAGTAATGAGGTTGTTCCCATTTCAAATGAGCACGTAATTGCTCCTCCAGC

CGATGATCTCCAGGATCTTCTCGGAGAGCTTTTCGGGAAAGACGATTTCTCTTTATCAAACTCTTTAGTA

GCGCCTCAAACTGAGCGTAATGTCATCCCAGTCGGAGGGAATGGAGAGGCTTTTAATAATTTCGTGAGTC

CTTCCTTTATTCCTCTCTCCTTTTCCCCCACGTCTGACAACGAGACCAACCAGACACGAAAAACCGAGGA

CAATATGATACCGTCTGGTGACCTCTTGCCAGTGTCTAAGAAGCACCGTGGTACATCGGAGAGCAGCTGC

AGCACACTGGCCTCTTTACTTCTAGAGGAGCGACCGATGGACATTCAACTGGACGATGAGCCGAAAAAGA

AGCCGTCACTTTCGTCACCTGTTAGCTCTCCTTCTCAATCCTCTCCTAAAGACGGTCCGGCTCCCTCATC

GAGTCGTAAGTCACACAGTGGAGGAAATGGAATAGGAACAGTCGCCATGTTTGGACAGAACGAAGACGAG

ATTATACATAAGCTCATGTCTTCGCATAATCGAAGAGGAGGAGCGTCGAAACCAATCACTCGGGACAAGC

TTGTCATAATGCCCGTTGAAGACTTTAACAGTTTGCTGGACGAAGCACTCCTGAGTGAAATTGAAGTGGC

CTTCATGAAGGAATGGAGAAGAAGGGGAAAGAACAAGATGGCTGCTCAGATAGCGAGAAAGAGAAAGCGA

GAAGAGCTATTTGAGCTAGAAGACGACATGGACTCCTTGAGACAGAAGAAGTCAAAACTCCAGCAGAGCG

TGGCAAAACTAAATGCCCTCATTGCCTCGTATAAGAGGCGGGTCGAAGTCGGCGAGAAAAAAATTTACGA

ACGATATTCTCACGCCCACGGCTCGTTAGTCTCTCGTGAGACTCACACCATCCACATGACAGATGATGGC

AAAACTATGCTCATTCCGAGAACTTCAGATCAAGTACTTCTAGTTTAA

>f_b_fuckeliana lcl|XM_001545911.1_cds_XP_001545961.1_1 [gene=BC1G_15568] [protein=hypothetical protein] [partial=3'] [protein_id=XP_001545961.1] [location=1..>1761]

ATGAACTCTATCAACGTGGCCGCAAACACTACAATGGATACCGCTCTCGAGATGACAAGTCCCGGCCGTA

AGGCCAAGCTCGCGGAAACCATTCAATCCATGGCAAACTTGAATAGTAACATTGAGAACATGGAATCCCC

TATGGATCCGGATGCCCAGGCAACAATCACAGACTTTCTCGATTTTACGGAATATCTACCTTCGGATTTG

GTTCGGTCATTGGCACTTATTAGTGACCTCGATGAGAAATATGTCAATGCTTCGTCGAGCTTGAATGATT

TGACGAAAACCTATGGTCACCTCCCCGATCTTCCTGCCGACACAAAACCTGACCCTGTCTCTCTGCGAAA

AGACATATCGACCAGCTTGACTGATGCTCTCGGTGCGCGTACTTTGGCTTTCGCAGAAGCATCACATCTG

GTTGAAAACGTGGAGAGACATTACAAAAGGGCCCAGAATATATTGGCGAAGCTTCAAACCATGGCCGAAA

ACTATCCCGAATCTCGAGAAACAAGTCCTGTTCCGCAGAAGCCAAATTCACCTGTCGCAAATCGTGTGCC

AGGAAAATTACTTTTGCGAACTGGAAACCCTGACGGTCGCATGCGTGTTCGCAAACGACGCGCGCCAATC

ATCACTGTTCCTGGTGAGGTCATGGCTCCCTATGAGCTCGACTACGATTCTTATGATTCGTGCAGTGATG

ATTCGGAATCCGACGCTGGTCCACCCACACCACGTCGGCAGACACCTGCGAGATCCATATCAGTCAATCC

TAAGATCAAGTTGAAGGTCAAGCCACCTAAGAAAGAGAAAGTACCAAAAGCCCCCCGGACACCAAGGCCA

CCTGGAGTAATGGGAACAAACGTACATAGCGCGGTCGCCGGGATATCGACAAGTAACGCACTAGCGAAAC

TTCAGCCTCCACCACCGGATGCGAAAGCGGGAACCGAAGATGCGCCTTGGCTTCAGTTGACCGCATGGGA

GCTTGCGAAGCTCAGAAAGAGAATGAAAAAGAATGCGGTATGGAGCCCGAGCGATACAATGATCGCACGT

GAATTAAAACAATTGGGACGAGGAATAGAAGCGTATCGTACTGCAAAGAGTAAGGCTGACGCTGCAGGAC

AGCCTTTCGAACAATCGGTACCACCCCAACTCACCGGGAAGACAGTTATCGCGGAAGGTGCTATAAGTGC

AGAAGCCTTAGGCACAGAGGAGATACAACTTAGCAATCGTGTAAAGAGCCTTTTTGGGAATCATCCCGAA

GACGAGAAGAAAGAGAAAGAAAAAGAAAAAGAAAAAGAGAAAGAGAAAGAGAAAGAGAAAGAAAAAGAGA

AAGAGAAAGAGAAAGAGAAAGAGAAAGAGAAAGAGAAAGAAAAAGAGAAAGAAAAAGAGAGAGAAAAAGA

GAAAGGGAAAGAAAAAGAGAAAGAGAAAGAGAAAGAGAAAGAGAAAGAGAAAGAGAAAGAAGAGGAAAAG

GAAAAAGAAAAAGAAAAAGAGAAAGAGAAAGCTCCTACTAAAACACCTAAGAAGCAGCCAGCATCGAAAA

AACGAAAACGCGAGTCTACCATCGAAGACGTAAAGGCGAATGGCGACGCAAGCTCGTCAACTATAGATGG

CATAACTGACATCAATGATGCTCAAGGTGATAAAGTGAATGAAGTAGATTCTTCAAAATTAGGTAAGCCT

CAATTCAAGAGAACGAAGACTGAAACCCCGGTTCCACTCCCTCATCCCATTATCACAAATGCCACTCCCC

GGGCAACTCCT

>f_t_blattae lcl|XM_004181184.1_cds_XP_004181232.1_1 [gene=TBLA0F01710] [protein=hypothetical protein] [protein_id=XP_004181232.1] [location=1..3759]

ATGGCTGCTGTTAAAAGAAAGGCTAGTCATGATTCTGATGCTTCTGATATTAAAAGCAAAACTAATATTA

AACTAGCTAAGATAGACAACTCAAAGCTTAAAAATAATCAAGAAGTAGAAAAAGTTAAAGATCATGAGAA

AGAAGAAGAAGAAGAAGAAGAAGAAGAAGAAGAAGAAGAAGAAGAAGACAACGAAGACAACGAAGACCAA

CTTAAAGAAGGAAACTCAGATAAAAAACCTGCTTCAAGAACAACTGCTCAAGATATTCAAATAGCTAGAG

AGACAGCTGAATTATTTAAATCTAATATCTTCAAATTACAAATCGATGAGTTGTTGAACCAAGTTAAAGT

TAACCCAAACCATGTTTCAAAGCTAGAAAAATTCTTACATAAACTATACGACGTTCTACAATTAATTCCA

GATTGGAAAGAATCGTCTTTAGCAGATGTAGAAACTTATTTCGATGATAAAACAGTCAAGATTCCATTTG

CCGATCCAAAACCTATTGCATCTTCCACAAACTATAAATTTAATTATAAATCTCCAGACCAAGTGTCTCT

AATTGGTTCATTCCCATTAAAGACTTTTATAAACCAACCAAATGGCAATTCCATAGATATTTTATTAACA

ATGCCCATAGAGCTGTTTGATAAAAAGGATTTCTTAAATTTTAGATGTCTTCATAAGAGAAGTGTTTATT

TAGCTTACTTGACACATCATCTATCTTCATTGTTAGCAAAGGATTCTTCTTTAAAAGATTTATTAAATTT

GGAATACACCTATCTTAATAATGATCCACTATTACCAATTTTAAAATTATCATGTTCTAATGAAACAAAT

TCTAAAAAAGAATCTCCCTATAATTTCCAAAAGACAAATTTCTCCATAAATTTAATCATTGGCTTTCCAT

TTAAAGCATTTGATACTAAAAAATTATCTCCCAAAAAAAATTGTATTAGAGTAGCCATAGAAAAGGACTC

AAATAATAATTCTTCATCACATTCAGCATTACCTCCAACGTTATTATATAATTTCTCCGTTTTATCATCT

TCAAGCCATGAAATATATTTGAAATATTTATATAAAACCAAAAAAATCACCGAGTCTTTCCAAGAAGCTA

CAATATTAGGTAGACTATGGCTAAATCAACGTGGGTTCAGTTCAAGTTTAGCCCATTCAGGCTCATTAGG

TGGATTTGGTTCATTCGAATTTTCTCTATTAATGGCTGCTCTATTAAACGGGGGTGGTATTAATGGTAAT

AAGATATTATTGCATGGGTTTTCCTCTTATCAATTATTTAAGGGTGTTATCAAATACTTAGCTACAATGG

ATCTTTCTTCAAAGGGTCATTTACATTTCCATTCCATGCCATCAACTTCCTCATCAGATGATTCAACAAA

TGCTCATTTCCATACTTCAAAATACACAGAGGAATCTTTTAATTTCCCAACCATCTATGATAGATCAACC

AATATTAATATTCTAAGTAAAATGTCAATTGAGTCTTATAAGATTTTAAAACTATATGCCACTGAAACTT

TAAAGATGTTAAATAACGTGGTACAAGATCAATTTTCAAATGTTTTCTTAACTAATATCAATAAATTAGA

TAATATAAAATATGATTTAGTTTATGATTTATCATTCCCTGTAGCATCTCTAAAAGTTGCCATGAATGAA

TTGTATGAAGATTTTGGTCCTTTTGAAAGAATAAAATTTATAACTTTTGAAAATTTCTTGGTGGATAAAA

TTTCTAAAATCATCAAATTCTCCTTAGGTGATAGAATTACTACTTTCGAAATACAACTGTTGGGACAAAA

GTCTTCGTTCCCCATCACAAGAAGAAAAATTTATCACTCTAAAAATCTGATTGCCAATTTTACTGCAATT

AGAATCAAATTATTAACAAATCCAGCAGAATCGGAAAAACTAGTGACAAGAGGCCCAGCTCATTCAGAAG

AACCAACTGAAGAGGCAATTAATTTCAAAAATTTCTGGGGCATCAAATCTTCGTTACGTCGATTCAAAGA

TGGGTCAATTACTCATTGTTGTATATGGCAAACATCTTCATCTGAACCAGTAATTTCGTCTATTTTGAAA

TTTGTTCTCCAAAGTCACTTATTCGAAAATGTTACAATTAATGATACAATTACAAAACAATTCCAAGATT

TATTACCATTACCAAATCTTCCTGCAAGTTCTAAAACTTCAATTTTGAATTTATCAAGCTATTTCAATTT

GAAGAAATCTTTTGATGAATTATACAAAGTCCTTTTCAAAATGCAATTACCTTTATCAATTAAATCCATC

CAACCAGTTGGCTCGAAATTTAGATATACTTCTTTATGCCAACCTGTTCCATTTGCTTATTCTGATCCAG

ATTTCTTCCAAGATGTTATTTTGGAATTTGAAACCTCTCAAAAATGGCCAGATGAAATTACTTCTTTAGA

AAAGTCAAAATCTGCCTTCTTATTGAAAATCCATGAACAACTTAATACAGAACACAGTGATAAATTTAAA

TCGTTTTTCACTCGTGATGAATCAATTCCTTATAATTTGGAAATAACTATTTTAAATATTTTAACCCCAG

AAGGTTATGGTTTTAAGTTTAGAGTTTTAACTGAACGTGATGAAATCTTGTATTTAAGAGCTATTTCAAA

TGCAAGAAATGAATTAAAACCTGAATTAGAAAACACTTTCTTAAAATTTACTGCTAAATACTTAGCTTCT

GCTAGACATACAAGAACTATTGAAAATATTTCACACTCTTATCATTATTATTCTGCAACTGTTAGACTAT

TTAAAAAATGGCTGGATATTCATTTATTATTAGGACATTTAAGTGATGAACTAGTTGAATTGATTGCAAT

GAAACCTTTTGTTGACCATTCACCATACCTAATTCCTGGTTCTCTTGAAAACGGGTTCTTAAAAATTTTA

AAATTTTTAAGTCAATGGAATTGGAAGGAAGACCCTTTAATTTTAGATCTAATAAAGCCTGAGGAAGAAT

TCGAAAGTGGTTTTGAAACTAGTATTGGTGGTTCAGACTTAGATTCTAAAACATTAAAAAAGTTATCAGA

AAAACTTACATTAGCTCAATATAAGGCTATACAATCTAATTTTACCAACTTGAGAAAAAGTGATCCACAT

GGTTTAAACATTCAATTCTTTATTGCTTCTAAGATTGATCCTAGTGGTTTATTATACTCTAGTGGTATTC

CGCTACCAATTGCCACTCGTGTGACAGCATTGGCTAAAGTTGCAGTCAACATTTTAGAGACTCACGGACT

AAACAAACAAACTGTTGATTTATTATTTACTCCTGCATTAAAGGATTATGATTTTGTTGTCCAATTAAAA

GCACCAAAACCATTAAAAGCCTCTTGTGGTATATTGGAAAACACTGAATTCAAGAATTTATCTCAATTAC

CAACCAAATTTCCATCTGATTTAGATTCTATCTCTGAGAAGATGGATCCAACTTATCAGTTAGTTAAGTA

TTTAAATATGAAATACAAAAACAGCATTATCTTTTCTAGTCATAAATATATGGGGGCCAATGGTGGAGAA

AATGGAGATAAAAATGTTATTACAGCATTAATTAAACCAATGTTTAAACAAGACCATGCTTTTAAGGTGA

ACATTGATTGTAATATCAAGCCTGTCGATCAAGAACATGTTTCATTGAACAAAGAAGCAATATTCCATGA

AATAGCTGCCTTTGGTAATGAGTTTGTGGTTGAATTTGAAACAAAATAA

>bac_p_marinus lcl|CP000576.1_cds_P9301_05491_550

ATGCAAGAAAAACCTTCATCTTCCGAGAAAATATTTAACCTCGATAATCAAGCAAATAAACTTGGAATGG

GAGGTAAATTATCACCGGATAGCGATGAGAGCTCATATAAAAAAAGAATGCAGCAAAGAAAAGATATTCA

ATCAGAGAGACTACAAATTAGAAAAACAAAAAAAGGGTTATTGATTGTTTTTACAGGGAATGGCAAGGGC

AAAACAACTGCATCTTTAGGTATGGCTCTAAGGACGATAGGGCATGGCTATAAAGTAGCAATAATTCAAT

TTATCAAAGGAGGCTGGACCACTGGAGAAGAAAAAGCACTAAAAATCTTTTCTTCAAACCTATCTTGGCA

TTCATTAGGAGAGGGATTTACTTGGGAAACTCAAGACAGAATAAGAGATGAAAAATTAGTTCAAGAGGCG

TGGCAATTAGCCAAAAAATACATCCAAAACGAATCTTATAAACTTATCATTCTTGATGAAATTAATATTG

CGACAAAACTTGGTTATCTTGCACCCGAAGAAATAATCACTTTTTTAAAAAGCTTAAATAATAGAAAAAA

TCATATTGTTTTAACTGGAAGGGGAGCATCTGATTCAATTATCAATTACGCTGATCTAGTTACAGAGATG

AAACTAATAAGACATCCATTTAAAGAACAAGGAATAAAAGCACAAAAGTGTGTTGAATTTTAG

**References:**

1. Eddy, S. R. Accelerated profile HMM searches. *PLoS Comput. Biol.* **7,** e1002195 (2011).

2. Altschul, S. F. *et al.* Gapped BLAST and PSI-BLAST: a new generation of protein database search programs. *Nucleic Acids Res.* **25,** 3389–3402 (1997).

3. Gacesa, R. *et al.* Bioinformatics analyses provide insight into distant homology of the Keap1-Nrf2 pathway. *Free Radic. Biol. Med.* **44,** 1–8 (2015).

4. Bouckaert, R. *et al.* BEAST 2: A Software Platform for Bayesian Evolutionary Analysis. *PLoS Comput. Biol.* **10,** 1–6 (2014).

5. Notredame, C., Higgins, D. G. & Heringa, J. T-Coffee: A novel method for fast and accurate multiple sequence alignment. *J. Mol. Biol.* **302,** 205–217 (2000).

6. Wallace, I. M., O’Sullivan, O., Higgins, D. G. & Notredame, C. M-Coffee: Combining multiple sequence alignment methods with T-Coffee. *Nucleic Acids Res.* **34,** 1692–1699 (2006).

7. Di Tommaso, P. *et al.* T-Coffee: A web server for the multiple sequence alignment of protein and RNA sequences using structural information and homology extension. *Nucleic Acids Res.* **39,** 1–5 (2011).

8. Larkin, M. A. *et al.* Clustal W and Clustal X version 2.0. *Bioinformatics* **23,** 2947–2948 (2007).

9. Edgar, R. C. MUSCLE: Multiple sequence alignment with high accuracy and high throughput. *Nucleic Acids Res.* **32,** 1792–1797 (2004).

10. Katoh, K. & Standley, D. M. MAFFT multiple sequence alignment software version 7: Improvements in performance and usability. *Mol. Biol. Evol.* **30,** 772–780 (2013).

11. Chang, J. M., Di Tommaso, P. & Notredame, C. TCS: A new multiple sequence alignment reliability measure to estimate alignment accuracy and improve phylogenetic tree reconstruction. *Mol. Biol. Evol.* **31,** 1625–1637 (2014).

12. Jones, D. T., Taylor, W. R. & Thornton, J. M. The rapid generation of mutation data matrices from protein sequences. *Comput. Appl. Biosci.* **8,** 275–82 (1992).

13. Caspermeyer, J. New grand tree of life study shows a clock-like trend in the emergence of new species and diversity. *Mol. Biol. Evol.* **32,** 1113–1113 (2015).

14. Hedges, S. B., Marin, J., Suleski, M., Paymer, M. & Kumar, S. Tree of life reveals clock-like speciation and diversification. *Mol. Biol. Evol.* **32,** 835–845 (2015).

15. Wang, D. Y., Kumar, S. & Hedges, S. B. Divergence time estimates for the early history of animal phyla and the origin of plants, animals and fungi. *Proc. Biol. Sci.* **266,** 163–71 (1999).

16. Nei, M. & Gojobori, T. Simple methods for estimating the numbers of synonymous and nonsynonymous nucleotide substitutions. *Mol. Biol. Evol.* **3,** 418–426 (1986).

17. Tamura, K., Stecher, G., Peterson, D., Filipski, A. & Kumar, S. MEGA6: Molecular Evolutionary Genetics Analysis version 6.0. *Mol. Biol. Evol.* **30,** 2725–9 (2013).

18. Ronquist, F. *et al.* MrBayes 3.2: efficient Bayesian phylogenetic inference and model choice across a large model space. *Syst. Biol.* **61,** 539–42 (2012).
